# Rational Design and Functionalization of Melt Electrowritten 4D Scaffolds for Biomedical Applications

**DOI:** 10.1007/s40820-025-01986-9

**Published:** 2026-01-12

**Authors:** Yanping Zhang, Fengqiang Zhao, Aike Qiao, Youjun Liu, Menglin Chen

**Affiliations:** 1 Department of Biomedical Engineering, College of Chemical and Life Science, Beijing University of Technology, Beijing, 100124 People’s Republic of China; 2 Department of Biological and Chemical Engineering, Aarhus University, Aarhus, Denmark

**Keywords:** Melt electrowriting (MEW), 4D printing, Dynamic biomimetic scaffolds, Biomedical applications

## Abstract

Melt electrowriting (MEW) enables the precise deposition of polymeric fibers at micro-/nanoscale, allowing for the fabrication of 3D biomimetic scaffolds. By incorporating stimuli-responsive polymers and/or functional fillers, MEW-based 4D printing creates scaffolds capable of undergoing controlled, reversible shape transformations in response to external stimuli over time. These dynamic 4D scaffolds can be tailored for minimally invasive delivery, remote actuation, and real-time responsiveness to physiological environments, making them highly relevant for biomedical applications. This review systematically elucidates the principles of MEW-based 4D printing, including material considerations, actuation methods, and structure design strategies, along with shape programming and morphing mechanisms. The versatility of MEW for rational fabrication of biomimetic scaffolds is firstly introduced. Subsequently, the critical elements underpinning MEW-based 4D printing process are overviewed, including an analysis of stimuli-responsive materials compatible with MEW, an evaluation of applicable external stimuli, and a discussion on the advancements in design strategies for 4D scaffolds. Recent progress of MEW 4D scaffolds for applications in tissue engineering, biomedical implants, and drug delivery systems are highlighted. Finally, key challenges and perspectives toward material innovation, fabrication optimization, and actuation control are discussed. This review aims to provide valuable insights for design and creation of multifunctional biomimetic dynamic scaffolds by MEW-based 4D printing.

## Highlights

- This review categorically analyzes the state of the art of the structural complexity of melt electrowriting (MEW) scaffolds, ranging from 1D, 2D to 3D architectures, and presents advanced strategies to enhance scaffold quality.
- This review systematically elucidates the principles of MEW-based 4D printing, including material considerations, actuation methods, and structure design strategies, along with shape programming and morphing mechanisms.
- This review highlights the advances of MEW 4D scaffolds in tissue engineering, personalized biomedical implants, and drug delivery systems.

## Graphical abstract

**Figure:**
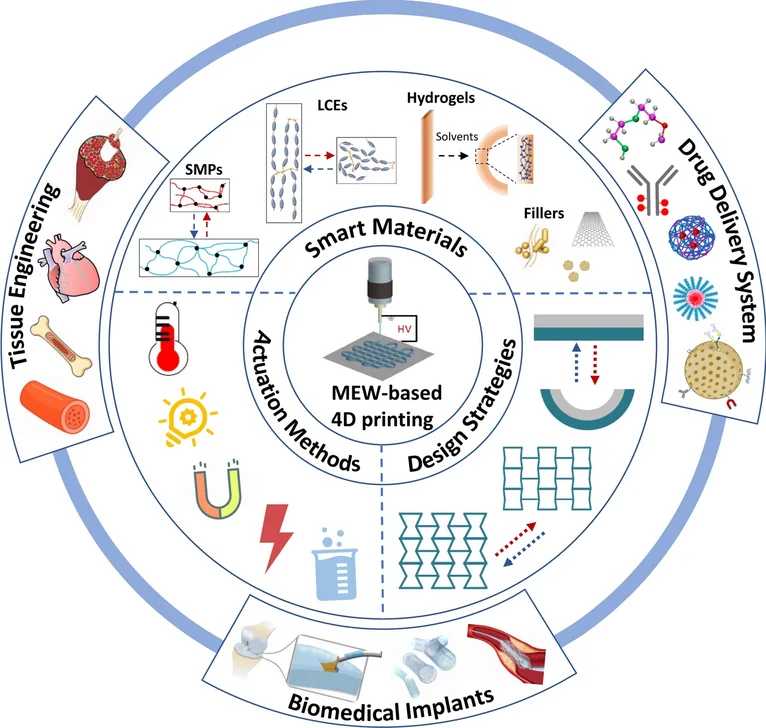

## Introduction

Bioimplants (e.g., tissue engineering scaffolds, biomedical stents) are designed to replace or restore damaged or diseased tissues/organs caused by aging, tumor resection, trauma, or degeneration [1]. They provide structural support, facilitate tissue repair, and promote functional recovery. Recent advances in biomaterials and biofabrication have enabled the development of bioimplants that better replicate the complex geometries and multifunctional properties of native tissues.

Although 3D extrusion printing has emerged as a powerful tool for the fabrication of patient-specific bioimplants due to its customization capacity, rapid prototyping, and cost-effectiveness, it is limited by strand widths of ~ 100–500 µm (constrained by nozzle size) and low positional precision, especially at small scales [2]. In contrast, melt electrowriting (MEW) [3, 4], an emerging technique that integrates principles from electrospinning with additive manufacturing, has attracted significant attention for its high resolution and versatility. Using high-voltage-assisted deposition of a molten polymer jet, MEW enables precise deposition of continuous fibers with diameters typically ranging from 2 to 50 μm, an order-of-magnitude finer than that of 3D printing, closely matching the scale of native extracellular matrix (ECM) fibers [5]. The absence of whipping instability ensures predictable fiber placement, and assembly into 3D porous scaffolds up to centimeter scale. Moreover, fiber diameter, pore geometry, and stacking layers can be finely programmed to map directly to cell-relevant cues. The MEW process is further enhanced by integration with filament-driven systems [6, 7] or UV modules [8, 9], broadening the range of printable thermoplastics and elastomers. With its unique ability to replicate multiscale architecture and biomechanics of native tissues, MEW represents a powerful platform for the fabrication of personalized, tissue-specific 3D scaffolds for biomedical applications.

However, traditional 3D scaffolds are geometrically static, lacking the dynamic adaptability of native tissues. This limitation has been mitigated by the advent of 4D printing, a disruptive technology that introduces a temporal dimension by incorporating stimuli-responsive materials (e.g., shape memory polymers (SMPs), liquid crystal elastomers (LCEs), hydrogels), enabling the fabrication of dynamic scaffolds that undergo programmable deformations respond to specific stimuli [10–13]. This dynamic adaptability is particularly valuable for bioimplants that must accommodate evolving physiological conditions, as well as their minimally invasive implantation in clinical practice. Recently, Liu et al. developed amphiphilic dynamic thermoset polyurethanes (DTPUs)-based 4D scaffolds suitable for minimally invasive treatment of tissue defects [14]. 2D laminated scaffolds composed of high-swelling (active) and low-swelling (passive) DTPUs were printed via fused deposition modeling (FDM), which could be fixed into temporary 1D roll-up shapes for catheter delivery. Upon implantation, body temperature restored the 2D pattern, and subsequent swelling induced programmed morphing into desired 3D structure for cavity filling and mechanical support. Additionally, Shi et al. reported a biodegradable elastomer (PCL-AD-4) with skin-like mechanical properties, and precise shape memory transitions at 37 °C [15]. 4D-printed PCL-AD-4 vascular stents, myocardial patches, and intervertebral disk scaffolds with negative Poisson’s ratios, all achieved compression/expansion cycles with excellent shape recovery. A hybrid biofabrication approach further integrated 4D-printed alginate-methylcellulose (AlgMC) hydrogels with MEW poly(ε-caprolactone) (PCL) meshes to fabricate shape-morphing, small-diameter vascular grafts [16]. These composites self-transformed into tubular constructs due to anisotropic swelling, while PCL fibers provided topographical guidance for cell attachment, and reinforced mechanics enabling suture ability and perfusion. AlgMC/PCL scaffolds supported co-cultures of fibroblasts, endothelial cells and smooth muscle cells, forming organized bilayers with reciprocal phenotype stabilization. Moreover, a multifunctional scaffold of hydroxyapatite, silica, poly(d,l-lactide-co-trimethylene carbonate), and Fe_3_O_4_ (HSP- Fe_3_O_4_) was fabricated for osteosarcoma therapy and bone regeneration [17]. Hyperthermia triggered chemo-, photo-, and magnetothermal effects while actuated shape memory effect of HSP-Fe_3_O_4_ scaffolds for defect-specific fitting.

These recent studies have increasingly emphasized the promising of 4D printing for creating dynamically responsive and temporally programmable bioimplants [10, 11, 14–18]. With this regard, MEW offers unique advantages for fabricating biomimetic scaffolds from SMPs, LCEs, and hydrogels that can undergo reversible and programmable shape transformations under external stimuli such as heat, light, or solvents. The incorporation of functional fillers (e.g., electroactive, magnetoactive, or photothermal agents) further enables remote actuation, mechanical reinforcement, and real-time imaging. Moreover, advanced programming strategies, including stress/strain mismatch, multimaterial layering, and spatial patterning, allow precise control of shape-morphing behaviors, yielding predictable tubular, curved, or other complex structures. Such 4D scaffolds not only support minimally invasive implantation and seamless tissue integration but also create dynamic microenvironments that enhance cell–cell and cell–ECM interactions, thereby promoting tissue remodeling and maturation [19].

Although several reviews have addressed the molecular design, functional properties, and additive manufacturing compatibility of stimuli-responsive materials [10–13], a systematic discussion of their integration with MEW is still lacking. MEW-based 4D printing remains in its infancy, and its clinical translation requires deeper insights into material compatibility, accessible actuation, and programming strategies. To address this gap, this review highlights the fundamental principles of MEW-based 4D printing. We first introduce the versatility of MEW for biomimetic scaffold fabrication, followed by an overview of stimuli-responsive materials compatible with MEW, applicable external stimuli, and design strategies for 4D scaffolds, along with their shape programming and morphing mechanisms. Recent advances of MEW 4D scaffolds in tissue engineering, biomedical implants, and drug delivery systems are then summarized. Finally, current challenges and potential solutions toward the fabrication of MEW 4D scaffolds are discussed to guide future directions.

## Rational Design of MEW Scaffolds

Scaffolds possessing biomimetic structural organization and adapting to biomechanics of native tissues are highly desirable for their use as biomedical implants. The versatility of MEW enables precise and customizable scaffold design, including control over fiber diameter, pore size, and pore geometry, allowing closely mimic the tissue-specific ECM architecture and mechanical properties. In this section, the state of the art of the structural complexity of MEW scaffolds categorized as 1D fiber diameter and morphology, 2D fiber micropattern, and 3D fiber configuration are overviewed, together with the advanced strategies for quality improvement of MEW scaffolds.

### 1D Fiber Diameter and Morphology

**Fig. 1:**
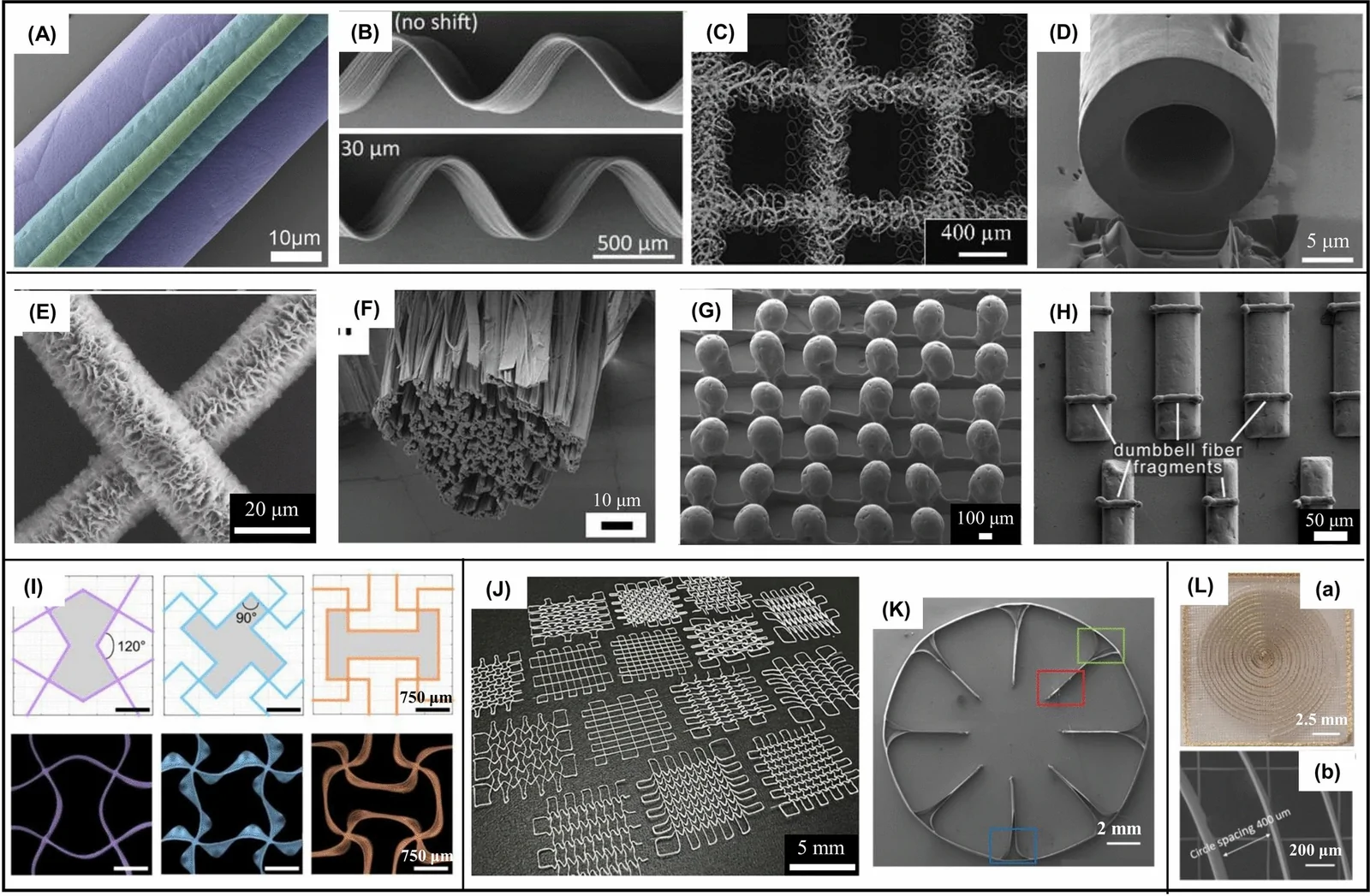
**A** SEM image showing stacked fibers with three different diameters. Reproduced with permission [21], Copyright 2018, Wiley Online Library; **B** SEM images of sinusoidal fiber walls. Reproduced with permission [28], Copyright 2020, Wiley; **C** Optical micrograph of MEW PCL mesh with entangled fiber morphology. Reproduced with permission [32], Copyright 2024, Elsevier; **D** Hollow fibers obtained with a coaxial nozzle [33], Copyright 2022, Wiley; **E** SEM image of PCL fibers after recrystallization. Reproduced with permission [34], Copyright 2024, Elsevier; **F** SEM image of PnPrOx fibril scaffold following dissolution of PcycloPrOx. Reproduced with permission [35], Copyright 2021, Wiley; **G** Coalesced spheres observed in MEW of PVDF onto heated collectors. Reproduced with permission [36], Copyright 2021, Wiley Online Library; **H** Fragmented PCL fibers. Reproduced with permission [37], Copyright 2022, Wiley Online Library; **I** Illustration and **J** Photograph of MEW-fabricated scaffolds with virous 2D micropatterns. Reproduced with permission [42], Copyright 2024, Wiley; **K** Circular scaffold with eight radial walls. Reproduced with permission [43], Copyright 2020, Wiley Online Library; **L** (a) Photograph and (b) SEM image of classic circular coil scaffold. Reproduced with permission [44], Copyright 2023, Springer Nature

In MEW, process parameters critically influence the morphology and diameter of deposited fibers, thereby determining the achievable resolution and consequently other structural and mechanical properties of scaffolds [20]. Fiber diameters ranging from 2 to 50 μm (Fig. 1A) have been achieved by modulating both melt feeding pressure and collector speed, without altering the applied voltage [21]. Optimized parameters (high voltage, low melt flow rate, small spinneret) enabled uniform PCL fibers of 817 ± 165 nm, forming a 50-layer crosshatch scaffold with 100 μm pores [22], while ultrafine PCL fibers of 275 ± 86 nm were obtained using an acupuncture needle spinneret [23]. The microtopography (roughness) of deposited PCL fibers became smoother with increased collector speed [24]. Notably, methodological models were developed for analyzing the effects of temperature, collector speed, tip-to-collector distance, melt pressure, and voltage on fiber diameter, enabling predictive optimization of fiber diameter [25, 26].

Collector moving speed above the critical translation speed (CTS) yields straight fibers, while speeds below CTS allow nonlinear toolpaths [4], such as serpentine/sinusoidal fibers (Fig. 1B) that exhibiting nonlinear mechanical properties closely adapt to native tissues [27–29]. Key process parameters (voltage, collector speed, tip‐to‐collector distance, spinneret diameter) govern the precise deposition of serpentine fibers [30], and spinneret geometry (convex, concave, flat) also affected their morphology, with a concave spinneret resulting in a reduction in sinusoidal amplitude [31]. Entangled fiber meshes (Fig. 1C) showed higher elasticity than straight fiber mesh due to untangling behavior at low strains [32]. Specialized fiber morphologies have also been fabricated, including coaxial hollow fibers (10 μm outer diameter, 6 μm lumen) (Fig. 1D) [33], PCL fibers with shish-kebab surfaces for osteogenesis (Fig. 1E) [34], collagen mimetic nanofibrillar microbundles from composite PVA/PCL fibers (Fig. 1F) [35], PVDF fibers with coalesced spheres (Fig. 1G) [36], fragmented microfibers via microrelief collectors (Fig. 1H) [37], and dual-nozzle systems for high throughput [38].

### 2D Fiber Micropattern

Together with fiber diameter and morphology, pore size and geometry significantly affect scaffold mechanical performances [39]. Larger pores reduce stiffness and increase yield strain, improving scaffold recovery after deformation. Electrostatic repulsion limits minimum inter-fiber distance, which can be mitigated by reducing fiber diameter and stack height [40]. Brenna et al. tuned PCL scaffold architectures (crosshatch, single-/double-wave, auxetic) (Fig. 1I) to achieve elastic moduli from 0.3 to 7.3 MPa [41]. Further complexity in scaffold design has been enabled through customized G-code generation (Fig. 1J) [42], such as circular scaffolds with radial walls for identifying preferred cell migration (Fig. 1K) [43], circular coil designs with precise spacing and stacking for wireless electrically stimulating neurons (Fig. 1L) [44].

### 3D Fiber Configuration

**Fig. 2:**
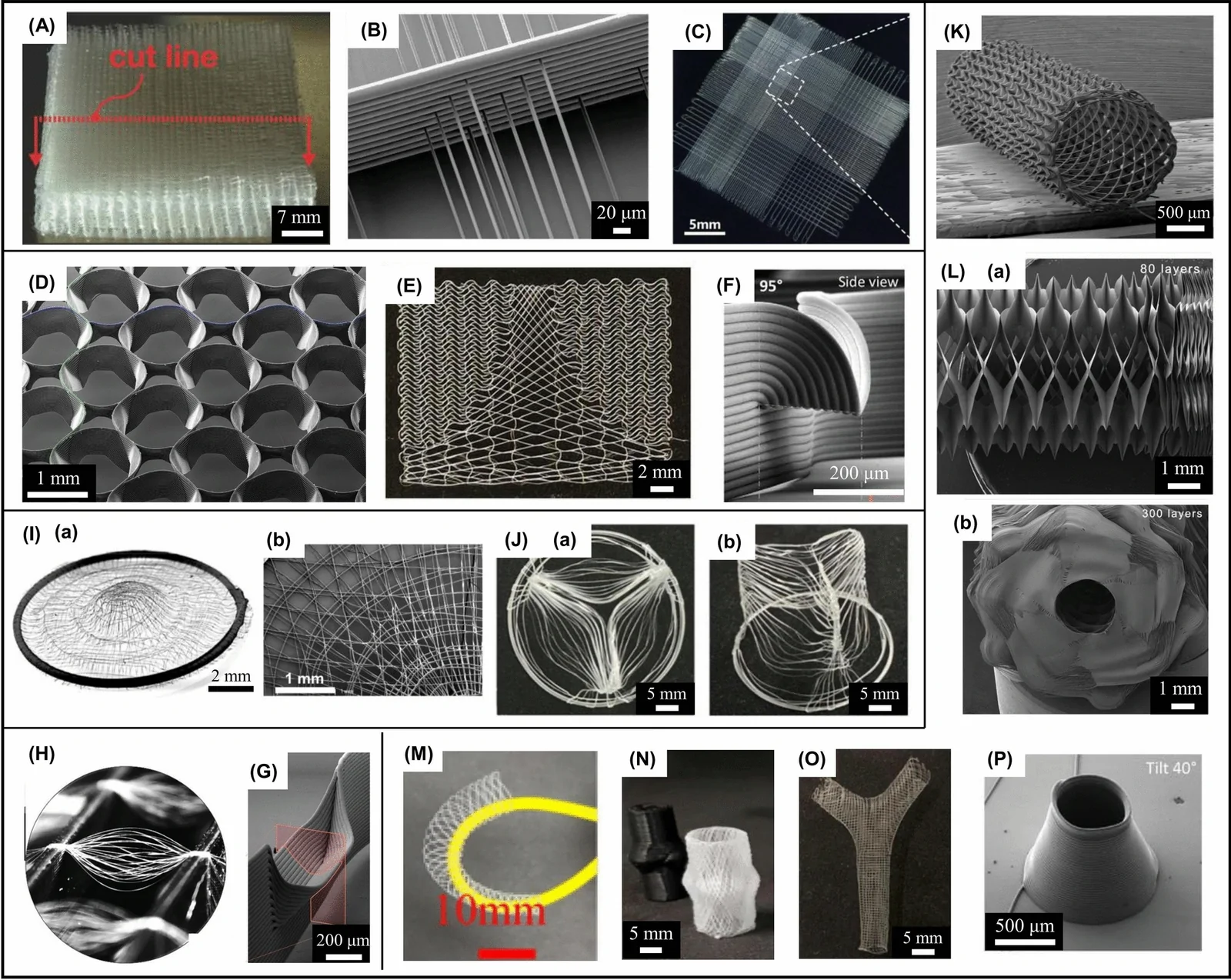
**A** Thick MEW scaffold (7.1 mm). Reproduced with permission [45], Copyright 2018, Wiley; **B** Suspended MEW fibers at varying heights. Reproduced with permission [49], Copyright 2021, Wiley Online Library; **C** MEW scaffold with gradient pore sizes. Reproduced with permission [50], Copyright 2019, Elsevier; **D** SEM image of MEW scaffold with circular pores formed by sinusoidal fibers. Reproduced with permission [28], Copyright 2020, Wiley; **E** Bioinspired MEW scaffold replicating the aortic heart valve interfacial region with continuous interfaces. Reproduced with permission [55], Copyright 2022, Wiley; **F** Overhangs enabling horizontal printing without support structure; **G** Fiber wall branching into dual overhangs. Reproduced with permission [28], Copyright 2020, Wiley; **H** Spindle-like constructs. Reproduced with permission [59], Copyright 2024, Wiley Online Library; **I** Funnel-shaped construct. Reproduced with permission [60], Copyright 2023, Elsevier; **J** (a) Top and (b) side view of MEW fiber constructs mimicking the aortic root with tri-leaflet valve architecture. Reproduced with permission [61], Copyright 2024, Elsevier; **K** SEM image of MEW tubular scaffold with a winding angle of 20°, 30 pivot points, 1.5mm internal diameter, and ~ 200 μm wall thickness. Reproduced with permission [64], Copyright 2018, Elsevier; **L** (a) SEM image of a tubular scaffold with compressed 300-layer and (b) side view of the scaffold. Reproduced with permission [70], Copyright 2021, Wiley; **M** Flexible tubular scaffold with gradually varying curvatures. Reproduced with permission [71], Copyright 2023, Elsevier; **N** Personalized aortic root scaffolds. Reproduced with permission [73], Copyright 2020, Frontiers; **O** Bifurcating vessel scaffold. Reproduced with permission [61], Copyright 2024, Elsevier; **P** Microscopic domes (“micropottery”). Reproduced with permission [28], Copyright 2020, Wiley

Electrical repulsion arising from charge accumulation within deposited fibers limits scaffold height beyond 2–3 mm. Height can be increased (> 7 mm) (Fig. 2A) by adjusting* z*-axis and voltage to maintain the electrostatic force at a constant level during printing [45]. Polygonal scaffold with up to 200 fiber layers and 9 mm height was accurately fabricated by maintaining a constant working distance [46]. Larger fiber diameters improve interlayer bonding, with a 5 µm increase in fiber diameter resulting in approximately 70% greater bonding strength [47]. Speed-programmed MEW produced 3D hierarchical scaffolds with tunable compacted coil densities, enabling spatial control over cell growth and density distribution [48]. Gradients in diameter (Fig. 2B) [49], pore size (Fig. 2C) [50], or multilayered architecture of scaffolds [51, 52], yielded their anisotropic mechanical behavior. By manipulating fiber spacing, orientation, and stacked layers, modulus values range from 5.6 to 13 MPa (tensile) and 6 to 360 kPa (compressive) have been achieved [53]. Orthogonal printing PLA and PCL fibers generated anisotropic composites [54]. Nonlinear geometries, including circular pores (Fig. 2D) [28], aortic valve interfaces (Fig. 2E) [55], overhanging structures (Fig. 2F), and bifurcating fiber walls (Fig. 2G) [28] also have been achieved.

Printing on non-planar collectors enables anatomically relevant structures. Strategies for accurate fiber placement on non-planar surfaces include optimizing size and material of hemispherical collectors [56], maintaining constant voltage and nozzle to substrate [57, 58]. Moreover, by leveraging fiber sagging and residual charge phenomena as part of the design intent, spindle-like constructs were successfully fabricated (Fig. 2H) [59]. Other designs include airtight, funnel-shaped tympanic membrane analogs (Fig. 2I) [60], and the tri-leaflet structure of the aortic root (Fig. 2J) [61].

The fabrication of tubular scaffolds with biomimetic microarchitectures is essential for engineering artificial tubular tissues [62, 63]. Custom tubular scaffolds have been fabricated using mathematical models to define winding parameters (Fig. 2K) [64, 65], and user-friendly G-code platforms to correct fiber lag during printing [42]. These strategies enable diverse patterns with tunable pore geometries [66–69]. Real-time correction of fiber pulsing facilitated the successful fabrication of a collapsible tube (12 mm wall) (Fig. 2L) [70]. Other designs include conical meshes (diameter tapering from 8 to 4 mm) (Fig. 2M) [71], patient-specific aortic root scaffolds (Fig. 2N) [72, 73], arterial structures [74], bifurcating vessels by employing multiaxis deposition (Fig. 2O) [61], and tapering dome-like forms (“micropottery”) by layer-shifting techniques (Fig. 2P) [28].

### Quality Improvement of MEW Scaffolds

Consistent scaffold quality remains challenging due to the multiparametric nature of MEW [75, 76]. A recent review has comprehensively summarized advances in improving scaffold quality, particularly in terms of fiber uniformity (printing stability) and fiber placement precision (printing accuracy) [77]. Key process parameters influencing fiber diameter/uniformity include voltage, melt flow rate, and collector speed [78, 79]. Conductive substrates improve precision via charge dissipation [80, 81], though no consistent correlation between jet landing accuracy and voltage configuration has been established [82]. Generally, increased fiber diameter and stacked layers, coupled with reduced inter-fiber distance, lead to diminished printing accuracy [83, 84]. A geometrical model incorporating coded trajectory, CTS, and collector speed was developed to predict fiber paths, enabling early defect detection and real-time G-code adjustment [85]. The jet lag effect [86] that affects the fiber placement accuracy can be compensated by a reverse speed planning process [87], while residual charge entrapped within pre-deposited fibers [88], particularly in narrow-spaced multilayers that compromise deposition accuracy can be mitigated by regulating collector temperature to control material polarization and charge retention [89, 90]. A charge-based analytical model grounded in surface energy evolution further clarifies the interplay between residual charge and fiber deposition accuracy [91], though charge neutralization alone yields limited benefits [92]. Additionally, based on an established mathematical model of trailing fiber trajectory, the jet lag effect was effectively suppressed by subdividing and optimizing the printing path [93].

## Smart Materials for MEW 4D Scaffolds

The cornerstone of 4D printing lies in the rational selection of stimuli-responsive “smart” materials capable of change their properties under external stimuli (e.g., heat, solvent, PH). According to recovery mechanisms, these materials are broadly categorized into conventional responsive materials, which undergo irreversible deformation after stimulation, and shape memory materials (SMMs), which restore their original configuration upon triggering. In biomedical applications, 4D-printed SMMs leverage programmed deformation and recovery to adapt to physiological environments, thereby enabling precise therapeutic interventions. In contrast, non-reversible responsive materials rely on expansion or contraction to achieve controlled morphological changes. Through the rational programming of material composition and spatial distribution within constructs, complex deformation behaviors can be finely tuned, allowing smart materials to interact dynamically with diverse tissue microenvironments and accommodate patient-specific anatomical needs. Representative materials include SMPs, LCEs, hydrogels, and functional fillers, which underpin applications ranging from actuators and adaptive biomedical implants to morphodynamic tissue engineering scaffolds [94]. Although several recent reviews have detailed their molecular design, properties, and additive manufacturing compatibility [10–13], a focused discussion of their integration with MEW remains absent.

Processing smart materials in MEW poses challenges due to stringent requirements for suitable melt viscosity, thermal stability, and low conductivity, as materials must remain molten for extended durations [95, 96]. PCL remains the most widely used, due to its low melting point (~ 60 °C), rheological stability, and biocompatibility [97, 98]. Nevertheless, ongoing advances in device update, fabrication optimization, and material innovation are rapidly diversifying the MEW material landscape. This section highlights recent progress in smart materials for MEW-based 4D printing, including SMPs, LCEs, smart hydrogels, and functional fillers, with emphasis on their suitability for specific biomedical applications. Table 1 summarizes reported smart materials, MEW printing conditions, scaffold structures and properties, stimuli, and their applications.

**Table 1:** Smart materials processed via MEW

Smart materials		Printing conditions	Structures	Properties	Stimuli	Applications	Refs.
Shape memory polymers	TPU powder (90A, BASF AG, Germany)	220 °C, 0.4 kPa, 12 mm min^−1^, 5 kV, 7 mm	Gird mesh with fiber spacing 100–500 μm, 1–5 layers, fiber diameter around 50 μm	High flexibility and elasticity, the breakthrough pressure of 3–150 mmH_2_O	–	Colorimetric sweat sensor, the average effecting time of 44.38 min, compare to the test paper (control) of 2.42 min	[115]
	TPU	24G, 220 °C, 200 kPa, 450 mm min^−1^, 4 kV, 3 mm	Scaffold with 72 °C tilting between layers, fiber diameter of 59 µm, 6 layers	Good adhesion between layers	–	–	[7]
	TPU filament (Filaflex 70A)	27G, 230°C, 600 mm min^−1^, 2.5 kV, 3 mm, then combined with electrospun TPU PBA-75 mats	Bilayer scaffold composed of MEW TPU layer with fiber diameter of 80 µm and electrospun TPU PBA-75 layer	*T*_g_: 60 ℃, E: 7 MPa, values of curvature: 85–1200 m^−1^ Rr: ~ 10–15% recovery per cycle	Heating (60 °C)–cooling (0 °C) cycles in water	Undergoing bidirectional shape changes in response to thermal stimuli	[116]
	SMPU pellets (DiAPLEX MM 3520)	25G, 200 °C, 25 kPa, 1500 mm min^−1^, 3.3 kV, 5 mm; then fused with hydrogels	Mesh with fiber spacing of 500 μm, fiber diameter of 58µm, thickness around 550 µm	*R*_f_ of 50–90%, Rr of 100% at 37 °C	Heating to 37 °C	Bestow temperature responsiveness to a hydrogel	[119]
	TPU PBA-75	27G, 215 °C, 100 kPa, 600 mm min^−1^, 3 kV, 2 mm	Lamellae structures with fiber diameter around 30–40 μm, distance of 500 and 150 μm, the height of 500 and 1700 μm	Exhibited different mechanical properties: hard state (E = 50 MPa, treated by cooling to 4 °C), soft states (E = 9 MPa, T = 70 °C)	Heating to 70 °C	A smart valve that be controlled by a single water droplet	[120]
	TPU PBA-75	27G, 215 °C, 100 kPa, 600 mm min^−1^, 3 kV, 2 mm	Lamellae with the height of 1500 μm, and coating with a photothermal ink layer (~ 0.9 μm thick)	Young's modulus for the coated lamella with and without light exposure was 42 and 6 MPa, respectively	Light	Light-controlled valves with a response time of 52 s	[121]
	EVOH pellets (K3850B)			*T*_g_	–		[125
Liquid crystal elastomers	LCE ink composed of liquid crystal mesogen (RM82 or C6M) and the chain extender (n-butylamine)	25G, 60 °C, 15 kPa, 420 mm min^−1^, 10 kV, 30 mm, UV cross-linking (365 nm, 100 mW cm^−2^)	Square lattice scaffolds with fiber diameter of 7 µm, fiber spacing of 300 µm, 50 layers, sinusoidal structures with a hexagonal design (fiber diameter of 16 µm, fiber spacing of 950 µm, 5 layers)	–	Heating to 120 °C	Exhibited reversible shape-morphing behavior, raise a steel ball (325 times its weight) to a distance of 3.7 mm when heating to 120 °C	[131]
	LCE ink composed of liquid crystal mesogen RM257 and the chain extender EDDET	26, 28, or 30G, 60° to 120°C, 0.2 MPa, 60–1200 mm min^−1^, 2 to 3 kV, 2 mm, UV cross-linking (365 nm)	Fiber diameter range from 4.5 to 60 μm	The maximum actuation strain of a single LCE microfiber actuator was approximately 55%	Heating to 120 °C	A maximum work density of 160 J/kg; spatial temperature field sensors exhibited a low response time (< 42 ms) and a high precision of 94.79%	[8]
Hydrogels	UPy-PEG	27 G, 120 °C, 0.3 MPa, 2100–2400 mm min^−1^, 3–3.5 kV, 3–5 mm	Grid scaffold with fiber diameter of 94 μm	–	A relative humidity of approximately 85%	A strain of 25% and 4.5% in the transverse and longitudinal direction, respectively, corresponding to a volumetric change of 64%	[140]
	Poly(2-ethyl-2-oxazine) (PEtOzi)	14 G, 150 °C, 0.15–0.2 MPa, 2–4 kV, DA cross-linking	Gird scaffold with fiber spacing of 500 μm, 10 layers	a Young's modulus between 0.14 and 0.2 MPa	Solvent	Fiber diameter increased from 45 ± 5 μm to 89 ± 12 μm, the total scaffold area increased from 4 cm^2^ to 9 cm^2^ after swelling	[141]
	Methacrylated alginate (AA-MA)	MEW PCL fibers were deposited on top of the UV cross-linked AA-MA layer	Bilayer scaffold composed of the dried AA-MA layer with a 125 μm thickness and uniaxial aligned PCL fibers with diameter of 22 μm	storage modulus around 9 MPa, the zeta potential of 65 mV	Solvent	Self-folded into a tubular scroll-like scaffold with anisotropic inner topography, allowing the growth of oriented muscle tissues	[169]
	Methacrylate hyaluronic acid (HA-MA)	MEW PCL–PU fibers were deposited on top of the UV cross-linked HA-MA layer	Bilayer scaffold composed of HA-MA layer and uniaxial aligned PCL–PU fibers with diameter of 2 μm and spacing 100 μm	elastic modulus of 8 MPa, withstanding stretching up to at least 200%	Solvent	Self-folded into a tubular scroll-like scaffold with anisotropic inner topography, allowing the growth of oriented muscle tissues	[170]
Electroactive fillers	0.1 or 0.2 wt% multiwalled carbon nanotubes (MWCNTs) in PCL	70 °C, 30 μL/h, 40 mm/s, 5 kV, 4 mm	Grid structure with fiber spacing of 100 μm, layer of 20	E of 6 MPa,	–	Good cytocompatibility for cellular spreading and proliferation	[146]
Magnetoactive fillers	0.3% USPIO in PCL	23G, 85 °C, 200 kPa, 360 mm min^−1^, 5 kV, 3.1 mm	Scaffold with wavy fibers (diameters ~ 33 μm)	Young's modulus around 250 MPa	MRI detectable	Real-time monitoring of the scaffold's performance	[152]
	SPIONs and (Si-SPIONs) coated on the surface of PCL fibers	85 °C, 255 kPa, 1680 mm min^−1^, 4kV, 0.35 mm	Grid scaffold with fiber diameter ~ 13 μm, fiber spacing ~ 300 μm	–	–	Exhibited antioxidant capabilities and enhanced cellular activity	[153]
	20% NH2-MIL-88B(Fe) MOF in PCL	23G, 75 °C, 300 kPa, 325 mm min^−1^, 5.4 kV, 5 mm	Grid scaffold with fiber diameter ~ 50 μm	–	MRI detectable	Silver induced excellent antibacterial efficacy	[154]
	30% Carbonyl iron (CI) particles in PVDF	26G, 200 °C, 300 kPa, 2800 mm min^−1^, 3 kV, 2.5 mm	Grid scaffold with fiber diameter between 30 and 50 µm	–	Magnetic field	Adhered to magnets and followed its movement; similar cell adherence and viability to pure PVDF	[155]
	10% Iron oxide nanoparticles (Fe_3_O_4_ NPs) in PCL	25G, 90 °C, 150 kPa, 400 mm min^−1^, 4.8 kV, 3 mm	Grid scaffold with fiber diameter ~ 10 μm, fiber spacing of 250 µm, up to 20 layers; tubular scaffold with decreasing radial fiber spacing from 850 to 250 µm in 50 µm steps	–	Magnetic field	Controlled magnetic responses (grip, “wings” flapping, preferential orientation, etc.)	[156]
	20% oxidized graphene platelets functionalized with iron oxide (rGNP@) in PCL	27G, 105 °C, 0.2 MPa, 120–600 mm min^−1^, 6 kV	Scaffold with hexagonal pores displaying a side length of 0.6 mm, fiber diameters of 20 µm, and scaffold thickness of 0.4 mm	Tangent modulus of 68 MPa	Magnetic field	Guided the formation of 3D myotube architectures, and undergo reversible bending	[157]
other fillers	0.1% Fluorescent nanodiamonds (FNDs) in PCL	23G, 100 °C, 0.15–17 kPa, 250 mm min^−1^, 6 kV, 4 mm	Grid scaffold with fiber diameter of 20 µm, fiber spacing of 100–400 µm	Young's modulus around 4.5 MPa	–	Real-time tracking of scaffold degradation; enhance cell proliferation	[160]
	4% Thermochromic dyes in PCL	24G, 80 °C, 240 kPa, 260–460 mm min^−1^, 6.3 kV, 3.5 mm	Scaffold with a 0°/45°/90°/135° laydown pattern with a 440 μm interspace	–	Heat	Respond to temperature by changing the color	[161]

### Stimuli-Responsive Polymers

Stimuli-responsive polymers, particularly SMPs, LCEs, and smart hydrogels, are central to 4D printing owing to their shape memory effect (SME) [99], which enables programmed deformation and recovery under external stimuli. Most current systems exhibit one-way SME, requiring reprogramming after each cycle, while two-way SME materials allow reversible switching without reprogramming, and multiple SME behaviors, achieved by tuning transition temperatures, copolymer design, or multimodal programming, expand biomedical potential for multifunctional implants requiring sequential or continuous shape changes [100].

#### Shape Memory Polymers

Shape memory polymers (SMPs) are the most widely investigated smart polymers, capable of reversible deformation under external triggers, most commonly heat [101, 102]. Thermoplastic SMPs, including polylactic acid (PLA)-, poly(lactic-co-glycolic acid) (PLGA)-, polyurethane (PU)-, and PCL-based, are extensively studied for biomedical applications due to their potential biocompatibility, tunable mechanical properties, adjustable biodegradability, and processability [2, 12, 103–107]. 4D-printed SMP implants have been explored in tissue engineering scaffolds [14], stents [108], and drug delivery systems [109]. Their SMEs allows compact delivery for minimally invasive implantation and subsequent recovery into adaptive geometries under specific stimuli upon implantation at the target site, enabling intimate conformity with host tissues.

**Fig. 3:**
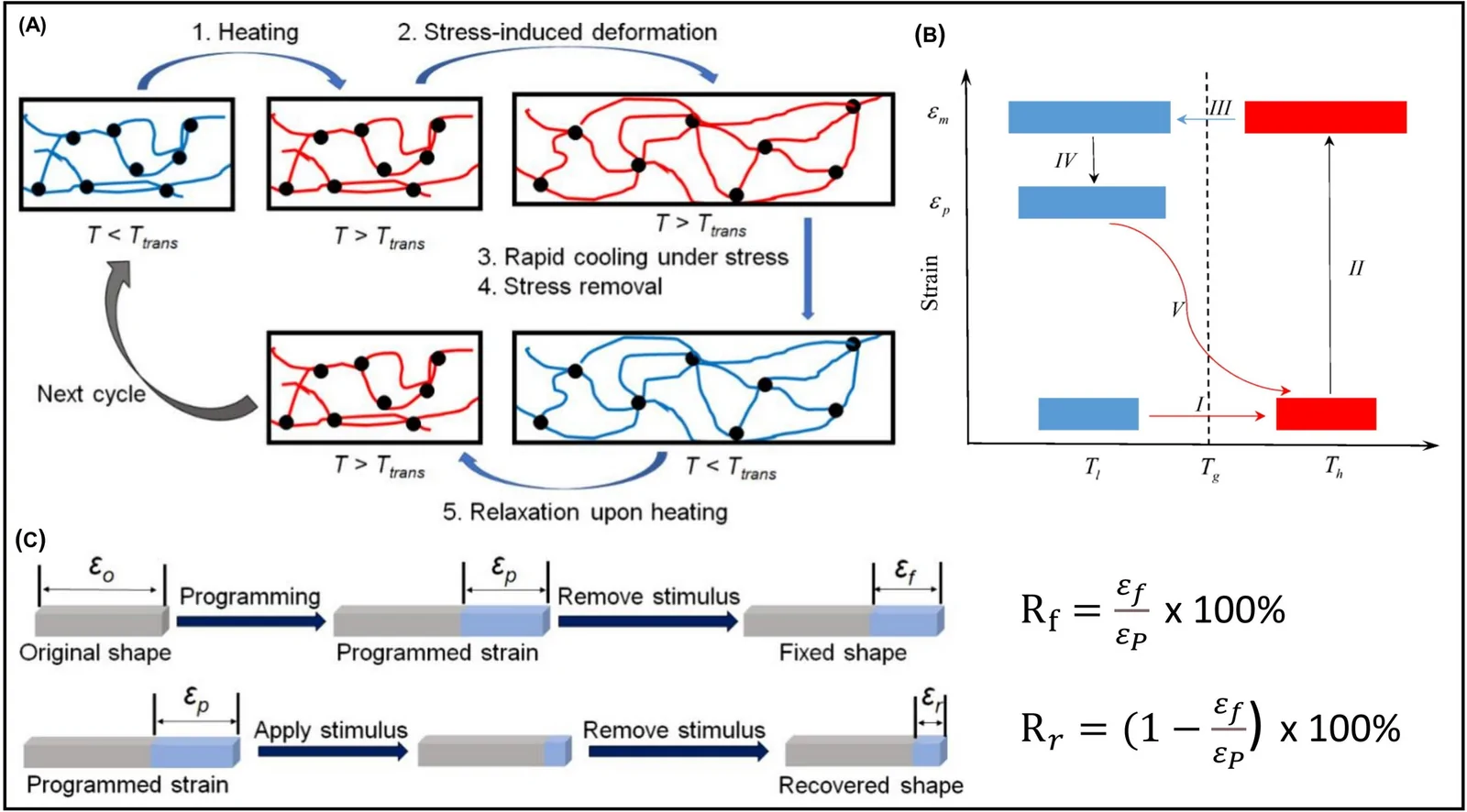
**A** Schematic of SME in SPMs: black dots represent net points, blue chains indicate rigid network below *T*_trans_, and red chains indicate the mobile network above *T*_trans_. Reproduced with permission [11], Copyright 2025, ACS; **B** Shape memory effect illustrated on the strain–temperature plane. Reproduced with permission [112], Copyright 2017, Elsevier; **C** Schematic of shape fixity and shape recovery, where *ε*_o_ is original strain, *ε*_p_ is programmed strain, *ε*_f_ is fixed strain, and *ε*_r_ is residual strain. Reproduced with permission [11], Copyright 2025, ACS

Shape memory programming, typically encoding stress/strain within SMP-based scaffolds, is the initial step and essential for enabling their reversible shape transformation [110, 111]. The detailed mechanism of thermoresponsive SMP programming and shape recovery is schematically illustrated in Fig. 3A. Below the transition temperature (T_trans_), polymer chains are immobile, and the material retains as a rigid plastic. Heating above T_trans_, the increased chain mobility allows macroscopic mechanically deformation with a corresponding entropy reduction. This temporary shape is then fixed by cooling the polymer below T_trans_. Upon reheating above T_trans_, chain mobility is restored and the polymer recovers its permanent shape by returning to the original high-entropy conformation. Figure 3B displays a typical thermomechanical programming protocol for SMPs in the strain–temperature phase diagram [112]. T_trans_ defined by melting temperature (*T*_m_) or glass transition temperature (*T*_g_), can be finely tailored by rational design of block length, segment composition, or phase separation [110, 113, 114]. Several quantitative metrics of SME behavior are critical for bioimplant design, such as shape fixity (*R*_f_), ensuring implant retain its programmed form during minimally invasive implantation, and shape recovery (*R*_r_) that guarantees full restoration of implants for seamless tissue integration (Fig. 3C). Shape recovery rate is also critical for clinical workflows.

Thermoplastic polyurethanes (TPUs) are particularly well studied for MEW [7, 115–118]. For example, MEW-printed TPU-based SMP (*T*_g_, 35 °C) meshes (60 μm fiber diameter, 500 μm square pores) embedded within hydrogels achieved good to excellent* R*_f_ (50–90%) depending on the hydrogel composition, and excellent Rr (∼100%) at 37 °C within 2 min [119]. Cytocompatibility tests demonstrated good viability either with cells seeded on top the composites or encapsulated within hydrogel during all shape memory processes. In addition, poly(1,4-butylene)-based poly(ester urethane) (TPU PBA-75), a physically cross-linked SMP containing 75% crystallizable PBA soft segments was processed via MEW [120, 121]. This material exhibited dual mechanical memory states at room temperature depending on their thermal history, “soft” following high-temperature exposure (> 70 °C) and “hard” after low-temperature treatment (4 °C), arising from the amorphous or semicrystalline states of the soft segments, respectively. High-aspect-ratio lamellar structure (fiber diameter 30–40 μm, height-to-width ratio 57:1) exhibited reversible changes in stiffness and deformability upon thermal treatment [120].

Most biomedical SMPs are thermally triggered, with T_trans_ adjusted near physiological levels (~ 32–37 °C) for passive actuation [122, 123]. Polymers with higher T_trans_ require external heating or solvent-mediated activation but must remain biosafe and biostable under physiological conditions. Cytocompatible, biostable TPU-based SMPs with high *T*_g_ (> 50 °C) synthesized via solvent-free methods demonstrated *R*_f_ of 73–80% and R_r_ of 93–95% even in degradation media, enabling stable fixation and user-defined recovery [124]. The degradation of SMPs, governed by hydrolyzable bond density, hydrophilicity, geometry, and porosity, must be tailored to match tissue formation [104, 105]. By rationally modifying molecular structures, SMPs with tunable degradation rates, hydrophobicity, and mechanical properties can be designed to meet specific application requirements.

However, processing biomedical SMPs via MEW remains challenging due to high processing temperatures, which can cause swift degradation and rapid jet quenching, impairing print quality. Besides the innovation of SMP-based materials with suitable thermal properties, the optimization of MEW processing offers an alternative strategy. For example, T. Sun et al. employed ethylene vinyl alcohol copolymer (EVOH,* T*_m_ = 174 °C) to investigate the principles of printing high-melting-point polymers through MEW [125]. The effects of melt temperature (180–250 °C) and collector temperature (60–100 °C) on jet motion, fiber stacking, and scaffold mechanics were systematically examined. Precise control of both parameters mitigated quenching and stabilized deposition, enabling continuous printing of EVOH microfibers (8–14 μm) for > 15 h with strong interlayer adhesion. Elevated collector temperatures slowed quenching, suppressed jet “jumping”, and promoted fiber bonding, allowing the fabrication of complex geometries such as tubular stents with favorable compression fatigue resistance.

#### Liquid Crystal Elastomers

**Fig. 4:**
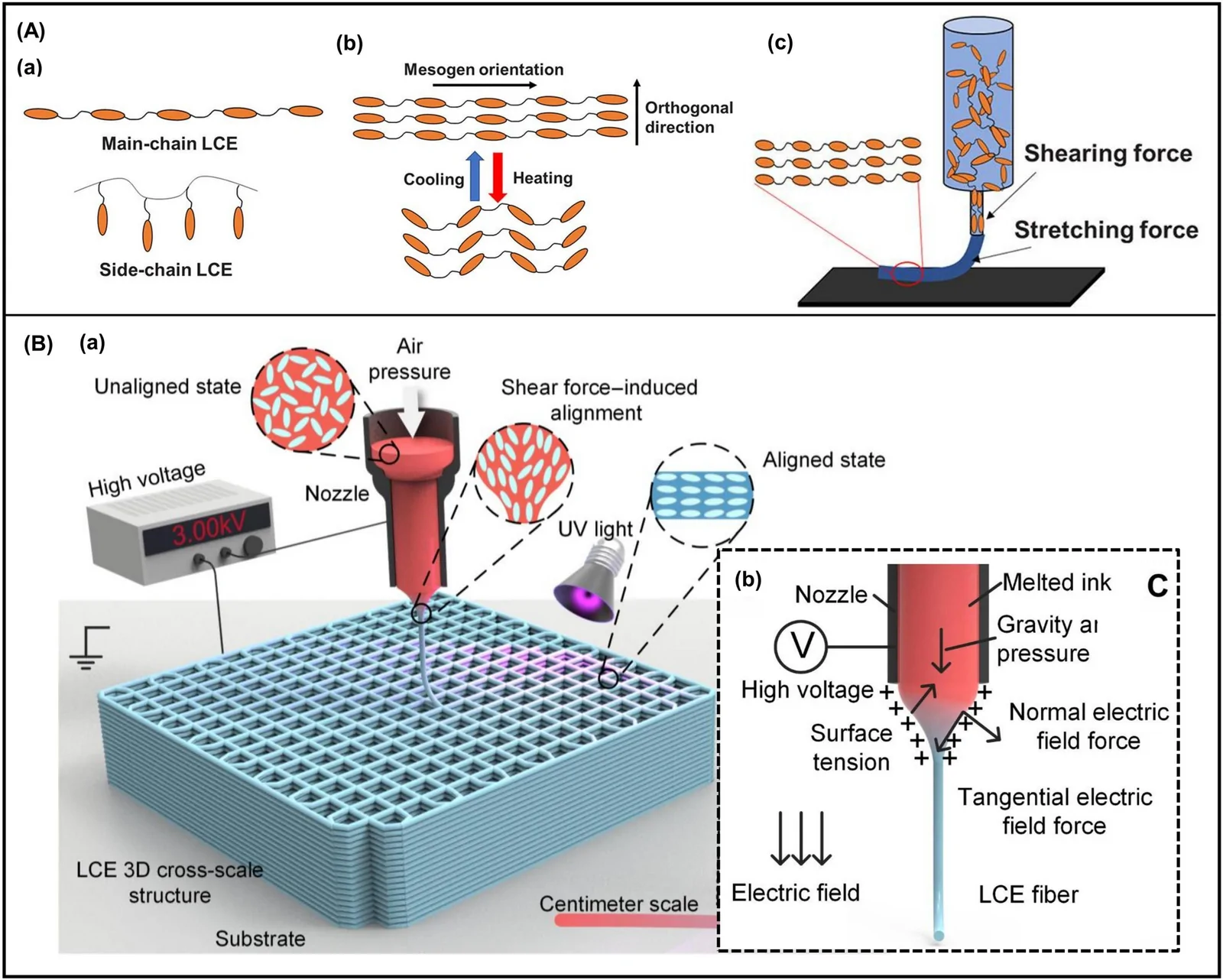
**A** (a) Structures of main-chain and side-chain LCEs, (b) Schematic of reversible thermal contraction of LCE chains, (c) Extrusion-induced mesogen alignment in the printing direction. Reproduced with permission [11], Copyright 2025, ACS; **B** Schematic of (a) MEW printing of LCE microstructure, (b) Shear forces generated in the Taylor cone during MEW. Reproduced with permission [8], Copyright 2024, AAAS

Liquid crystal elastomers (LCEs) are property-programmable soft polymers composed of flexible polymer backbones and rigid mesogenic units. These mesogens may be covalently incorporated into the main chain or tethered as pendant side groups (Fig. 4A-a) [126]. Depending on mesogen alignment, LCEs can exhibit nematic, smectic, or cholesteric mesophases. Thermotropic LCEs undergo reversible phase transitions above *T*_NI_ (temperature at nematic-to-isotropic phase transition), inducing macroscopic deformation through mesogen disorientation, wherein the polymer contracts along the mesogenic orientation and expands orthogonally (Fig. 4A-b) [127]. Specifically, monodomain LCEs, characterized by uniaxially aligned mesogens, can exhibit reversible actuation strains up to 400% [128]. Mesogen monodomain alignment, programmable during extrusion processing (Fig. 4A-c), is critical for generating directional, stimulus-responsive actuation [129, 130].

During MEW, LCE inks undergo in situ mesogen alignment primarily driven by shear forces within the nozzle and elongation forces between nozzle and collector (Fig. 4B) [8, 131]. When processed below T_NI_, mesogens are preferentially aligned along the fiber axis and are subsequently fixed in place via photopolymerization [132]. MEW enables precise control over the deposition of LCE microfibers, thereby allowing for spatially defined mesogen orientation within the *x*–*y* plane. This advantageous supports the fabrication of complex, microstructured scaffolds that exhibit programmable and reversible shape transformations. Temperature control is essential in MEW of LCEs to preserve mesogen alignment during extrusion. Effective mesogen orientation in MEW requires processing below T_NI_; however, such low temperatures increase ink viscosity and restrict material processability. A higher printing temperature can lower polymer viscosity and enable smooth extrusion but compromise mesogen alignment and performance, highlighting the need for low-T_NI_ LCEs. Recent molecular design strategies for LCEs have improved their processability while preserving mesogenic order [133]. In addition, printing parameters particularly collector speed and melt temperature, strongly affect mesogen orientation and anisotropic deformation, wherein mesogen orientation decreases with higher temperature but improves with faster speeds [134, 135]. In one representative study, LCE inks containing a photoinitiator (Irgacure 369) printed at 60 °C and UV cross-linked yielded uniform fibers (nm-tens of μm) with preserved axial mesogen alignment and reproducible thermal actuation [131]. MEW LCE microactuators (fiber diameter 4.5–60 μm) showed actuation strains of 10%–55% and stresses up to 0.6 MPa, with performance critically dependent on MEW printing conditions [8].

Beyond contraction of straight uniaxial fibers, MEW-printed LCE architectures demonstrate complex motions such as rotations and localized morphing, broadening their biomedical potential. While current researches emphasize soft robotics and remote actuators, MEW-printed LCE scaffolds with multiscale biomimetic architectures also show promise as dynamic mechanobiological platforms capable of replicating physiological strain environments for tissue modeling and regeneration. When manufactured with appropriate LCEs, such scaffolds could deliver physiologically relevant cyclic strains to embedded cells, mimicking native tissues such as the heart. The high resolution of MEW (0.1–10 μm fibers) further supports rapid actuation and short recovery times.

Although still emerging, MEW 4D printing of LCEs integrates intrinsic stimuli responsiveness of LCEs, spatial programmability of mesogen orientation, and rational 3D structural design, which holds advantages for fabricating dynamic biomimetic scaffolds. Such LCE-based 4D scaffolds enable temperature-driven morphing and self-adaptive behavior, yet their intrinsic softness limits rigid implant applications. A central challenge for LCE-based implants is enhancing their mechanical rigidity in defined states without compromising SME performance. Achieving two-way SMEs near body temperature also remains difficult. Recent molecular engineering strategies, such as introducing dynamic disulfide bonds and tailored chain architectures, have achieved LCEs’ structural adaptability with two-way SMEs [136]. Biocompatibility and biodegradability are also critical, requiring a balance between mechanical robustness, flexibility, crystallinity, and degradation, representing a central challenge for future biomedical applications of LCEs.

#### Hydrogels

Hydrogel scaffolds are widely employed in soft tissue repair due to their biocompatibility, 3D networks, and (visco-)elastic properties resembling native tissues. Stimuli-responsive hydrogels further provide dynamic microenvironments that support cell communication, ECM-like interactions, and tissue morphogenesis [137], making them promising candidates for 4D printing in biomedical applications [138]. Among them, thermoresponsive hydrogels exploit temperature-dependent swelling–deswelling transitions, where permanent cross-links stabilize the original shape and reversible domains enable temporary configurations. Their tunable activation near physiological temperatures and rapid hydration-driven volume changes are particularly advantageous for drug delivery and tissue engineering [139].

**Fig. 5:**
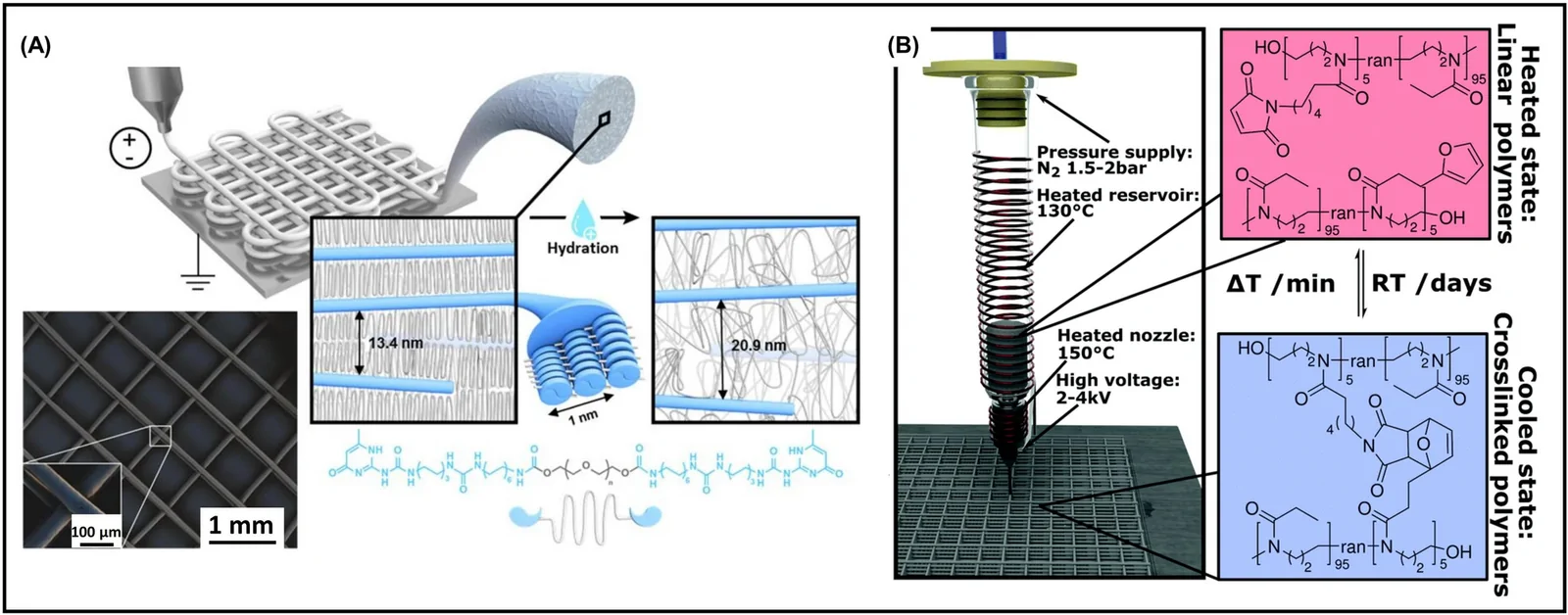
**A** Schematic and SEM image of MEW UPy-PEG10K fibrous scaffold. Reproduced with permission [140], Copyright 2020, Elsevier; **B** Schematic of MEW printing PEtOzi-based hydrogel. Reproduced with permission [141], Copyright 2020, RSC

However, direct MEW printing of hydrophilic polymers is technically challenging due to their limited electrical conductivity and hygroscopicity, which impair electrostatic jet guidance and induce discharges or repulsion. These limitations have been addressed by introducing reversible Diels–Alder (DA) cross-links post-processing. For example, ureido-pyrimidinone–PEG (UPy–PEG) supramolecular polymers were processed into hierarchical hydrogel structures (fiber diameter ~ 74 μm) (Fig. 5A) [140]. MEW-induced shear stress aligned crystalline lamellae perpendicular to fiber axes, yielding anisotropic swelling that caused rotation and buckling under high humidity (> 85%). These scaffolds preserved mesoscale architecture after swelling, and could encapsulate hydrophobic molecules, showing potential in shape-shifting systems for drug delivery. Similarly, poly(2-ethyl-2-oxazine) (PEtOzi)-based hydrogels underwent spontaneous DA cross-linking after MEW processing, yielding ordered microarchitectures (500 µm fiber spacing, 10 layers) with swelling-induced expansion while preserving integrity (Fig. 5B) [141]. These scaffolds showed long-term aqueous stability (> 4 months) and mechanical properties (Young’s modulus 0.14–0.20 MPa) well suited for soft tissue engineering. Notably, they withstand repeated aspiration through a 14 G needle and cannula ejection without structural compromise, and this combination of desirable softness with structural resilience are particularly valuable for minimally invasive biomedical applications. Importantly, PEtOzi hydrogels exhibited low cytotoxicity (IC50 = 10–100 g L^−1^) [142], and DA-based hydrogels generally display good biocompatibility [143]. WST-1 assays with L929 fibroblasts confirmed high viability (> 86%) after 48 h, indicating cytocompatibility of MEW-printed PEtOzi hydrogels. The PEtOzi system also enables stiffness tailoring through modulation of swelling behavior, which can be achieved by adjusting cross-linking density via hydrolysis and modification or by tuning material hydrophilicity. Combining MEW with reversible DA cross-linking allows property adjustment without relying on potentially cytotoxic additives such as photoinitiators, while offering straightforward post-processing functionalization with fluorophores or other biomolecules. Furthermore, DA cross-linked hydrogels can be engineered for controlled degradation, with rates tunable by cross-link density and pH, thereby aligning scaffold resorption with tissue regeneration timelines.

### Stimuli-Responsive Fillers

In addition to the inherent properties of polymers, stimuli-responsive properties could also be realized by incorporating functional fillers to endow specific responsiveness. Functional inorganic or organic fillers are incorporated into polymer matrix to enhance their mechanical strength and/or shape recovery force, increase thermal conductivity and shape recovery speed, or impart additional functionalities such as remote activation (e.g., light, magnetic, or electric) and imaging capacities [144].

#### Electroactive Fillers

Incorporating electroactive fillers such as metal particles, carbon-based nanomaterials, conductive polymers, and piezoelectric particles into polymer matrices enables Joule heating for uniform temperature distribution and faster actuation compared to conventional contact heating [145]. However, directly blending with conductive fillers often introduce electrohydrodynamic (EHD) instabilities during MEW, usually resulting in printing defects, such as jet breaking and pulsing [146]. Post-processing techniques such as surface coating MEW-printed scaffolds with conductive materials provide an alternative [147], although coatings may increase electrical resistance due to filler disruption under mechanical deformation.

#### Magnetoactive Fillers

The integration of magnetoactive fillers into MEW scaffolds offers significant potential for remote actuation, real-time imaging, and multifunctional therapeutic applications. Magnetic responsiveness has been achieved using a variety of paramagnetic and ferromagnetic nanoparticles (e.g., Fe_3_O_4_, NdFeB), and superparamagnetic iron oxide nanoparticles (SPIONs) [148–150]. SPIONs are especially attractive due to their biocompatibility, high magnetic susceptibility, and modifiable surface chemistry, which enable *in vivo* tracking of implants via magnetic resonance imaging (MRI) and support theranostic applications [151].

For instance, MEW PCL scaffolds incorporating up to 0.3 wt% SPIONs maintained well-ordered microarchitectures (33 μm fiber diameter), exhibited effective MRI contrast, and remained cytocompatibility up to 0.2 wt% [152]. Surface functionalization of PCL scaffolds with silica-coated SPIONs enabled magnetic hyperthermia, antioxidant activity, antibacterial effects, and enhanced osteogenic cell responses, underscoring the potential for combined diagnostic, therapeutic, and regenerative applications [153]. Metal–organic framework (MOF) particularly Ag/AgCl-decorated Fe-based MOFs (NH_2_-MIL-88B(Fe)) (5–20 wt%) loaded MEW scaffolds (50 μm fiber diameter) further combined cytocompatibility, Ag^+^-mediated antibacterial efficacy, Fe^3+^-endowed MRI contrast, and controlled drug release attributing to the reversible expansion–contraction behavior of the MOFs [154]. Incorporating carbonyl iron (CI) particles (up to 15 wt%) into PVDF enabled stable jetting and uniform fiber formation (30–50 μm) [155]. Scaffolds even loading with 1 wt% CI, displayed magnetic responsiveness, with higher loadings exhibiting strong magnetic adhesion and following magnetic movement at higher loadings. Similarly, PCL/Fe_3_O_4_ (up to 5 wt%) scaffolds (10 μm fiber diameter, 250 µm fiber spacing) demonstrated magnetic actuation behaviors such as flapping, gripping, and alignment under external magnetic fields, with tunable actuation strength based on nanoparticle concentration [156]. Despite the wide application of iron oxide nanoparticles (IONs), their nanoscale size raises concerns regarding cytotoxicity and genotoxicity due to non-specific cellular uptake. To mitigate nanoparticle cytotoxicity, reduced graphene oxide (rGO) carriers were employed for immobilizing IONs, improving dispersion and lowering toxicity while maintaining magnetic actuation. PCL scaffold with 20 wt% rGNP@ exhibited reversible out-of-plane deformation under magnetic fields below 300 mT, with actuation achievable at as low as 100 mT in 10 wt% composites due to their enhanced elasticity [157].

#### Photothermal and Other Fillers

Photothermal fillers (carbon black, graphene, carbon nanotubes, gold nanoparticles/nanorods) enable precise, remote actuation under light stimuli [158, 159]. Recently, fluorescent nanodiamonds (FNDs) have gained attention in biomedical applications due to their unique quantum properties and proven biocompatibility. When integrated into polymer matrices, FNDs enhance hydrophilicity and thermal/chemical stability, and modulate cellular behaviors. Their nitrogen vacancy (NV) centers provide intrinsic fluorescence and quantum sensing capabilities, enabling real-time, noninvasive monitoring of local magnetic fields, temperature variations, and intracellular free radical levels. PCL scaffolds blending with 0–0.1 wt% FNDs exhibited well-defined microarchitectures (20 µm fiber diameter, up to four stacked layers) [160]. Even ultralow FND concentrations (0.001 wt%) significantly enhanced tensile modulus (1.25-fold), thermal resistance, and hydrolytic stability. The NV centers enabled T₁ relaxation behavior analogous to MRI contrast, allowing nanoscale tracking of scaffold degradation and free radical formation. Human dermal fibroblasts confirmed scaffold biocompatibility, with a twofold increase in cell proliferation compared to pristine PCL scaffolds. Incorporation of thermochromic dyes (up to 4 wt%) into PCL scaffolds yielded scaffold with ordered microfibrous architectures (20 µm fiber diameter), and enabled reversible color changes at dye-specific transition temperatures [161]. Multimaterial scaffolds combining dyes T35 and T38 in alternating orientations enabled stepwise, color-coded temperature sensing.

Collectively, functional fillers often serve as thermal converters for localized actuation and shape transformation, expanding the responsiveness, actuation kinetics, and mechanical properties of MEW scaffolds. However, challenges in filler dispersion and biocompatibility remain. Filler agglomeration, phase separation, and weak filler–matrix interfacial interactions deteriorate both printability and actuation performance. High filler loading increases melt viscosity and disrupt jet stability, often causing nozzle clogging, fiber disorder, and structural defects. The use of non-conductive, thermally stable nanofillers preserves EHD stability. While solvent-based dispersion is commonly used, residual solvents may negatively affect scaffold biocompatibility and composite material integrity. Solvent-free techniques, such as twin-screw extrusion, offer scalable processing with improved dispersion and filler–matrix interactions.

### Biomedical Application Requirements

Stimuli-responsive materials for 4D bioimplants must meet both general and application-specific requirements [99]. Beyond shape memory performance, they should provide biocompatibility, appropriate mechanical properties, favorable surface chemistry, and controllable biodegradation to support defect bridging, cell adhesion and infiltration, and seamless tissue integration without provoking immune rejection or chronic inflammation. Biocompatibility is fundamental, encompassing non-toxicity, hemocompatibility, and non-immunogenicity. Mechanical requirements involve balancing strength, stiffness, elasticity, and toughness to provide durability while avoiding adverse biomechanical effects. Controlled biodegradation is also essential, with degradation products that are non-toxic and a rate synchronized with tissue regeneration [162].

Polyhydroxyalkanoates (PHAs), a family of microbial biopolyesters with over 150 monomers [163], represent promising candidates. Produced by bacteria without additional solvents, PHAs degrade primarily via PHA depolymerases through surface erosion, enabling controllable degradation kinetics with minimal auto-hydrolysis. Their tunable crystallinity, flexibility, melting temperature, and excellent biocompatibility make them attractive thermoplastic biomaterials for MEW. PHA-based scaffolds have demonstrated long-term drug release, mild tissue response, and safe, non-genotoxic degradation products, highlighting potential in drug delivery and tissue engineering [164]. For instance, MEW of poly(3-hydroxybutyrate-co-3-hydroxyvalerate) (PHBV) and PHBV/P34HB blends produced multilayered scaffolds with diverse fiber orientations [165]. Compared with PHBV alone, the blends showed enhanced thermal stability, printability, and tunable enzymatic degradation, while supporting excellent adhesion, proliferation, and viability of human dermal fibroblasts. Unlike conventional rigid scaffolds that may damage soft tissues or require surgical removal, PHAs provide flexible, cell-supportive architectures with on-demand enzymatic degradability, making them highly attractive for biomedical applications.

In addition to stimuli-responsive polymers, nanofillers play a pivotal role in tailoring 4D scaffold performance. Incorporating functional fillers into polymer matrices imparts multifunctionality but raises safety concerns, as their nanoscale size and high surface reactivity may induce reactive oxygen species generation or toxic ion release. Filler characteristics such as size, shape, surface charge, and dispersibility critically affect cellular uptake, immune activation, and biodegradation [166]. For instance, rod-shaped IONs exhibit higher internalization and stronger pro-inflammatory responses than spherical ones, while poor dispersibility impairs biodegradability and increases cytotoxicity. Although some fillers (e.g., AuNPs) show good biocompatibility, others such as CNTs can cause severe toxicity depending on configuration and surface chemistry, underscoring the need for standardized synthesis and predictive toxicity assessment. Surface modifications, including silica (SiO_2_) coatings on Fe_3_O_4_ and ZnO nanoparticles [167], or PEGylation of CNTs [168], can improve dispersibility and reduce cytotoxicity. Optimizing filler concentration, surface chemistry, spatial distribution, and polymer compatibility is essential to balance printability, mechanical performance, and biological safety.

Finally, sterilizability is a non-negotiable requirement. Implant materials must tolerate sterilization procedures without loss of functionality, as non-sterilizable materials cannot be safely used in vivo or even in vitro.

## Actuation Methods for Smart Materials

The stimulus, acting as a trigger for dynamic and adaptive behaviors of 4D scaffolds, requires careful selection and calibration, particularly in biomedical applications where functionality, safety, responsiveness, and clinical feasibility are critical. Stimuli can be broadly categorized as physical (e.g., heat, light, electric/magnetic fields, ultrasound), chemical (e.g., solvents, humidity, pH, ions), and biological (e.g., enzymes, glucose, nucleic acids). 4D scaffolds should be rational programmed to respond to the intended stimuli based on the specific application. For instance, in tissue engineering and biomedical implants, cytocompatible stimuli are essential, excluding high temperatures and harsh chemicals, while in robotics and actuators, non-contact triggers such as light or magnetism are preferred for remote control.

Among these, heat-based activation remains the primary trigger for shape morphing in SMPs, thermoresponsive hydrogels, and LCEs due to its simplicity, predictability, and versatility. Temperature changes induce structural transformations via mechanisms such as SMEs, where materials recover their predetermined shape above T_trans_, or thermal expansion/contraction due to variations in molecular structure [171]. Contact heating (e.g., hot liquid or air) is limited in operational scope, particularly for materials with high T_trans_. Remote heating addresses this by incorporating functional fillers (e.g., CNTs, Fe_3_O_4_, Au NPs) to generate localized thermal energy through optical, electrical, or magnetic conversion. While biologically responsive materials for MEW 4D printing remain limited, this section focuses on physical (contact and remote heating) and chemical (solvents) actuation. Table 2 summarizes an overview of typical stimuli for 4D printing, including the mechanism, smart materials, advantages and limitations, as well as their biomedical applications and in vivo application constrains.

**Table 2:** Summary of actuation methods for smart materials

	Stimulus	Mechanism	Materials	Advantages	Limitations	Biomedical applications	In vivo constrains
Heat	Contact heating (hot liquid/air or body temperature)	Shape memory effect; nematic-to-isotropic transition; pase transition based on LCST (lower critical solution temperature); anisotropic thermal expansion	SMPs, LCEs, Hydrogels, Composites	Simple operation; wide material options; environmental friendly; high biocompatibility; uniform heating	Limited localized control; slow response rate; requires physical contact	Bioimplants; drug delivery; soft robots	Narrow physiological temperature window (36–38 °C); risk of hyperthermic injury
Electric field	Voltage	Joule heating	Conductive fillers incorporated into SMPs, LCEs, hydrogels	Fast response; precise and real-time control; remote actuation	Limited to conductive fillers; complex material processing; risk of electric exposure; localized overheating; requires physical contact	Muscle and neural tissue stimulation; actuators; soft robots; drug delivery	Difficult in vivo regulation; risk of interference with natural bioelectric activity
Light	(Near-) infrared	Photothermal effect	Infrared-absorbing fillers incorporated into SMPs, LCEs, hydrogels	High spatial control; non-contact actuation; fast response	Limited tissue penetration; strong material dependency	Soft robotics; tissue engineering	Inefficient in deep tissues; risk of overheating
	UV/visible	Photochemical effect	Photochemical-responsive polymers	High spatial /temporal precision; rapid and reversible response; remote control	Limited material availability; risk of photothermal damage	Photoactivated implants; drug delivery	Limited tissue penetration; risk of phototoxicity and thermal damage
Magnetic field	Alternating magnetic fields	Magnetization; induction heating	Magnetic fillers incorporated into SMPs, LCEs, hydrogels	Remote, fast, and precise control; deep tissue penetration; biocompatibility; rapid actuation	Material dependency; filler clustering; limited field source	Bioimplants; drug delivery tissue engineering; real-time imaging and monitoring; actuators; soft robotics	Uneven field distribution; particle accumulation risk
Solvent	Water	Swelling/Deswelling via asymmetric cross-linking densities	Hydrophilic polymers	Abundant in vivo; biocompatible; enables large deformations	Limited to hydrophilic materials; slow response; low spatial control	Hydration-responsive implants; tissue engineering; drug delivery	Delayed response in low-moisture tissues; risk of uncontrolled swelling
	Ions	Ion-induced swelling/deswelling	Ion cross-linked hydrogels	Simple design; reversible deformations	Limited materials; slow response; low spatial control	Targeted drug delivery; tissue engineering	Potential toxicity or immune response
	pH	Protonation and deprotonation of functional groups	Polyelectrolytes	Simple design; reversible deformations	Limited materials; slow response; low spatial control	pH-sensitive implants; drug delivery; tissue engineering	Limited to specific pH ranges

### Contact Heating

Contact heating via hot liquid baths or hot air offers a straightforward approach. Compared to water, oils with lower heat capacity and vapor pressure improve actuation efficiency by reducing energy consumption and evaporation [172]. Unlike hot liquid, hot air serves as a trigger not only for phase transitions in thermoplastics, but also for dehydrating hydrogel scaffolds, inducing volumetric changes [173].

**Fig. 6:**
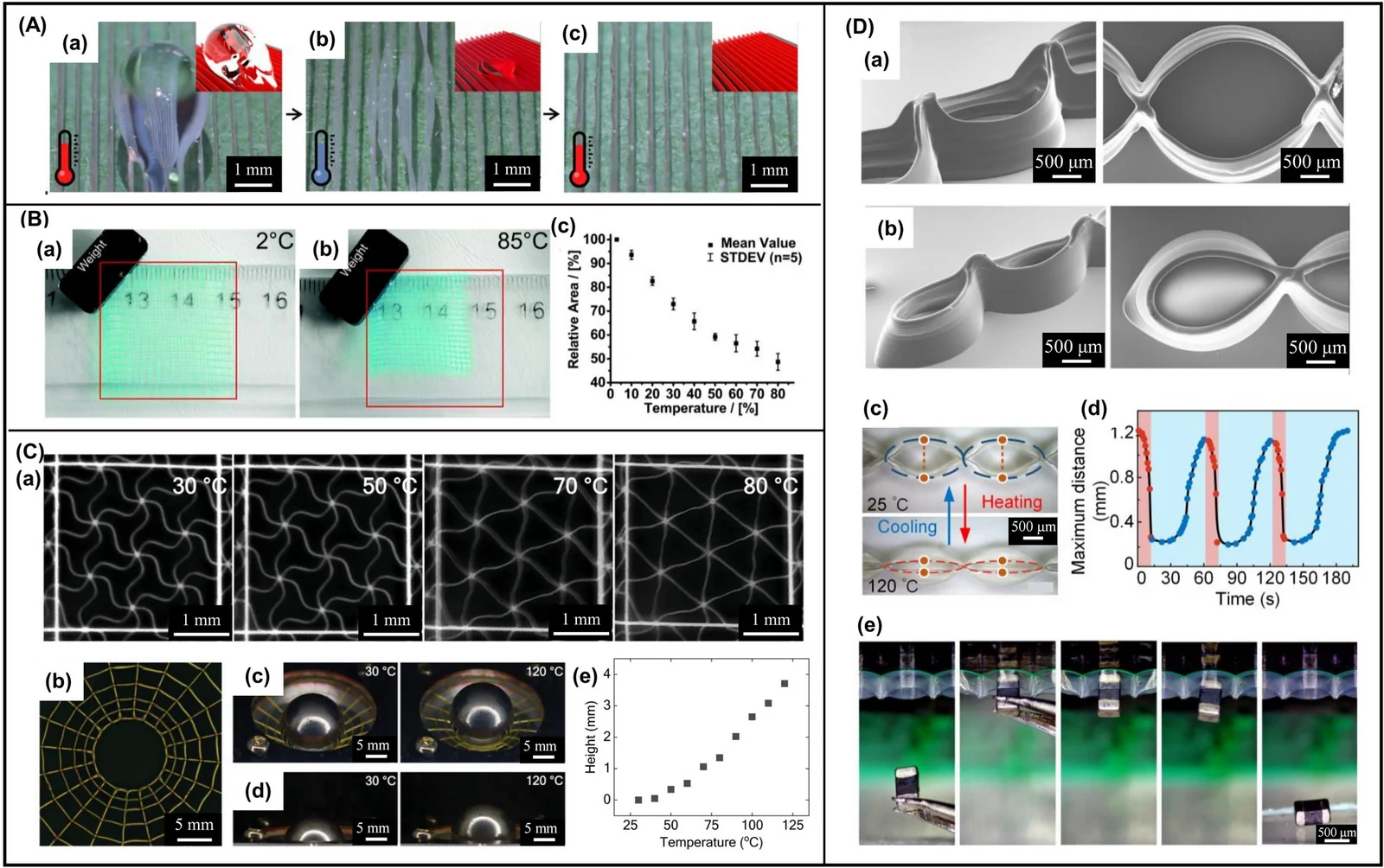
**A** Shape memory behavior of TPU PBA-75 lamellar "wall" structure, (a) water droplet-induced deformation at 70 °C, (b) fixed shape at 4 °C after droplet removal, and (c) shape recovery by reheating to 70 °C. Reproduced with permission [120], Copyright 2022, ACS; **B** Temperature-dependent hydrogel swelling visualized at (a) 2 °C and (b) 85 °C, (c) Relative scaffold area change with temperature. Reproduced with permission [141], Copyright 2020, RSC; **C** (a) Thermoactuation of hexagonal lattice LCE scaffold (fiber diameter: 25 µm, spacing: 950 µm) underwater, (b) LCE spiderweb-like scaffold. The scaffold supports a steel ball (16.26 g) at room temperature and raises it upon heating, (c) oblique view and (d) lateral view. (e) Ball height plotted against temperature. Reproduced with permission [131], Copyright 2023, Wiley; **D** SEM images of LCE microgrippers with (a) length 3 mm × width 2 mm and (b) 1.5 mm × 1 mm. (c) Photographs of thermal-responsive microgripper in open and closed states, (d) Cyclic actuation behavior over time, (e) Grasping and releasing of a 2 mm inductor by the microgripper. Reproduced with permission [8], Copyright 2024, AAAS

In SMPs, deformation fixed by cooling is restored when heating above *T*_trans_ [114]. A MEW-printed TPU PBA-75 lamellar "wall" showed reversible stiffness and deformability, enabling water droplet-triggered deformation and shape locking, with full shape recovery upon reheating (Fig. 6A) [120]. This thermally responsive behavior was further exploited to develop microfluidic valves, where droplet-induced actuation at 70 °C enabled reversible valve opening/closing. When the phase transition of SMPs is programmed at physiological temperatures, their shape-morphing properties become particularly useful for the 4D printing of deployable medical devices and implants [174].

In thermoresponsive hydrogels, temperature-dependent swelling contributes to their shape transformations. MEW-printed PEtOzi scaffolds shrink from ~ 9 to 4.8 cm^2^ (53%) when heated above physiological temperature, with swelling–deswelling occurring within seconds in a fully reversible manner [141] (Fig. 6B). The deswelling progressed steadily from 3 to 50 °C at a rate of ~ 10% area reduction per 10 °C, but slows markedly beyond 50 °C (Fig. 6B-c). Owing to the tunable lower critical solution temperature (LCST) of poly(2-oxazoline) (POx) and poly(2-oxazine) (POzi), such deswelling profiles can be tailored to meet future biomedical application needs.

In LCEs, heating above T_NI_ induces a transition from ordered to disordered mesogens, resulting in axial contraction and transverse expansion [175]. When these elastomers are locally activated, the volumetric expansion or shrinkage leads to noticeable geometric deformations. In some cases, this actuation exhibits large amplitude and reversibility. For example, MEW-printed LCE microfibers with controlled mesogen alignment exhibited up to 40% reversible contraction upon heating [134]. Sinusoidal LCE microfibers exhibited thermoactuation shape morphing under water (Fig. 6C-a). A spiderweb-like LCE structure lifted a 16.26 g steel ball, with lifting height increasing with temperature [131] (Fig. 6C-c). MEW-printed LCE microgrippers also demonstrated repeated grasped and subsequently released an inductor upon cyclic thermal actuation (Fig. 6D-e) [8].

### Electric Field

Actuation speed via hot liquid/air is often slow due to the low thermal conductivity of smart polymers. Joule heating, or resistive heating, another way of contact heating, converts electrical energy into localized heat via conductive fillers or coatings, mainly metal particles and carbon materials (e.g., graphene, CNTs, carbon fibers, carbon black) [102, 144]. This enables precise, real-time, and rapid activation, extensively explored for robotics and actuators. Despite its advantages in control and efficiency, design must consider filler geometry, resistivity, and safety to avoid overheating.

For example, patterned Au electrodes on SMPs have been used to achieve efficient Joule heating-driven actuation at a relatively low voltage of 13.4 V, significantly improving temperature distribution and shape recovery speed [176]. Importantly, the resistance evolution of Au layer enabled real-time monitoring of the shape recovery process of SMPs. In addition, graphene-integrated 3D hydrogel scaffolds have been developed as electroactive platforms, enabling pulsatile, ON/OFF drug release under low electrical voltages. Owing to the heat-dissipating properties of graphene, these hydrogels reduced resistive heating-induced tissue damage upon a voltage of 10 V, as confirmed in mouse skin implantation studies [177]. Importantly, and most critically, applying electrical stimulation near excitable tissues poses significant risks, as excessive fields may induce cardiac arrhythmias (> 3.0 V m^−1^) or disrupt ion channel dynamics in the central nervous system, leading to neuroinflammation or apoptosis [178]. High frequency (> 1 kHz) leads to nerve blocking or spike rate adaptation [179]. Therefore, the development of electroresponsive materials with safe stimulation thresholds and robust biocompatibility is essential for deploying conductive SMPs in excitable regions.

### Light

Light is an attractive stimulus in 4D printing due to its non-contact interaction, strong directionality, and ease of control [180]. It enables wireless, programmable, and localized actuation with rapid switching and remote manipulability, making it particularly advantageous for robotics and soft actuators requiring instant responsiveness [181]. The modulation of irradiation parameters (e.g., intensity, duration) allows fine control over the extent of shape transformation.

Light-responsive materials are generally categorized into photochemical and photothermal systems. Photochemical-responsive materials, such as ultraviolet (UV, < 400 nm)-responsive materials, convert light energy into mechanical motion through molecular photoreactions that alter properties like dipole moment, conformation, and cross-linking density, thereby triggering macroscopic shape changes. For example, azobenzene-functionalized LCEs produced by digital extrusion 4D printing demonstrated reversible UV/blue-light-driven deformation with actuation forces up to 24 kPa, tunable by light intensity and wavelength [182]. Photochemical-responsive hydrogels leverage molecular-level structural changes to regulate swelling, mechanical behavior, and shape deformation, offering significant potential for controlled drug delivery and tissue engineering [11].

**Fig. 7:**
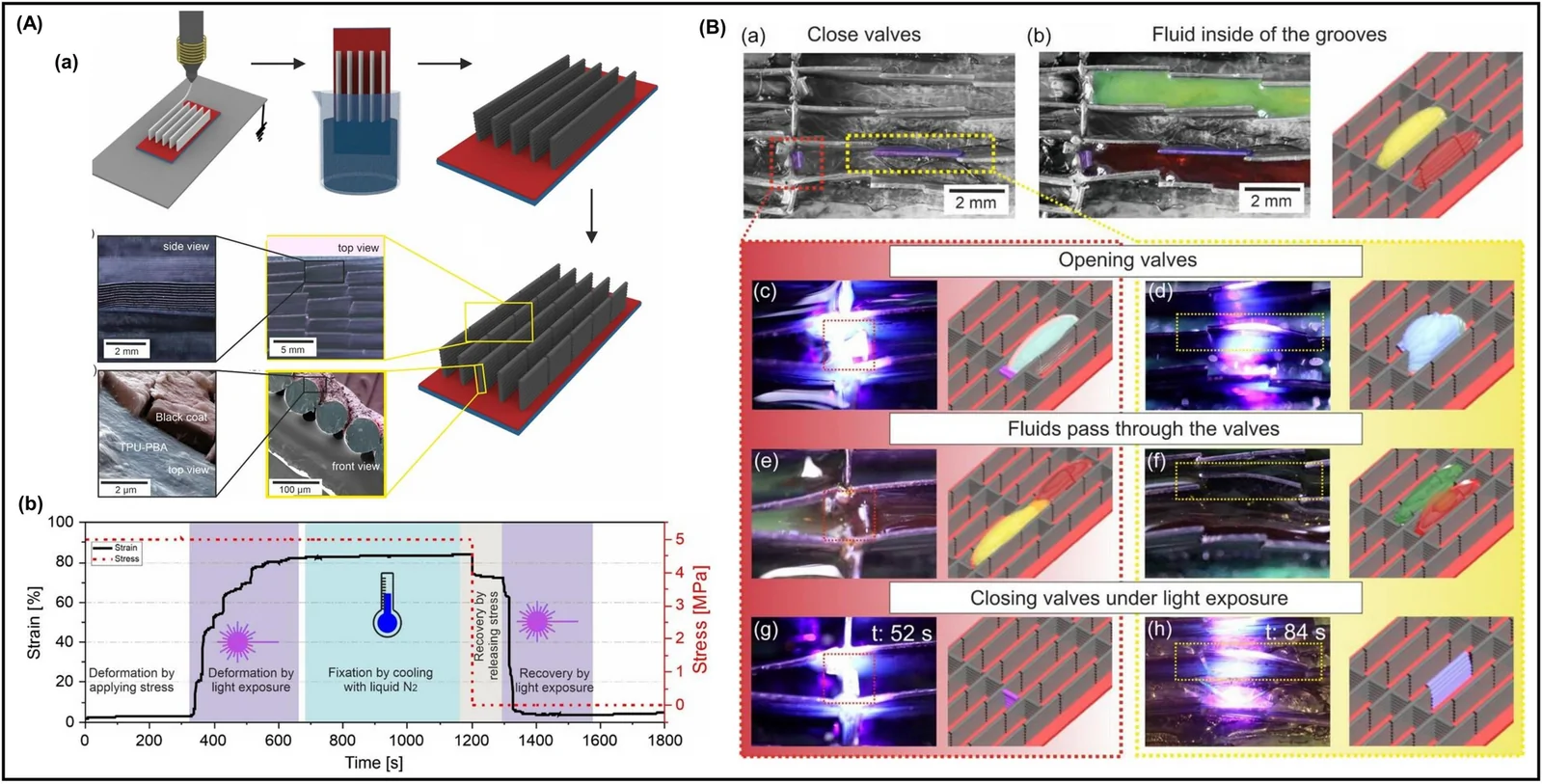
**A** (a) Scheme of fabrication of photosensitive TPU PBA lamellae via MEW, with light-absorbing coatings enabling photothermal actuation. The insert SEM images show coated surfaces (pink color: black coating, blue color: polymeric fiber). (b) Photothermal shape memory behavior measured in dynamic mechanical analyzer. **B** Application of independently light-controlled smart valves, (a, b) closed valves retain colored liquids, (c, d) advancing water volume opens valves, (e, f) fluids mixed and circulated to other grooves, (g, h) flap recovery induced by photothermal effect. Reproduced with permission [121], Copyright 2023, Wiley Online Library

Photothermal-responsive systems, in contrast, rely on fillers such as AuNPs [183] or CNTs that absorb light, typically infrared (IR)/near-infrared (NIR) (700–2500 nm), and convert it into heat to actuate the polymer matrix [184]. For example, MEW-printed TPU PBA-75 lamellae coated with a ~ 0.9 μm thick photothermal black ink layer displayed light-induced shape memory behavior, functioning as smart valves for liquid regulation [121] (Fig. 7). Copper ion-loaded polydopamine (Cu(II)@PDA) NPs were incorporated into TPU to obtained 4D composites with excellent NIR light responsiveness and antibacterial properties, which are crucial for tissue engineering applications [185]. The localized actuation can be tailored by adjusting filler concentration and dispersion [186], although excessive scattering may reduce penetration efficiency. Spatially controlled IR beams also enable localized deformation [187].

Despite these advances, several limitations persist. UV activation is hindered by shallow penetration, photodegradation, and safety concerns in biomedical applications. Visible light (400–700 nm) offers a safer alternative with greater penetration and reduced degradation, yet stimulation depth is still limited to < 1 mm due to tissue absorption and scattering [188]. Because penetration strongly depends on wavelength, NIR-responsive systems are preferred for deep tissue applications, but the material options remain limited. In addition, excessive photothermal effects risk tissue overheating and reactive oxygen species (ROS) overproduction [189, 190]. Future research should therefore prioritize innovative light delivery strategies to mitigate attenuation of shorter wavelengths, and expand the range of responsive materials for NIR stimulation, enabling deeper and safer activation. Ultimately, achieving reliable actuation of 4D-printed bioimplants requires uniform and precise irradiation, which depends on careful optimization of wavelength, intensity, exposure time, and material absorption.

### Magnetic Field

Magnetic field stimulation offers precise spatiotemporal control, remote operation, and versatility, attracting increasing attention for adaptive bioimplants and soft robotics. Magnetic responses are typically realized by embedding magnetic nanoparticles (MNPs) such as Fe_3_O_4_ or NdFeB into polymers (e.g., PCL, PLA, TPU, LCEs, and hydrogels). Magnetic actuation is generally categorized into magnetization-driven and magnetothermal-driven mechanisms.

**Fig. 8:**
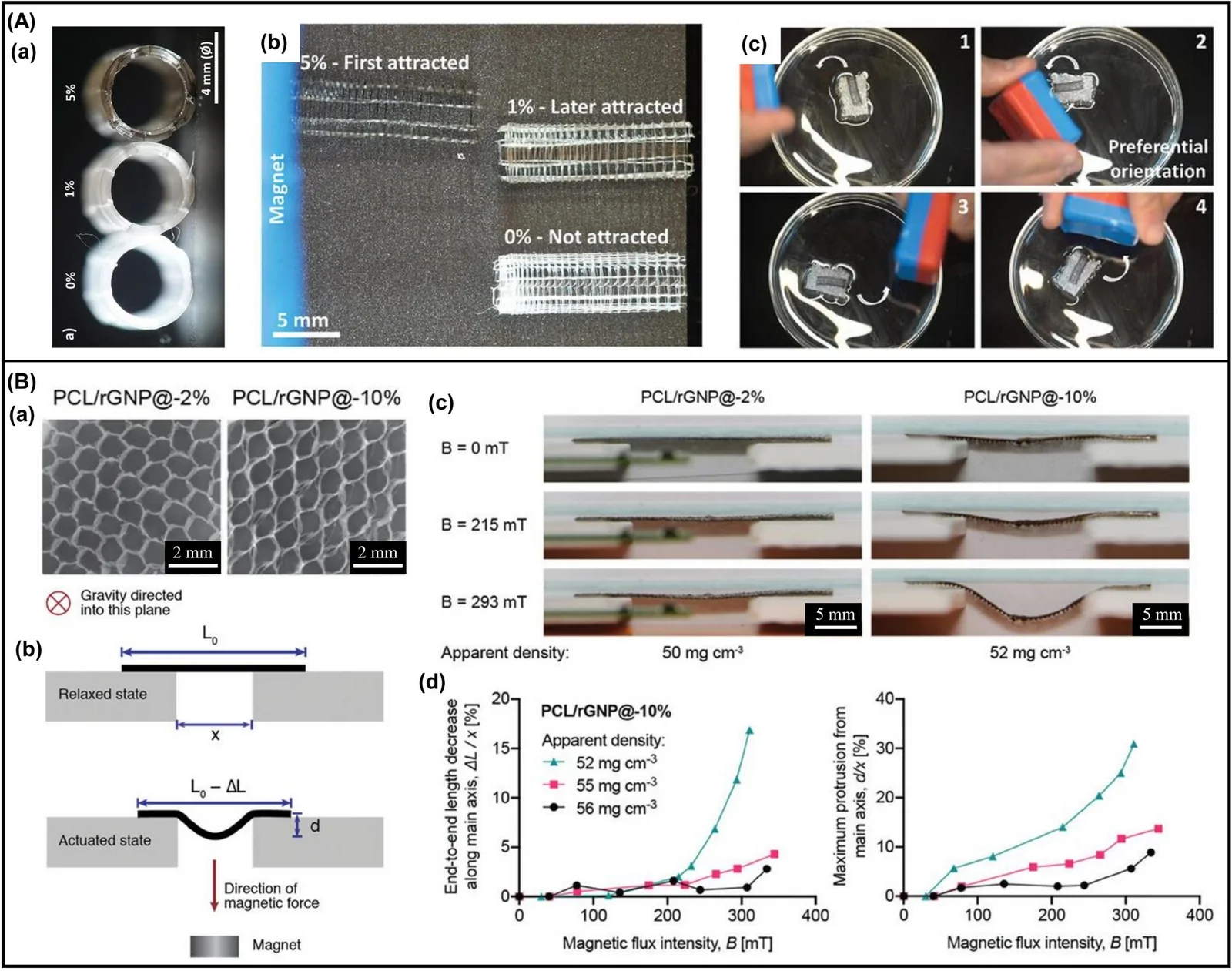
**A** Photographs of (a) MEW PCL tubes containing 0, 1, and 5 wt% of Fe_3_O_4_, (b) their different magnetic response, (c) constant rotation of 5 wt% tube aligning toward the magnet. Reproduced with permission [156], Copyright 2023, Wiley; **B** (a) SEM images of PCL/rGNP@ composite scaffolds, (b) Setup for magnetic actuation assessment, (c) Snapshots of constrained scaffold actuation under a magnetic field, (d) Magnetic flux density–deformation curves, end-to-end length contraction (left) and out-of-plane protrusion (right) for PCL/rGNP@-10% scaffolds. Reproduced with permission [157], Copyright 2024, Wiley Online Library

In magnetization-driven systems, MNPs couple with the polymer matrix, imparting anisotropic elasticity, swelling properties, and rapid responsiveness to static or alternating fields. Shape change arises from bulk magnetization as dispersed MNPs align with an external field. For example, NdFeB particles embedded in printed filaments reorient to generate permanent magnetic moments. Increasing field strength drives MNPs alignment toward saturation, while field removal induces dipole randomization, resulting in reversible shape transformations. MEW-printed PCL/Fe_3_O_4_ tubular scaffolds with 0, 1, and 5 wt% Fe_3_O_4_ displayed increasing magnetically induced movement (Fig.8A–b). The gradient tubular scaffold (fiber spacing from 850 to 250 μm in 50 μm increments) with 5 wt% Fe_3_O_4_ showed constant rotation toward the magnet from the side with smaller fiber spacing [156] (Fig. 8A–c). By precisely tuning fiber geometry and nanoparticle spatial distribution, localized responsiveness and dynamic shape changes were achieved. Similarly, MEW-printed PCL/rGNP@ scaffolds displayed magnetic actuation, with deformation proportional to magnetic flux density [157] (Fig. 8B). Although these systems enable complex, programmable morphologies, limited particle mobility within the matrix restricts large-scale deformations.

In magnetothermal-driven systems, alternating magnetic fields generate localized heating through hysteresis losses in MNPs [149]. Incorporated MNPs into SMPs, this heating elevates polymer temperature above T_trans_, thus triggering the SME [148]. Such magnetothermal actuation offers remote, non-contact heating with deep penetration and rapid response, while spatially patterning MNP distribution enables localized activation. For biological applications, magnetic parameters must be carefully controlled to avoid tissue damage [191]. Frequencies in the 50–100 kHz range are generally considered safe [10]. For example, Fe_3_O_4_/PLA composites reached 40 °C heating within 60 s under 27.5 kHz, enabling shape recovery near body temperature for potential bone repair [192]. PLA/TPU/ Fe_3_O_4_ scaffolds also demonstrated excellent fixity, recovery, and rapid actuation in complex geometries [193].

Magnetic stimulation holds strong promise for deep tissue applications, as magnetic fields are minimally absorbed or scattered by human tissue and can propagate freely in 3D space [194]. Direct depolarization of excitable tissues has been demonstrated under sufficiently strong magnetic fields in the low Tesla range, as in transcranial brain stimulation. However, the low magnetic permeability of biological tissues necessitates large field strengths, which reduce spatial resolution, increase the risk of off-target stimulation, and limit penetration depth [195]. Compared with visible and NIR electromagnetic waves that offer high spatial and temporal precision but penetrate only 1–1.5 mm [196], alternating magnetic fields (AMFs) provide greater tissue penetration. Low-frequency (< 1 kHz), high-amplitude (0.1–2 T) AMFs induce inductive coupling within superficial tissues at depths of 1–10 mm [197], whereas higher-frequency (0.1–1 MHz), lower-amplitude (1–100 mT) AMFs traverse tissue with minimal attenuation [198]. However, high-intensity AMFs carry safety concerns, with low-frequency exposure may trigger unwanted neural or muscle activation, while high-frequency fields can cause tissue heating [178]. Consequently, magnetic field intensities must remain below recommended safety thresholds [199], posing a design challenge, as inefficient receiver materials demand stronger fields to achieve effective stimulation.

### Solvents

Solvent-based stimuli (e.g., water, pH, ions) induce shape changes in 4D scaffolds primarily through hydrogel swelling and deswelling, which regulated by pore size, polymer–solvent interactions, and concentration gradients that generate internal stresses [200]. They offer accessibility without specialized equipment but lack precise spatiotemporal control.

For example, bilayer polyethylene glycol (PEG) hydrogels with distinct molecular weights exhibited differential swelling ratios, enabling spatiotemporal shape transformations into anatomically relevant geometries with ~ 90% cell viability over an 8-week culture period [201]. Similarly, mesenchymal stem cell (MSC)-laden bilayer scaffold containing hyaluronan (high-swelling) and alginate (low-swelling) layer exhibited programmable bending behaviors controlled by finely adjusting the stiffness and swelling capacity of each layer [138]. MEW-printed supramolecular hydrogel fibers exhibited anisotropic swelling (25% transverse, 4% longitudinal), enabling fiber rotation and buckling [140], while MEW-printed PEtOzi-based hydrogels achieved rapid, reversible swelling without compromising their organized microarchitecture [141]. Humidity-responsive systems rely on differential swelling of hydrophilic and hydrophobic segments to induce asymmetric deformations, where gradients in polymer composition or cross-linking density drive moisture-induced actuation and recovery upon drying.

**Fig. 9:**
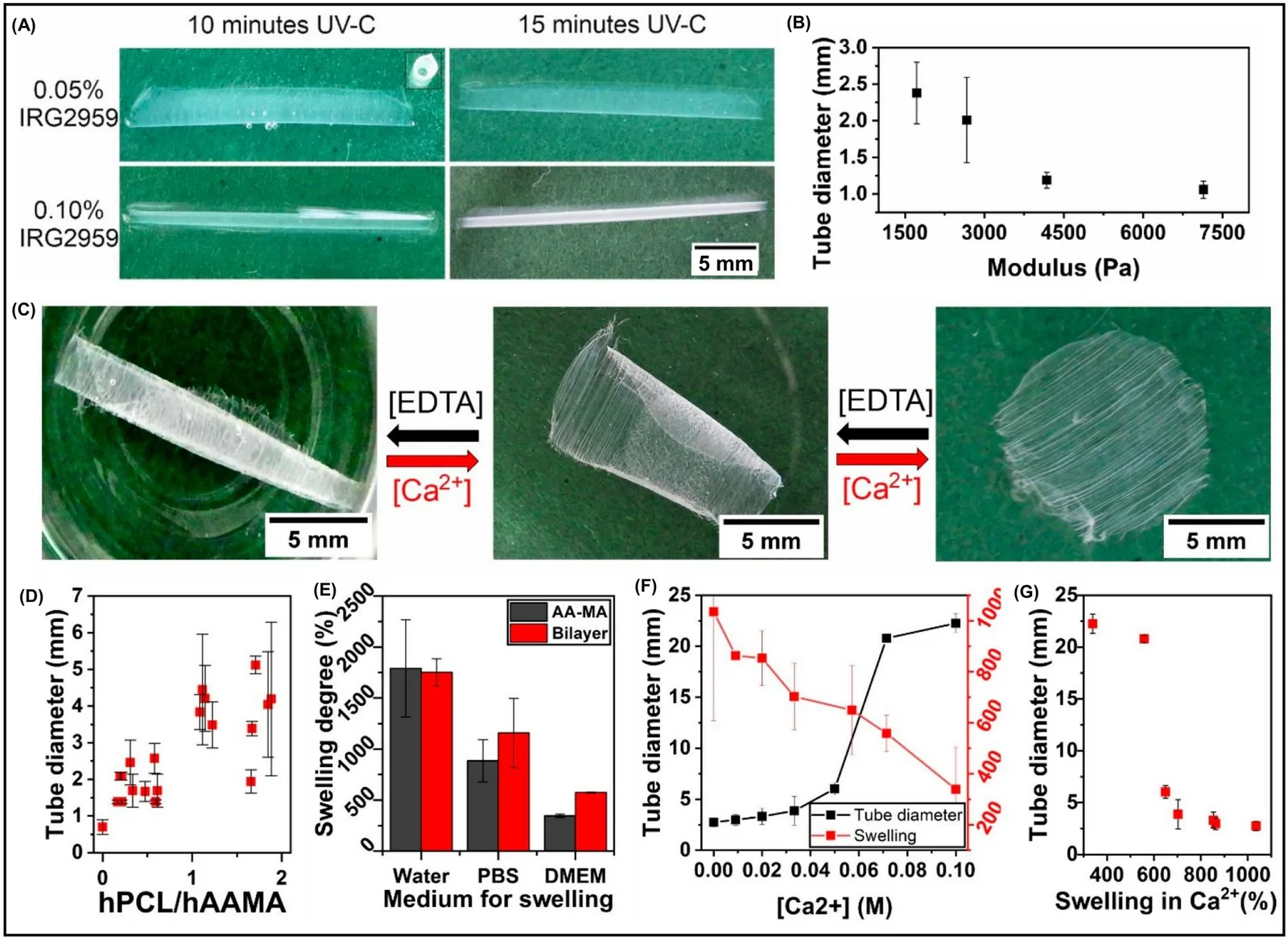
**A** Photographs of self-rolled AA-MA layer. **B** Tube diameter dependence on AA-MA hydrogel elastic modulus. **C** Reversible folding of PCL/AA-MA bilayers. **D** Influence of layer thickness on tube diameter. **E** Swelling ratio in different media. **F** Bilayer tube diameter and swelling degree as a function of calcium ion concentration. **G** Correlation between swelling and tube diameter. Reproduced with permission [169], Copyright 2021, ACS

Ion-responsive hydrogels further exploit ion cross-linking, where ionic concentration regulates stiffness, swelling, and shape-morphing behaviors [202]. For instance, a bilayer scaffold of methacrylated alginate (AA-MA) hydrogel with a top layer of uniaxially aligned MEW PCL microfibers exhibited spontaneous rolling in calcium-free media due to cross-linking-induced swelling gradients [169] (Fig. 9). The resulting tube diameter correlated inversely with elastic modulus of AA-MA hydrogel. Reversible unfolding and refolding were triggered by adding calcium salts and ethylenediaminetetraacetic acid (EDTA), respectively. Swelling was further modulated by medium ionic strength, with maximum swelling in deionized water, reduced swelling in buffer solutions due to electrostatic screening, and suppressed swelling under high Ca^2^⁺ concentrations owing to increased cross-linking density.

pH-responsive systems exploit the reversible protonation and deprotonation of functional groups (e.g., acrylic acid) to regulate stiffness and swelling for shape transformation [203]. Because of their environmental sensitivity, such hydrogels are particularly promising for drug delivery applications. However, biological pH varies across tissues and among individuals, and can shift markedly at pathological sites due to chronic inflammation. These fluctuations underscore the need for next-generation pH-responsive systems with integrated pH monitoring and adaptive response mechanisms to achieve reliable and precise performance in vivo.

### Short Summary of Stimuli

The choice of stimulus for activating shape recovery in 4D-printed bioimplants is critical. Direct heating, though widely used, is often impractical in vivo. SMPs require T_trans_ near body temperature for recovery via physiological heating, yet the fixed ~ 37 °C provides limited control over recovery kinetics and risks premature deployment. Although recent strategies have engineered materials with activation temperatures closer to physiological conditions [204, 205], low-T_trans_ SMPs generally suffer from reduced modulus and poor mechanical strength. Remote thermal actuation, such as light or magnetic induction, enables rapid and controllable responses but demands precise regulation of heating rate, penetration depth, and exposure duration. Cells exhibit some thermotolerance through heat shock protein upregulation [206], but sustained exposure above 40 °C can impair immune function [207]. While brief tolerance up to 45 °C is possible, prolonged heating risks tissue damage, making temperatures above 42 °C clinically unsuitable [208]. Confined heating approaches, such as high-intensity focused ultrasound (HIFU), can safely activate scaffolds with higher *T*_g_ (e.g., 47 °C) while maintaining surrounding tissue within safe temperature limits [209].

## Design Strategies for MEW 4D Scaffolds

Design strategies encompass not only material selection but also structural configuration, governing the functional properties and the time-dependent shape-morphing behavior of 4D-printed scaffolds. Smart materials impart adjustable deformation, compliance, and adaptability, which have been extensively discussed in Sect. 3. Structural design, including geometry, material distribution, and internal architecture, is also crucial. Stress/strain mismatches, arising from differences in stress distributions between different materials or regions within printed scaffolds, represent a fundamental programming strategy for predictable deformation. Single-material 4D printing offers design simplicity and superior repeatability by avoiding interfacial compatibility issues but is generally limited to simple deformations. Multimaterial 4D printing integrates heterogeneous material properties within a single construct, significantly expanding the design space for bioinspired and multifunctional systems, but presents challenges such as cross-contamination between materials, material compatibility, and interface reliability. This section emphasizes structural design strategies, focusing on single-material stress/strain encoding, microscale alignment, and multimaterial assembly for advanced 4D scaffolds. The modeling and optimizing of scaffolds in 4D printing design are also highlighted.

### Single-Material Programming

#### Stress/Strain Encoding

Thermomechanical programming is widely employed to generate internal stress within MEW-printed SMP scaffolds. This approach activates the SMEs through controlled heating–cooling cycles, enabling constructs to deform and subsequently recover their original shape according to the programmed memory. For example, MEW-printed TPU PBA-75 lamellar “walls” exhibited hot water-induced deformation that was fixed upon cooling, followed by complete recovery when reheated above T_trans_ [120]. Notably, FDM printing can program SMPs in a graded manner during fabrication [112]. Heat transfer between adjacent layers induces anisotropic tensile strain, which is locked during cooling and released upon reheating, enabling programmed deformations. The magnitude and distribution of this strain can be tuned through process parameters such as melt temperature, print speed, and scaffold geometry [210–212]. Likewise, optimization of material composition, processing conditions, and structural design allows controlled pre-strain introduction during MEW fiber deposition.

For hydrogels, stress/strain encoding commonly involves network density gradients, often achieved via gradient cross-linking. Since swelling inversely related to cross-link density, less cross-linked regions swell more, bending toward the side with denser regions [213]. Porosity gradients further influence water uptake kinetics and contribute to multimodal deformation.

#### LCE Mesogen Alignment

LCEs are lightweight, soft materials known for their remarkable shape-morphing capabilities in response to external stimuli. Achieving locally controlled mesogen alignment in LCEs, ideally in a monodomain alignment, is crucial for enhancing their stimuli-responsive behaviors, which remains a key aspect in 4D printing of LCEs. For example, shearing induction is a useful method to align LCE mesogens during extrusion printing.

For LCE-based 4D scaffolds, locally controlled mesogen alignment is essential for tailoring stimulus responses. In MEW processing, shear induction aligns mesogens along the fiber axis [131]. Control of print speed and melt temperature regulates mesogen alignment [134, 135], enabling programmable deformation patterns. MEW-printed LCE scaffolds with architectures such as rectangular lattice, reentrant honeycomb, hexachiral right-handed open structure, antichiral open structure, all exhibited strong interlayer adhesion, high fiber uniformity, and tunable thermomechanical deformation [131]. A recent study demonstrated high-resolution LCE microfibers actuating with strains up to 30% and sub-33 ms response under dynamic airflow (1–15 Hz), retaining performance over 10,000 cycles [8]. These fibers responded at temperatures as low as 30 °C due to enhanced heat transfer at microscale dimensions. MEW’s ability of precise deposition of LCE fibers with tunable diameters enables the fabrication of 3D LCE architectures across multiple scales, offering promise for mechanobiology and tissue engineering as mechanically active scaffolds delivering physiologically relevant cyclic strains to cells.

### Multimaterial Assembly

Multimaterial assembly, particularly via layering and spatial patterning, represents a fundamental strategy for programming strain/stress mismatches in 4D-printed scaffolds. By combining materials with distinct thermal expansion or shrinkage behaviors, multilayered or heterostructured architectures can be fabricated with precisely defined morphing patterns. Adjusting the recovery rates and thickness ratios of individual layers enables sequential or multistep shape transformations. Alternatively, spatial distribution of different materials within a single construct allows location-specific deformation, offering greater design flexibility and functional complexity [214].

#### Multimaterial Layering

In bilayer or multilayer constructs, deformation arises from mismatched expansion or contraction between active and passive layers under external stimuli. Active layers (e.g., hydrogels or SMPs) undergo large strains, while passive layers resist deformation, generating stress differentials that drive bending, buckling, or rolling. Selective deposition of these materials in planar and thickness directions allows tailored internal stress profiles, and once the induced mismatch exceeds a critical threshold, out-of-plane deformations occurs.

**Fig. 10:**
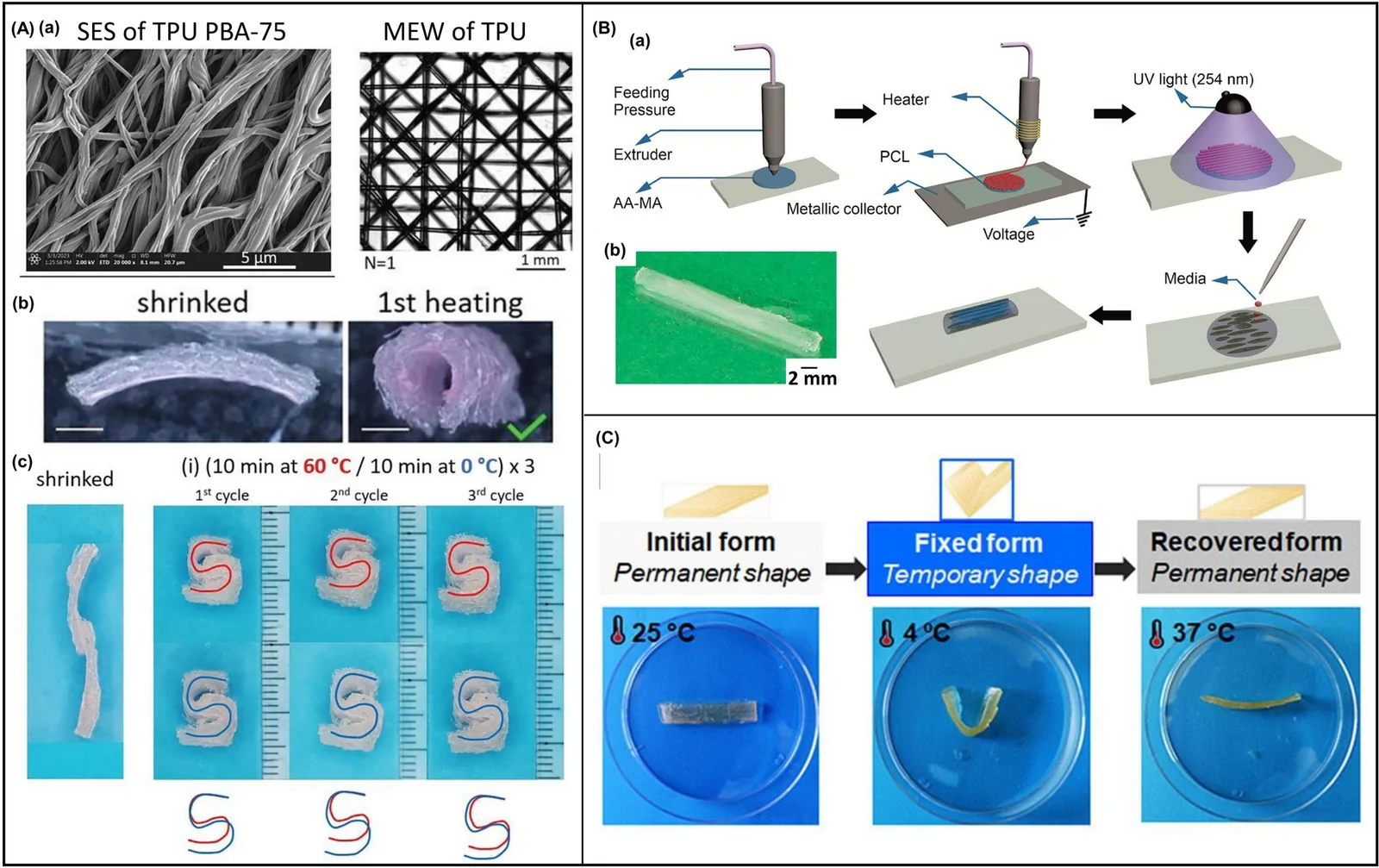
**A** (a) SEM images of electrospun fibers and MEW elastomer, (b) Specimens after shrinkage at Troom and post-heating recovery at 60 °C, (c) Two-way SME of S-shaped specimens under thermal cycles. Reproduced with permission [116], Copyright 2024, Wiley Online Library; **B** (a) Schematic of fabrication of bilayer self-folded tube via 3D printing and MEW, (b) Photography of a self-folded tube. Reproduced with permission [169], Copyright 2021, ACS; **C** Photographs showing deformation, fixation, and recovery stages. Reproduced with permission [119], Copyright 2022, ACS

For instance, highly stretched electrospun TPU nanofibers integrated with MEW elastomer matrices exhibited two-way shape memory behavior, with nanofibers reversibly contracting/elongating during heating–cooling cycles while the MEW elastomer guided overall morphing. By tuning SMP content, elastomer patterning, and bilayer thickness, reversible shape transformations were achieved [116] (Fig. 10A). In another study, a scroll-like tubular scaffold was fabricated by combining a 3D-printed AA-MA hydrogel layer with a top layer of uniaxially aligned MEW PCL microfibers [169] (Fig. 10B). These bilayer scaffolds rolled spontaneously in calcium-free aqueous media, driven by cross-linking-induced swelling gradients, and reversed upon adding calcium ions. C2C12 cells cultured on these self-folded tubular scaffolds exhibited high viability and alignment. A follow-up study HA-MA hydrogel (a component of natural ECM) with elastic PCL–PU microfibers enhanced mechanical compliance and reversibility, with folding largely independent of fiber orientation (e.g., parallel, 45°, or 90° mesh) [170]. Another system embedded MEW SMPU meshes within methacrylated hydrogels [119]. At 37 °C, composites were temporarily deformed (e.g., U or L shapes) and fixed at 4 °C (*R*_*f*_ up to ~ 90%), then rapidly recovered (*R*_r_ ~ 100%) within two minutes upon reheating (Fig. 10C). Similar results were observed in cylindrical ring structures, which fully recovered from temporary diametric deformation upon heating. The SMPU’s segmented structure enables thermally responsive phase transitions, while the hydrogel matrix supports actuation and enhances recovery dynamics. Cells seeded on the surface or encapsulated into hydrogel all showed good cell viability. More recently, combining AlgMC hydrogels with MEW PCL meshes produced vascular grafts that transformed from flat sheets into tubular geometries via anisotropic swelling and ionic cross-linking. PCL reinforcement improved mechanical integrity, suturability, and perfusion without compromising morphogenesis [16]. Furthermore, integration of LCEs with SMPs offers another route to tunable actuation. LCEs provide rapid, reversible deformation but limited robustness, whereas SMPs supply stability but poor reversibility. Their combination yields composites with synergistic properties, enabling large, reversible morphing with improved stiffness [215].

#### Multimaterial Spatial Patterning

**Fig. 11:**
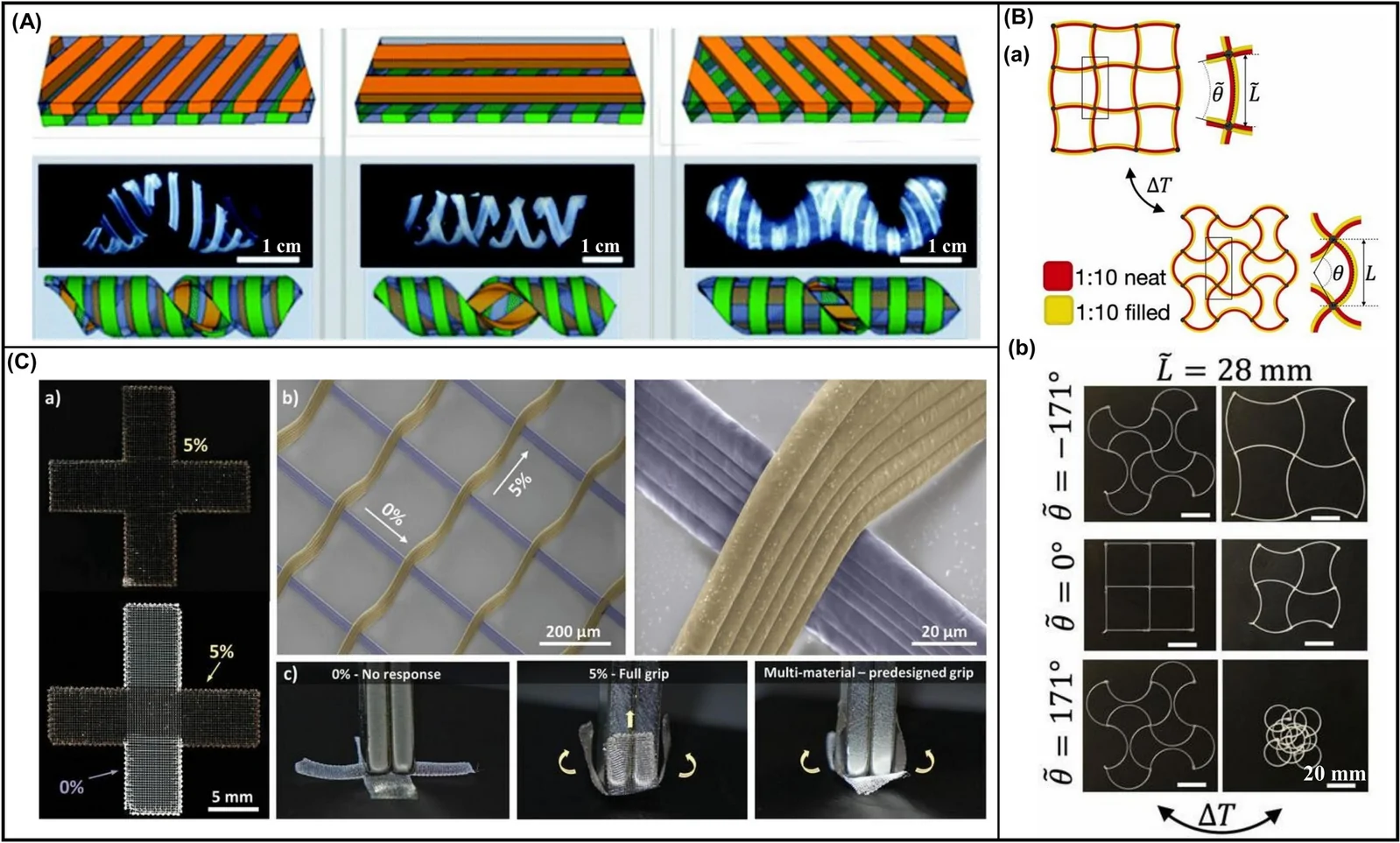
**A** Schematic of complex shape transformation by spatial patterning. Composite hydrogels with distinct responsive polymers induce right-handed helix formation at pH 9 via PAA layer swelling. Reproduced with permission [216], Copyright 2016, RSC; **B** (a) Schematic of bilayer lattice design, (b) Images of 2D lattices before and after thermal exposure. Reproduced with permission [219], Copyright 2019, National Academy of Sciences; **C** (a) Photographs of cross-shaped samples with a 0°/90° microstructure (20 mm length, 5 mm width arms, 12 layers) fabricated with 5% Fe_3_O_4_ and multimaterial PCL + 5% Fe_3_O_4_, (b) False-colored SEM images of multimaterial fiber intersections, (c) Photographs of samples deflected under a magnetic field. Reproduced with permission [156], Copyright 2023, Wiley

In contrast to layering in the vertical direction, multimaterial spatial patterning distributes active and passive materials in both in-plane and out-of-plane (Fig. 11A) [216]. Precise control over material composition, geometry and spatial interactions enables complex shape morphing and site-specific actuation [217, 218]. For example, heterogeneous lattice architectures printed with elastomeric inks of varied cross-linking density and anisotropic fillers allowed programmable shape morphing. When deposited in a curved bilayer lattice, these structures provided local modulation of expansion metrics and directional actuation, with individually programmed lattice cells achieving complex and predictable 3D transformations upon heating (Fig. 11B) [219].

In MEW-based systems, actuation can be tuned by adjusting filler type, concentration, fiber diameter and spatial placement. Smaller-diameter fibers enhance flexibility, lowering actuation force while retaining mechanical response. For instance, PCL composites with Fe_3_O_4_ fillers yielded magnetically responsive scaffolds, where actuation behavior depended on filler content, multimaterial patterning, and scaffold geometry [156] (Fig. 11C). Skeletal muscle-inspired hexagonal scaffolds with zonal magnetoactive material distribution exhibited reversible out-of-plane deformation under magnetic fields below 300 mT [157]. Moreover, MEW of microfibrous networks onto soft elastomers produced miniaturized actuators (10–15 mm length, 1 mm inner diameter) capable of full-range motion within ~ 20 ms using minimal fluid volumes [220].

### Modeling and Optimizing of 4D Scaffolds

Finite element analysis (FEA) is widely employed to stimulate the shape-morphing behavior of 4D-printed SMPs [112, 221], supporting both forward prediction and reverse design [100]. For instance, Wang et.al integrated a constitutive model with FEA to accurately simulate the deformation of FDM-printed PLA scaffolds [221]. Classical Timoshenko beam theory is often applied to analyze bilayer bending by relating curvature to strain differentials, but its small-strain assumption limits accuracy for highly responsive materials [222]. Modified analytical models have been developed for swelling-induced curvature in hydrogel bilayers [223].

A key design consideration for 4D bioimplants is ensuring mechanical properties that support complete shape recovery while minimizing mismatch with surrounding soft tissues. Computational optimization of scaffold architecture and mechanics via FEA offers an effective approach to enhance tissue compatibility, thereby reducing implantation trauma and improving therapeutic outcomes. For example, Li et al. developed a Haversian system-inspired gradient scaffold based on triply periodic minimal surfaces (TPMS) and used FEA to simulate stress conduction during implantation [224]. The simulations predicted detailed strain fields under static and physiological loading, demonstrating that TPMS-based scaffolds optimized stress conduction and reduced stress shielding by redistributing loads from the implant–bone interface into surrounding tissue. Additionally, FAE COMSOL Multiphysics simulation enable detailed predictions of stress–strain distributions and recovery behavior of 4D-printed SMP scaffolds under external stimuli [204]. ABAQUS simulations incorporating a generalized Maxwell model, WLF time–temperature superposition, and Prony series parameters successfully reproduced the recovery of 4D-printed PCL/Fe₃O₄ stents at a 30° bending angle [108]. The simulated stress distributions within the stent and its recovery behavior closely matched experimental results, underscoring the utility of FEM for optimizing SMP-based scaffolds and guiding the rational design of patient-specific biomedical implants.

Weak interlayer adhesion is a common limitation of 4D-printed SMPs, often leading to delamination and compromised shape memory performance. Conventional thermoplastics (e.g., TPU, PCL, PLA, PC, PA) rely primarily on van der Waals forces between layers, resulting in insufficient bonding. To address this, recent studies have introduced covalent or dynamic bonding strategies to strengthen interfacial adhesion. For example, Cheng et al. synthesized unsaturated PLA–PCL copolymers (U-PLA–PCL), where UV-induced cross-linking during FDM formed covalent interlayer bonds, significantly improving adhesion and enabling stable 3D constructs with reliable SMEs [225]. Additionally, dynamic bond (DB)-reinforced poly(urethane-urea-amide) (PUUA-DB) elastomer-based scaffolds incorporated dynamic covalent networks and hierarchical hydrogen bonds, exhibiting strong mechanical performance and reversible SMEs [226]. In another case, PEtOzi-based hydrogels underwent spontaneous DA cross-linking after MEW, achieving rapid, reversible swelling without compromising their organized microarchitecture. This intrinsic covalent fiber fusion further improved interlayer adhesion in printed constructs [141].

## 4D Scaffolds for Biomedical Applications

4D printing holds broad application prospects in biomedical engineering [13], with significant progress in tissue engineering, implantable devices, and drug delivery [10, 11, 227, 228]. Compared with static 3D scaffolds, 4D scaffolds can be delivered via minimally invasive procedures, conform to complex or fragile tissues [229], and provide dynamic, stimuli-responsive microenvironments that more closely replicate native conditions [178, 230]. SMP-based biomedical implants offer opportunities for personalized customization, minimally invasive delivery, on-demand actuation, and autonomous adaptation [12]. In addition, 4D printing with stimuli-responsive materials enables programmable, site-specific drug release in response to disease-associated or environmental cues [231].

### Tissue Engineering

Tissue engineering aims to develop biomimetic functional substitutes for the repair or replace of damaged/diseased tissues, yet replicating the intricacy of native structures remains a major challenge. Conventional static scaffolds support cell adhesion, proliferation, and differentiation but lack dynamic stimulation, often resulting in limited cell maturation and suboptimal tissue functionality. By contrast, 4D scaffolds can transform from planar constructs into anatomically relevant curved or tubular geometries, conform to irregular defects, and provide stimuli-responsive environments that facilitate cellular recruitment, enhance host integration, and promote functional tissue regeneration [232]. For example, SMPU scaffolds (*T*_g_ ~ 32 °C) enabled tunable cell–material interactions. When cells were seeded on scaffolds in their temporary shape and subsequently subjected to shape recovery, significant elongation of both the cells and their nuclei was observed. Remarkably, a single mechanical actuation was sufficient to trigger these morphological changes in adherent cells, highlighting the potential of these 4D scaffolds as dynamic, instructive platforms for guiding cell fate and tissue organization [233]. To address the limitations of static MEW scaffolds, magnetoactive scaffolds incorporating carbonyl iron (CI) [155] or Fe_3_O_4_ [156] particles were developed. These 4D scaffolds demonstrated fiber displacement at both micro- and macroscales under magnetic fields, and confirmed excellent cell viability. More recently, cell-laden hydrogels incorporating magnetic microparticles enabled reversible, on-demand folding, mimicking tissue morphogenesis in mucosal tissues [19]. In this section, we highlighted applications of 4D scaffolds in muscular, vascular, osteochondral tissue engineering.

#### Muscular

Skeletal muscle consists of highly organized bundles enveloped by epimysium, and this hierarchical 3D architecture determines their mechanical properties and functionality. Traditional strategies primarily induce 2D cell alignment, making it difficult to recapitulate the full complexity of muscle tissue. 4D printing addresses this limitation by facilitating dynamic transformation from 2 to 3D constructs that better mimic native organization.

For example, MEW-based magnetic composites (e.g., PCL/rGNP@ scaffolds with hexagonal pores embedded within collagen/Matrigel) guided C2C12 myoblast alignment and fusion into organized myotube networks [157]. Under cyclic magnetic field stimulation (> 100 mT), scaffolds exhibited reversible out-of-plane bending without compromising scaffold integrity and cell viability. Although distinct from the in-plane contractions of native skeletal muscle, such magnetomechanical cues support myogenic maturation. MEW further allows spatially patterned magnetically active regions to replicate region-specific muscle functions. In another study, bilayer scaffolds combining AA-MA hydrogel layer with uniaxially aligned MEW PCL microfibers exhibited spontaneous rolling in calcium-free media [169]. The transformation was reversible and tunable by factors such as the environment media, the concentration of calcium ions and the structure of the scaffold. MEW PCL fibers (∼14 μm diameter) exhibited a storage modulus (~ 8.4 MPa at 37 °C) similar to that of native collagen fibrils (~ 1.1 MPa), ensuring mechanical compatibility. These bilayer scaffolds supported myoblast viability (> 80%), increased metabolic activity, and the anisotropic inner topography-induced cell alignment. Cells were seeded on flat bilayers, ensuring homogeneous cell distribution in the folded 3D structure. A subsequent design combing HA–MA hydrogel with MEW-printed PCL–PU microfibers (100 μm spacing) yielded compliant, scroll-like bilayer structures capable of reversible deformation [170]. The PCL–PU fibers exhibited tunable elastic modulus (3–15 MPa) depending on PU content, up to 200% elongation, and partial shape recovery, thereby imparting shape-morphing functionality to the fiber/hydrogel composites. Myoblasts cultured on these bilayers showed excellent viability and efficiently aligned along the microfibers, confirming their suitability for skeletal muscle regeneration. Notably, the shape-changing response originated from the PCL–PU fibers rather than the hydrogel, underscoring the versatility of combining MEW microfibers with hydrogels to fabricate tubular constructs with spatially patterned interiors that support both dynamic responsiveness and cell organization.

Similarly, 4D printing offers promising for cardiac tissue engineering by enabling fabrication of curved constructs with intricate surface topologies that conform to native anatomy, thereby achieving seamless integration and improving functional regeneration [234]. By combining a unique 4D self-morphing capacity with expandable microstructure, the specific design has been shown to improve both the biomechanical properties of the patches themselves and the dynamic integration of the patch with the beating heart [235]. Moreover, an elastic, biodegradable SMP-based scaffold whose shape memory originated from a microfabricated lattice architecture was developed for minimally invasive delivery of human cell-derived cardiac patches via injection [236]. Furthermore, composite films integrating MEW ultrafine PCL fibers with GelMA hydrogels were developed as conformable strain sensors [237]. With tunable elastic modulus (20–420 kPa) and Poisson’s ratio (− 0.25 to 0.52), these films closely matched soft tissue mechanics and enabled real-time monitoring of heartbeats and respiration in animal models.

#### Vascular Tissue Engineering

Vascularization remains one of the major challenges in tissue engineering, as the absence of functional hierarchical vascular networks severely limits tissue viability and integration. Conventional fabrication methods often struggle to produce hollow tubular structures with the resolution and tunable diameters required to mimic native vasculature. In this context, 4D printing offers a promising alternative by exploiting shape-morphing materials to generate self-folding constructs that resemble blood vessels and can achieve adjustable diameters [238].

Recent studies have shown that shape-morphing hydrogels, such as AA-MA and HA-MA, can form ultrathin films (1–16 μm) via direct ink writing (DIW) that spontaneously roll into hollow tubes (20–150 μm inner diameters) through partial drying and anisotropic swelling [239]. However, these structures remain limited to single-layer geometries and lack the multilayered complexity and functional differentiation of native vessels. MEW microfibrous scaffolds offer a viable approach for multilayer, small-diameter vascular grafts with suitable mechanical properties and hemodynamic performance [240]. For example, hierarchical living constructs mimicking the three-layer structure of arteries and veins were fabricated by integrating volumetric bioprinting with MEW meshes, exhibiting flexural, burst, and tensile strength approaching native vascular tissues [241]. More recently, a hybrid biofabrication strategy combining 4D-printed AlgMC hydrogels with MEW PCL meshes to fabricate small-diameter vascular grafts with programmable shape morphing and reinforced mechanical integrity [16]. The constructs self-transform from flat patterns into tubular geometries via anisotropic swelling and ionic cross-linking, while PCL reinforcement supports suturing and perfusion without hindering shape morphing. Post-fabrication functionalization with human blood-derived proteins enhances cell adhesion and proliferation, and co-cultures of fibroblasts, endothelial cells, and vascular smooth muscle cells form organized bilayers with reciprocal phenotype stabilization. Although this approach highlights the potential of 4D-printed hydrogel–fiber composites to achieve biologically active, structurally adaptive grafts for regenerative medicine, it remains limited in addressing key requirements such as mechanical compliance and sustained phenotypic maintenance.

Despite these advances, most current 4D-printed vasculature is limited to microtubes of predetermined sizes, falling short of replicating the anatomical complexity of native vessels. Key challenges persist, including the reliable fusion of tube walls or edges post-morphing to prevent leakage, as well as the long-term functional maturation of the printed vasculature. Moreover, reliable fusion of tube walls after morphogenesis and functional maturation including endothelialization, perfusability, and physiological responsiveness, requires further investigation.

#### Osteochondral

Bone tissue repair requires not only mechanical support but also biocompatibility and seamless integration with surrounding tissues, posing significant technical challenges. Personalized orthopedic treatments further complicate this process due to patient-specific structural and physiological differences. SMP-based 4D scaffolds offer unique advantages for bone defect repair, as they can be implanted in a temporary, minimally invasive form, and recover their intended shape in situ to conform to irregular defects [229, 242].

Bilayer constructs that integrate deformable hydrogels with SMP layers enable independent macro- and microscale shape transformations, offering great potential for minimally invasive implantation. A 4D-printed bilayer membrane that comprising an SMP layer with responsive surface microstructures and a hydrogel layer, can be delivered in a flat state, followed by transformed into a 3D structure conforming to irregular bone defects via hydrogel swelling [243]. Meanwhile, the SMP layer enabled NIR-triggered surface switching between smooth and micropillared topographies, actively regulating cell behavior. This dual responsiveness significantly enhanced osteogenesis, with in vivo studies demonstrating superior bone formation compared to static membranes. In addition, MEW-fabricated SMPU meshes embedded within hydrogels provide reliable shape memory with cytocompatibility during shape fixation and recovery cycles [119]. Magnetic functionalization, e.g., MEW PCL scaffolds post-treated with Si-SPIONs, endowed scaffolds with superparamagnetic, antioxidant, and antibacterial properties while enhancing MC3T3-E1 pre-osteoblastic cell adhesion efficiency and proliferation [153]. Moreover, magnetoelectric hydrogel scaffolds have been proposed to provide remote electrical stimulation for osteogenesis [244].

Similarly, fully replicating the structure and function of native cartilage remains difficult. While 3D printing is widely studied in cartilage repair, 4D printing remains underexplored. By enabling biomimetic scaffolds that adapt dynamically to physiological environments and patient-specific defects, 4D printing holds promise for improved cartilage integration and functional recovery. For example, hydrogels with differential swelling, such as GelMA and OMA, have been used to fabricate programmable C-shaped, spiral, and flowerlike structures mimicking cartilage geometry [245]. In a recent study, TPU-based 4D scaffolds demonstrated multidimensional morphing for minimally invasive delivery [14]. Laminated scaffolds of high-swelling (active) and low-swelling (passive) DTPUs could be fixed into temporary 1D roll-up shapes for catheterization, recover to 2D patterns at body temperature, and subsequently morph into 3D structures to fill defects and provide mechanical support.

### Biomedical Implants

Stents are vital biomedical implants used to alleviate blockages in arteries, veins, and other luminal structures. Conventional 3D-printed stents provide anatomical conformity but remain static, limiting their adaptability to dynamic physiological environments. In contrast, 4D-printed stents fabricated from stimuli-responsive, biodegradable materials can undergo programmed shape transformations in vivo, enabling minimally invasive delivery in a compact form, expansion upon activation, and eventual degradation without the need for retrieval [10, 11]. Such dynamic behavior not only reduces implantation invasiveness but also enhances patient-specific conformity. Recent advances include self-expanding and remotely controllable stents triggered by thermal, magnetic, or photothermal stimuli, demonstrated in vascular [246], tracheal [108], orbital [247], intestinal [248] stents, and cardiac occluders [249] applications.

Biodegradable SMPs such as PLA, PCL, and PU dominate the field due to catheter deliverability and thermal expandability. For instance, FDM-fabricated PLA vascular stents with auxetic geometries achieved high shape recovery efficiency (> 91%) within 5 s [246]. In addition to geometrical innovation, remote stimulation presents another avenue for minimizing invasiveness in stent deployment. PLA/Fe_3_O_4_ composite tracheal stent showed magnetically induced recovery within 40 s and a recovery rate exceeding 99% [108]. A PU-based SMP composite stent with AuNPs/nanohydroxyapatite (nHA) exhibited excellent mechanical properties, radiopacity, and bioactivity. *In vivo* implantation in a rabbit orbital model confirmed successful shape recovery under 44 °C saline stimulation, along with effective anatomical restoration and tissue integration [247]. FDM-fabricated PEG/PLA-based intestinal stents enabled shape recovery near physiological temperature (∼42 °C) [248]. One of the persistent challenges in designing temperature-responsive stents is the elevated *T*_g_ of many SMPs, which complicates activation within the human body. A vascular stent based on poly(glycerol dodecanoate) acrylate (PGDA) was engineered to exhibit a low *T*_g_ and rapid recovery (< 0.4 s) at physiological temperatures (20–37 °C), demonstrating effective performance in both in vitro and in vivo settings [250]. Blending strategies further enhance tunability. PLA/PCL blends exploit PLA as the fixed phase and PCL as the reversible phase, allowing modulation of transition temperature by varying composition. A PLA70/PCL30 stent, with ~ 90% recovery, enabled controlled deformation for tracheal implantation [251]. However, stents operating at or below body temperature risk premature recovery, complicating deployment. To overcome this, innovative approaches beyond thermal triggering are being explored. For example, shape memory hydrogels governed by phase separation rather than heat transport demonstrated recovery kinetics controlled by internal mass diffusion, offering a new paradigm for stent design [252]. Peng et al. employed FDM-based 4D printing with a biocompatible PLA/PLGA composite to fabricate shape memory BARs with self-deployability. Incorporating 6% acetyl tributyl citrate (ATBC) reduced the *T*_g_ to 43 °C, enabling adaptive deployment at 50 °C, which was successfully validated in a pig intestinal anastomosis model [162].

Stimuli-responsive materials, including SMPs, hydrogels, and shape memory alloys (SMAs), have shown significant clinical feasibility in diverse biomedical applications. For example, SMP-based embolization devices (IMPEDE-FX plug) [253], composed of bioabsorbable polyurethane that successfully delivered via catheter and subsequently expand into porous, conformable foam upon blood contact, have been successfully integrated into endovascular aneurysm repair, promoting rapid sac thrombosis and regression while preventing type II endoleaks without increasing procedural complexity. Temperature-responsive hydrogels such as PF72, composed of poloxamer 407 and hyaluronic acid, undergo sol–gel transition at body temperature to enable sustained ropivacaine release over 72 h [254], providing prolonged analgesia in orthognathic surgery as a safe and locally active alternative to systemic opioids. Additionally, nitinol-based SMA stents (e.g., Memokath™ 051) [255] employ thermal shape memory to enable minimally invasive, durable management of ureteral strictures with immediate patency and minimal complications. These findings highlight the translational potential of stimuli-responsive materials-based implants as clinically viable, adaptable, and patient-friendly solutions across interventional, surgical, and implantable therapies.

While FDM has enabled microscale stent fabrication, MEW offers superior resolution and design flexibility for biomedical implants. Conventional bioresorbable stents often require thick struts (~ 150 μm) to ensure mechanical strength, which may elevate thrombogenic risk and disturb hemodynamics. By contrast, MEW-fabricated PCL stents reinforced with 0.1% rGO achieved much thinner struts (60–80 μm) while maintaining high mechanical robustness (Young’s modulus 520 MPa; ultimate tensile strength 21 MPa). The incorporation of rGO further enhanced protein adsorption and endothelialization without increasing thrombogenicity [256]. Although it is still early in development, MEW-based 4D printing holds great potential for engineering biodegradable implants with finely tuned structural architectures, mechanical properties, and programmed shape transformations.

### Drug Delivery System

Achieving precise drug delivery to lesion sites while maintaining effective local concentrations remains a major challenge for drug delivery systems (DDSs). Stimuli-responsive DDSs offer promising solutions to overcome the limitations of conventional approaches, including low bioavailability, systemic side effects, and poor patient compliance [257, 258]. Unlike conventional diffusion-based systems that rely on slow diffusion or hydrolytic/enzymatic degradation, these systems enable site-specific deployment and controllable release profiles (e.g., pulsatile or on-demand release) in response to external stimuli (e.g., heat, electromagnetic fields, or PH) [164, 231, 259, 260]. The integration of 4D printing further enhances these advantages by enabling minimally invasive implantation, and immobilization of DDSs at target sites through shape memory effects, thereby ensuring sustained localized release without continuous external intervention [261]. Prolonged local release not only reduces dosing frequency and side effects but also improves therapeutic efficacy and patient compliance. Nanosized DDSs hold potential to increase bioavailability through targeted delivery; however, complex physiological environments often result in non-specific retention or premature release. In this regard, in situ release via 4D printing represents a promising strategy, combining drug delivery with shape morphing to generate tissue-retentive DDSs that achieve sustained release and minimally invasive administration.

By integrating shape morphing with stimuli responsiveness, 4D-printed DDSs open new avenues for dynamic, personalized, and precise therapies in regenerative medicine and controlled release [262]. However, its application remains at an early stage, particularly MEW 4D printing-based drug delivery has not yet been explored. This section highlights recent progress in MEW 3D scaffold-based DDSs, with Table 3 summarizing material composition, structure design, drug type, loading method, and therapeutic outcomes.

**Table 3:** MEW scaffold-based drug delivery systems

MEW biopolymer	Scaffold architecture	Drug type	Drug concentration	Drug loading methods	Therapeutic effects	Refs.
PCL/PEG (90:10, 75:25)	Crosshatch meshes with pore size 1 mm or 1.5 mm, 10 stacked layers	Azithromycin	1 mg/mL	Immersion coating	Suppress the growth of S. aureus bacteria	[264]
PCL	Wavy fiber patterns with pore size 1250 µm	Vancomycin	5, 10, and 25%	Immersion coating	Suppress the growth of S. aureus bacteria	[265]
PCL	Crosshatch meshes with fiber diameter 319 µm and pore size 1.5 mm	Gentamicin sulfate, tetracycline hydrochloride	1% w/w aqueous solution	Iimmersion coating	Inhibit the P. aeruginosa strain biofilm formation	[266]
PCL	Crosshatch meshes	Milk proteins	0.5%	Pre-blending	Display low degradation, and rapid protein release, increase cell growth and infiltration	[267]
PCL:PEG (95:0, 90:5, 85:10, 80:15)	Crosshatch scaffolds with fiber diameter 8 μm, pore size 100 μm, and 15 stacked layers	Roxithromycin (ROX)	5%	Pre-blending	Improve the hydrophilicity, display effective antibacterial activities	[268]
PCL	A composite scaffold composed of ROX-encapsulated MEW microfiber layer and HAP-loaded electrospun layer	ROX, hydroxyapatite (HAP)	2% ROX, 10% and 20% HAP	Pre-blending	Promote osteogenesis, improved the level of calvaria defect repair in rats	[269]
PCL:PEG (90:10)	Scaffolds with square, triangle or rhombus pores	Ciprofloxacin (Cip)	5%	Pre-blending	Exhibited antibacterial activities	[270]
PCL	Crosshatch scaffolds with diameter 20–30 μm, thickness 2.51 mm	Fosmidomycin derivative CC366	5, 10%	Pre-blending	Prevent the formation of A. baumannnii biofilms	[271]
MeOziPentOx	Crosshatch scaffolds with diameter of 25–50 μm, and 15 stacked layers	Indomethacin (IND)	33, 50, and 66%	Amorphous solid dispersion	Water-soluble MEW fibers allow for rapid sublingual drug delivery	[263]
PCL	Melt electrospun nanofibers with diameters of ∼800 nm were deposited on the MEW mesh with fiber diameter ∼10 μm, pore size 0.2 mm, and 20 stacked layers	Paclitaxel (PTX)	2%	Pre-blended within electrospun	The solvent vapor annealing treatment of PCL nanofibers result in a nonlinear drug release profile	[272]
PCL	Crosshatch scaffold with fiber diameter 78.74 µm, pore size 896.67 µm, and the height 3 mm	bFGF, VEGF	bFGF 50 ng/µL and VEGF 16 ng/µL	Encapsulated into gelatin microcapsules	Enhance the wound healing process	[273]
PCL	Hexagonal meshes with pore side 400 μm, and 35 stacked layers	Extracellular vesicle (EV) derived from hiPSC	–	Encapsulated into GelMA hydrogels	Promote endothelial cell migration	[274]
PCL	Crosshatch scaffold with pore size 300–400 µm, and 50 stacked layers	Exosomes	–	Encapsulated into GelMA hydrogels	Improved collagen deposition and angiogenesis in vivo	[275]
PCL	Crosshatch scaffold	β-cells secreted insulin	–	β-cells encapsulated in alginate microcapsules	Normalize blood glucose in diabetic mice for at least two months without taking antirejection drugs	[276]

MEW scaffolds have emerged as versatile platforms for drug delivery, incorporating therapeutic agents via immersion coating or polymer blending. Their fibrous architectures are particularly suited for loading bioactive carriers such as hydrogel microcapsules or MOFs [154]. MEW of water-soluble polymers has further expanded design possibilities [263]. Antibiotics have been widely incorporated into MEW scaffolds. For instance, PCL/PEG crosshatch scaffolds loaded with azithromycin inhibited S. aureus, with smaller pores exhibiting faster release [264], while vancomycin-loaded wavy PCL fibers achieved 18% loading efficiency and provided burst release followed by sustained delivery over 14 days [265]. Gentamicin sulfate and tetracycline hydrochloride in PCL meshes also inhibited P. aeruginosa biofilms [266]. Pre-blending drugs into base polymers offers another route, though high drug content may compromise viscosity, stability, and printability. Examples include PCL blended with milk proteins, yielding low degradation, sustained release, and enhanced dermal regeneration [267]; PCL/PEG@roxithromycin (ROX) composites with burst-sustained release against S. aureus [268]. A hybrid scaffold combing MEW PCL@ROX microfibers with hydroxyapatite (HAP)@PCL electrospun nanofibers allowed tunable release rates, with ROX release rate inversely proportional to the HAP content [269]. Drug kinetics can also be modulated by material composition and scaffold geometries (square, triangular, rhombic pores), as seen in PEG/PCL@ciprofloxacin (Cip) scaffolds, where higher PEG increased hydrophilicity and burst release with smaller fibers amplified this effect, and pore geometry altered diffusion profiles. PEG incorporation further shifted release from degradation- to diffusion-controlled, while all Cip-loaded constructs retained antibacterial efficacy with varying degrees [270]. In addition, fosmidomycin derivative CC366 in PCL scaffolds displayed a rapid initial release for infection prevention [271]. Solvent-free incorporation of indomethacin into poly(2-oxazoline)-based triblock copolymer yielded MEW scaffolds with drug release dependent on polymer/drug ratios [263]. PCL@Paclitaxel electrospun nanofibers integrated onto MEW PCL scaffolds showed a nonlinear release rates due to opposing effects of solvent vapor annealing on crystallinity and surface area [272].

To preserve labile biomolecules, hydrogel microcapsules are often employed for encapsulation carriers. The customizable pore architecture of MEW constructs allows for geometric confinement and spatial localization of drug-laden hydrogels, enabling sequential or staged delivery of multiple agents. For instance, MEW meshes loaded with gelatin microcapsules containing different growth factors and bioactive glass (BG) particles showed staged release. BG accelerated release of basic fibroblast growth factor (bFGF), while vascular endothelial growth factor (VEGF) was released more gradually, promoting both early fibroblast migration and later-stage vascularization [273]. Similarly, extracellular vesicles (EVs) [274] or exosomes [275] embedded in GelMA hydrogels reinforced with MEW PCL meshes enabled sustained release, enhancing wound healing and neovascularization. Moreover, β-cell-laden alginate microcapsules within MEW PCL scaffolds promoted vascularization and restored normoglycemia in diabetic mice for two months without immunosuppression [276]. These developments underscore MEW’s potential for localized, sustained drug delivery, while future research should focus on integrating stimuli-responsive carriers with MEW 4D-printed scaffolds to achieve spatiotemporal release triggered by physiological cues.

## Summary and Perspectives

MEW presents a promising platform for fabricating fibrous scaffolds with micro- to nanoscale resolutions, closely resembling the structural dimensions of native ECM fibers and living cells. When combined with stimuli-responsive polymers and/or functional fillers, MEW-based 4D printing enables fabricate dynamic scaffolds with programmable shape transformations, opening new opportunities for biomedical applications. This review systematically analyzes the structural complexities achievable with MEW, from 1D to 3D constructs, and highlights advanced strategies to enhance scaffold quality. Stimuli-responsive materials compatible with MEW, including SMPs, LCEs, hydrogels, and functional fillers, are discussed, alongside the stimuli that actuate shape changes. Design strategies including single-material stress encoding, multimaterial layering and spatial patterning are also discussed in detail.

Although 4D printing shows great promise for dynamic tissue engineering, personalized adaptive implants, and controlled drug delivery, most studies remain at the proof-of-concept stage. Major challenges, including a limited materials library, low printing precision, simplistic morphing behaviors, non-biocompatible stimuli, lack of predictive simulation tools, and barriers to clinical translation, still constrain progress. In particular, MEW-based 4D printing is still early in development, with critical issues yet to be resolved before its full potential can be realized. This section examines current challenges and potential solutions across three key dimensions: material innovation, fabrication optimization, and actuation control.

### Material Innovation

Bioimplants must simultaneously meet stringent requirements of biocompatibility, biodegradability, and mechanical robustness to ensure long-term safety and functionality in vivo. A key bottleneck lies in material availability, as candidates must balance processability, application-specific responsiveness, and mechanical requirements, while exhibiting programmable degradation kinetics that align with tissue regeneration and avoid toxic byproducts. Beyond passive structural support, next-generation scaffolds should actively direct tissue regeneration, with their chemical, physical, and mechanical properties guiding cellular responses at multiple scales.

As discussed in Sect. 3, processing stimuli-responsive polymers via MEW is constrained by strict requirements for melt viscosity, thermal stability, and low conductivity. SMPs and their composites remain the most extensively studied for bioimplants, with molecular tailoring enabling control over their degradation, hydrophobicity, and mechanical properties. However, many SMPs still suffer from high T_trans_, limited biocompatibility, and insufficient mechanical strength. Progress with biodegradable SMPs, particularly polyester-based copolymers, is promising, though only a few, such as citrate- and glycerol-derived systems, have reached preclinical evaluation. Most SMPs show one-way SME, requiring reprogramming, while two-way or multiple SMEs, achieved via tailored T_trans_ or copolymer architectures, expand opportunities for multifunctional implants with sequential or continuous transformations. For LCE-based implants, the key challenge is achieving mechanical rigidity in defined states without compromising SME performance. Realizing two-way SME near physiological temperatures remains difficult, though molecular engineering approaches have improved structural adaptability and enabled reversible actuation. Hydrogels offer excellent adaptability and responsiveness but lack mechanical robustness for load-bearing implants. Strategies such as double-network design, supramolecular cross-linking, and nanoparticle reinforcement have enhanced their strength and multifunctionality. Continued exploration of biodegradable SMMs [277, 278], functional fillers [168, 279], photocurable systems [9, 280], and processing additives (e.g., rheology modifiers, stabilizers, plasticizers [281]) is essential to expand MEW’s material library and enhance scaffold performance. Current research emphasizes molecular engineering to tune mechanical and shape memory properties, while ensuring compatibility of these new biomaterials with MEW processing is prerequisite.

Moreover, the in vivo behavior of 4D-printed bioimplants within specific tissue environments remains poorly understood. Long-term studies are needed to assess how mechanical and functional performance evolve during degradation to ensure both safety and therapeutic efficacy. Cellular responses to dynamic scaffolds and mechanobiological cues are also underexplored, with most work limited to proof-of-concept rather than in vitro or in vivo validation. Future efforts must clarify how shape morphing influences cell behavior, signaling pathways, and long-term biocompatibility. For clinical translation, systematic evaluation of material–tissue interactions is required to prevent immune rejection, alongside addressing practical challenges such as scalability, sterilization, and shelf life.

### Fabrication Optimization

In MEW, low flow rates (0.5–20 μL h⁻^1^) and syringe-based reservoirs often expose polymers to prolonged heating, causing thermal degradation and limiting material options. Emerging filament-driven MEW systems reduce heating times, improving thermal stability and enabling processing of high-melting-point polymers [125]. For example, modified FFF-MEW printers [6] and open-source MEWron [7] expand material compatibility and multiscale fiber fabrication. Such fine-tuned regulation supports multiscale fiber architectures within a single scaffold, advancing the fabrication of biomimetic implants. Nonetheless, challenges remain in achieving high printing speeds and large-scale scaffolds under low-flow constraints, though dual-nozzle systems show promise for higher throughput [38].

Future optimization requires standardized software for real-time process control and predictive design tools. Machine learning and multiphysics modeling are increasingly applied to regulate parameters and expand 4D printing design space. Multimaterial printing enables complex, site-specific responses but faces hurdles in compatibility, adhesion, and workflow integration. Even in single-material systems, parameter tuning can yield gradient structures as simpler alternatives. Hybrid printing, combining MEW with other advanced techniques, such as inkjet bioprinting and volumetric printing, offers greater versatility but introduces integration and scalability challenges. Replicating precise micro-/nanostructures of native tissues remains challenging, underscoring the need for improved polymer printability and resolution.

### Actuation Control

For 4D-printed bioimplants (e.g., tissue engineering scaffolds, biomedical stents), actuation must match physiological ranges, be rapid, reversible, and repeatable. Achieving such responses in vivo is challenging. Hydrogels lose mechanical strength upon swelling, most SMPs lack intrinsic reversible actuation characteristics, and LCEs require high temperature to trigger reversible actuation. Functional fillers (e.g., magnetic nanoparticles) offer tunable responses, but precise spatiotemporal control, safe activation, and long-term stability remain elusive. As discussed in Sect. 4.6, no single stimulus is universally ideal, thus stimulus selection must be tailored to anatomical location, therapeutic objectives, and implant performance requirements.

In summary, despite promising multifunctional applications, most 4D-printed systems remain far from commercialization. Key barriers include limited SME performance, high costs, low efficiency, and complex regulatory pathways. Clinical translation also demands sterilization methods compatible with low T_trans_ of SMPs, standardized preclinical protocols, and robust regulatory frameworks. Future progress depends on actuation methods suitable for biomedical use, as well as the development of multistimuli-responsive materials for context-specific actuation. Embedding sensors and closed-loop control could further enable real-time adaptive deformation. Meeting stringent demands for biocompatibility, biodegradability, mechanical robustness, multimodal responsiveness, and printing precision will be crucial. With advances in material innovation, fabrication optimization, actuation control, and interdisciplinary collaboration, 4D printing, particularly MEW-based approaches, holds transformative potential for personalized medicine, regenerative therapies, and minimally invasive surgery.

## References

[1] C. Li, C. Guo, V. Fitzpatrick, A. Ibrahim, M.J. Zwierstra et al., Design of biodegradable, implantable devices towards clinical translation. Nat. Rev. Mater. **5**(1), 61–81 (2020). 10.1038/s41578-019-0150-z

[2] W. Wu, J. Wang, G. Li, 3D/4D printing of stimuli-responsive polymers in biomedical engineering: materials, stimulations, and applications. Mater. Sci. Eng. R. Rep. **166**, 101071 (2025). 10.1016/j.mser.2025.101071

[3] P.D. Dalton, Melt electrowriting with additive manufacturing principles. Curr. Opin. Biomed. Eng. **2**, 49–57 (2017). 10.1016/j.cobme.2017.05.007

[4] T.D. Brown, P.D. Dalton, D.W. Hutmacher, Direct writing by way of melt electrospinning. Adv. Mater. **23**(47), 5651–5657 (2011). 10.1002/adma.201103482 22095922 10.1002/adma.201103482

[5] N.K. Karamanos, A.D. Theocharis, Z. Piperigkou, D. Manou, A. Passi et al., A guide to the composition and functions of the extracellular matrix. FEBS J. **288**(24), 6850–6912 (2021). 10.1111/febs.15776 33605520 10.1111/febs.15776

[6] K.M.A. Mueller, A. Hangleiter, S. Burkhardt, D.M. Rojas-González, C. Kwade et al., Filament-based melt electrowriting enables dual-mode additive manufacturing for multiscale constructs. Small Sci. **3**(8), 2300021 (2023). 10.1002/smsc.202300021 40213605 10.1002/smsc.202300021 PMC11935881

[7] A. Reizabal, T. Kangur, P.G. Saiz, S. Menke, C. Moser et al., MEWron: an open-source melt electrowriting platform. Additive Manuf. **71**, 103604 (2023). 10.1016/j.addma.2023.103604

[8] X. Feng, L. Wang, Z. Xue, C. Xie, J. Han et al., Melt electrowriting enabled 3D liquid crystal elastomer structures for cross-scale actuators and temperature field sensors. Sci. Adv. **10**(10), eadk3854 (2024). 10.1126/sciadv.adk3854 38446880 10.1126/sciadv.adk3854 PMC10917348

[9] S.O. Mathew, R. Qi, B.G. Amsden, Thermally stable, photocrossinkable and biocompatible copolymers for melt electrowriting. Biofabrication **17**(4), 045001 (2025). 10.1088/1758-5090/adef81 10.1088/1758-5090/adef81 40659030

[10] E. Yarali, M.J. Mirzaali, A. Ghalayaniesfahani, A. Accardo, P.J. Diaz-Payno et al., 4D printing for biomedical applications. Adv. Mater. **36**(31), 2402301 (2024). 10.1002/adma.202402301 10.1002/adma.202402301 38580291

[11] A. Ding, F. Tang, E. Alsberg, 4D printing: a comprehensive review of technologies, materials, stimuli, design, and emerging applications. Chem. Rev. **125**(7), 3663–3771 (2025). 10.1021/acs.chemrev.4c00070 40106790 10.1021/acs.chemrev.4c00070

[12] A. Ding, F. Tang, E. Alsberg, The emerging 4D printing of shape-memory thermomorphs for self-adaptative biomedical implants. Adv. Funct. Mater. **35**(28), 2418348 (2025). 10.1002/adfm.202418348

[13] X. Wan, Z. Xiao, Y. Tian, M. Chen, F. Liu et al., Recent advances in 4D printing of advanced materials and structures for functional applications. Adv. Mater. **36**(34), 2312263 (2024). 10.1002/adma.202312263 10.1002/adma.202312263 38439193

[14] B. Liu, H. Li, F. Meng, Z. Xu, L. Hao et al., 4D printed hydrogel scaffold with swelling-stiffening properties and programmable deformation for minimally invasive implantation. Nat. Commun. **15**(1), 1587 (2024). 10.1038/s41467-024-45938-0 38383668 10.1038/s41467-024-45938-0 PMC10881973

[15] J. Shi, F. Xia, Q. Tu, C. Wang, Z. Wang et al., Damage-resistant and body-temperature shape memory skin-mimic elastomer for biomedical applications. Sci. Adv. **11**(24), eadv4646 (2025). 10.1126/sciadv.adv4646 40512853 10.1126/sciadv.adv4646 PMC12164989

[16] M. von Witzleben, A. Gasiūnaitė, M. Ihle, A.R. Akkineni, K. Schütz et al., Uniting 4D printing and melt electrowriting for the enhancement of regenerative small diameter vascular grafts. Adv. Healthc. Mater. e02380 (2025). 10.1002/adhm.202502380 10.1002/adhm.202502380 PMC12645082 40772385

[17] J. Wang, J. Zhou, Z. Xie, Y. Zhang, M. He et al., Multifunctional 4D printed shape memory composite scaffolds with photothermal and magnetothermal effects for multimodal tumor therapy and bone repair. Biofabrication **17**(2), 025032 (2025). 10.1088/1758-5090/adc29e 10.1088/1758-5090/adc29e 40106897

[18] K. Mirasadi, M.A. Yousefi, L. Jin, D. Rahmatabadi, M. Baniassadi et al., 4D printing of magnetically responsive shape memory polymers: toward sustainable solutions in soft robotics, wearables, and biomedical devices. Adv. Sci., e13091 (2025). 10.1002/advs.202513091 10.1002/advs.202513091 PMC13042656 40847440

[19] A. Roy, Z. Zhang, M.K. Eiken, A. Shi, A. Pena-Francesch et al., Programmable tissue folding patterns in structured hydrogels. Adv. Mater. **36**(43), 2300017 (2024). 10.1002/adma.202300017 10.1002/adma.202300017 PMC10518030 36961361

[20] Y. Xie, Q. Fang, H. Zhao, Y. Li, Z. Lin et al., Effects of six processing parameters on the size of PCL fibers prepared by melt electrospinning writing. Micromachines **14**(7), 1437 (2023). 10.3390/mi14071437 37512748 10.3390/mi14071437 PMC10385759

[21] A. Hrynevich, B.Ş Elçi, J.N. Haigh, R. McMaster, A. Youssef et al., Dimension-based design of melt electrowritten scaffolds. Small **14**(22), e1800232 (2018). 10.1002/smll.201800232 29707891 10.1002/smll.201800232

[22] G. Hochleitner, T. Jüngst, T.D. Brown, K. Hahn, C. Moseke et al., Additive manufacturing of scaffolds with sub-micron filaments *via* melt electrospinning writing. Biofabrication **7**(3), 035002 (2015). 10.1088/1758-5090/7/3/035002 26065373 10.1088/1758-5090/7/3/035002

[23] C. Großhaus, E. Bakirci, M. Berthel, A. Hrynevich, J.C. Kade et al., Melt electrospinning of nanofibers from medical-grade poly(ε-caprolactone) with a modified nozzle. Small **16**(44), e2003471 (2020). 10.1002/smll.202003471 33048431 10.1002/smll.202003471

[24] C. Blum, J. Weichhold, G. Hochleitner, V. Stepanenko, F. Würthner et al., Controlling topography and crystallinity of melt electrowritten poly(ɛ-caprolactone) fibers. 3D Print. Addit. Manuf. **8**(5), 315–321 (2021). 10.1089/3dp.2020.0290 36654937 10.1089/3dp.2020.0290 PMC9828622

[25] C.B. Dayan, F. Afghah, B.S. Okan, M. Yıldız, Y. Menceloglu et al., Modeling 3D melt electrospinning writing by response surface methodology. Mater. Des. **148**, 87–95 (2018). 10.1016/j.matdes.2018.03.053

[26] Y. Xie, J. Chen, H. Zhao, F. Huang, Prediction of the fiber diameter of melt electrospinning writing by Kriging model. J. Appl. Polym. Sci. **139**(21), 52212 (2022). 10.1002/app.52212

[27] H. Xu, I. Liashenko, A. Lucchetti, L. Du, Y. Dong et al., Designing with circular arc toolpaths to increase the complexity of melt electrowriting. Adv. Mater. Technol. **7**(10), 2101676 (2022). 10.1002/admt.202101676

[28] I. Liashenko, A. Hrynevich, P.D. Dalton, Designing outside the box: unlocking the geometric freedom of melt electrowriting using microscale layer shifting. Adv. Mater. **32**(28), 2001874 (2020). 10.1002/adma.202001874 10.1002/adma.202001874 32459023

[29] Y. Wang, Y. Su, Y. Zhang, M. Chen, High-voltage wave induced a unique structured percolation network with a negative gauge factor. ACS Appl. Mater. Interfaces **14**(4), 5661–5672 (2022). 10.1021/acsami.1c23741 35050585 10.1021/acsami.1c23741

[30] H. Wang, W. Ou, H. Zhong, J. He, Z. Wang et al., Exploring precise deposition and influence mechanism for micro-scale serpentine structure fiber. Adv. Nano Res. **12**, 151–165 (2022). 10.12989/anr.2022.12.2.151

[31] B. Tandon, A.B. Züge, S. Luposchainsky, P.D. Dalton, Effects of electrode design on the melt electrowriting of sinusoidal structures. Adv. Eng. Mater. **25**(17), 2300335 (2023). 10.1002/adem.202300335

[32] C.-V. Nicolae, E. Olăreț, A.-E. Bratu, A. Lungu, I.-C. Stancu et al., Reinforcing melt electrowritten elements with entangled multifibrillar strands for thin hydrogels with potential in bone resurfacing. Mater. Des. **237**, 112545 (2024). 10.1016/j.matdes.2023.112545

[33] F. Eberle, A.-K. Gruska, B. Filippi, P. Stahlhut, G.G. Wallace et al., Hollow-fiber melt electrowriting using a 3D-printed coaxial nozzle. Adv. Eng. Mater. **24**, 2100750 (2022). 10.1002/adem.202100750

[34] Y. Su, Y. Zhang, Y. Chen, S.S. Majidi, M. Dong et al., Surface recrystallization on melt electrowritten scaffolds for acceleration of osteogenic differentiation. Mater. Today Phys. **41**, 101344 (2024). 10.1016/j.mtphys.2024.101344

[35] M. Ryma, T. Tylek, J. Liebscher, C. Blum, R. Fernandez et al., Translation of collagen ultrastructure to biomaterial fabrication for material-independent but highly efficient topographic immunomodulation. Adv. Mater. **33**(33), 2101228 (2021). 10.1002/adma.202101228 34240485 10.1002/adma.202101228 PMC11468812

[36] J.C. Kade, P.F. Otto, R. Luxenhofer, P.D. Dalton, Melt electrowriting of poly(vinylidene difluoride) using a heated collector. Polym. Adv. Technol. **32**(12), 4951–4955 (2021). 10.1002/pat.5463

[37] H. Haag, D. Sonnleitner, G. Lang, P.D. Dalton, Melt electrowriting to produce microfiber fragments. Polym. Adv. Technol. **33**(6), 1989–1992 (2022). 10.1002/pat.5641

[38] R.S. Diaz, E.M. De-Juan-Pardo, P.D. Dalton, T.R. Dargaville, Semi-woven structures *via* dual nozzle melt electrowriting. Macromol. Mater. Eng. **308**(4), 2200526 (2023). 10.1002/mame.202200526

[39] P.B. Warren, Z.G. Davis, M.B. Fisher, Parametric control of fiber morphology and tensile mechanics in scaffolds with high aspect ratio geometry produced *via* melt electrowriting for musculoskeletal soft tissue engineering. J. Mech. Behav. Biomed. Mater. **99**, 153–160 (2019). 10.1016/j.jmbbm.2019.07.013 31352215 10.1016/j.jmbbm.2019.07.013

[40] J. Kim, E. Bakirci, K.L. O’Neill, A. Hrynevich, P.D. Dalton, Fiber bridging during melt electrowriting of poly(ε-caprolactone) and the influence of fiber diameter and wall height. Macromol. Mater. Eng. **306**(3), 2000685 (2021). 10.1002/mame.202000685

[41] L.D. Brenna, P. Edmund, C.A. Mark, C.P. Naomi, A.W. Maria, Advancing scaffold biomimicry: engineering mechanics in microfiber scaffolds with independently controlled architecture using melt electrowriting. bioRxiv (2023). 10.1101/2023.05.28.542676

[42] B.L. Devlin, S. Kuba, P.C. Hall, A.B. McCosker, E. Pickering et al., A melt electrowriting toolbox for automated G-code generation and toolpath correction of flat and tubular constructs. Adv. Mater. Technol. **9**(22), 2400419 (2024). 10.1002/admt.202400419

[43] E. Bakirci, N. Schaefer, O. Dahri, A. Hrynevich, P. Strissel et al., Melt electrowritten *in vitro* radial device to study cell growth and migration. Adv. Biosyst. **4**(10), e2000077 (2020). 10.1002/adbi.202000077 32875734 10.1002/adbi.202000077

[44] B.N. Jensen, Y. Wang, A. Le Friec, S. Nabavi, M. Dong et al., Wireless electromagnetic neural stimulation patch with anisotropic guidance. NPJ Flex. Electron. **7**, 34 (2023). 10.1038/s41528-023-00270-3

[45] F.M. Wunner, M.-L. Wille, T.G. Noonan, O. Bas, P.D. Dalton et al., Melt electrospinning writing of highly ordered large volume scaffold architectures. Adv. Mater. **30**(20), e1706570 (2018). 10.1002/adma.201706570 29633443 10.1002/adma.201706570

[46] G. Zheng, G. Fu, J. Jiang, X. Wang, W. Li et al., Melt electrowriting stacked architectures with high aspect ratio. Appl. Phys. A **127**(6), 410 (2021). 10.1007/s00339-021-04582-x

[47] C.D. Lamb, B. Maitland, M.S. Hepburn, T.R. Dargaville, B.F. Kennedy et al., Understanding the significance of layer bonding in melt electrowriting. Adv. Sci. **11**(47), 2407514 (2024). 10.1002/advs.202407514 10.1002/advs.202407514 PMC11653759 39447154

[48] Y. Su, Z. Zhang, Y. Wan, Y. Zhang, Z. Wang et al., A hierarchically ordered compacted coil scaffold for tissue regeneration. NPG Asia Mater. **12**, 55 (2020). 10.1038/s41427-020-0234-7

[49] A. Hrynevich, P. Achenbach, T. Jungst, G.A. Brook, P.D. Dalton, Design of suspended melt electrowritten fiber arrays for schwann cell migration and neurite outgrowth. Macromol. Biosci. **21**(7), 2000439 (2021). 10.1002/mabi.202000439 10.1002/mabi.202000439 33951291

[50] C. Xie, Q. Gao, P. Wang, L. Shao, H. Yuan et al., Structure-induced cell growth by 3D printing of heterogeneous scaffolds with ultrafine fibers. Mater. Des. **181**, 108092 (2019). 10.1016/j.matdes.2019.108092

[51] N. Abbasi, S. Ivanovski, K. Gulati, R.M. Love, S. Hamlet, Role of offset and gradient architectures of 3-D melt electrowritten scaffold on differentiation and mineralization of osteoblasts. Biomater. Res. **24**, 2 (2020). 10.1186/s40824-019-0180-z 31911842 10.1186/s40824-019-0180-z PMC6942301

[52] N. Abbasi, A. Abdal-hay, S. Hamlet, E. Graham, S. Ivanovski, Effects of gradient and offset architectures on the mechanical and biological properties of 3-D melt electrowritten (MEW) scaffolds. ACS Biomater. Sci. Eng. **5**(7), 3448–3461 (2019). 10.1021/acsbiomaterials.8b01456 33405729 10.1021/acsbiomaterials.8b01456

[53] M.K. Włodarczyk-Biegun, M. Villiou, M. Koch, C. Muth, P. Wang et al., Melt electrowriting of graded porous scaffolds to mimic the matrix structure of the human trabecular meshwork. ACS Biomater. Sci. Eng. **8**(9), 3899–3911 (2022). 10.1021/acsbiomaterials.2c00623 35984428 10.1021/acsbiomaterials.2c00623 PMC9472227

[54] M. Shahverdi, S. Seifi, A. Akbari, K. Mohammadi, A. Shamloo et al., Melt electrowriting of PLA, PCL, and composite PLA/PCL scaffolds for tissue engineering application. Sci. Rep. **12**(1), 19935 (2022). 10.1038/s41598-022-24275-6 36402790 10.1038/s41598-022-24275-6 PMC9675866

[55] M.J. Vernon, J. Lu, B. Padman, C. Lamb, R. Kent et al., Engineering heart valve interfaces using melt electrowriting: biomimetic design strategies from multi-modal imaging. Adv. Healthc. Mater. **11**(24), 2201028 (2022). 10.1002/adhm.202201028 36300603 10.1002/adhm.202201028 PMC11468946

[56] C.D. O’Connell, O. Bridges, C. Everett, N. Antill-O’Brien, C. Onofrillo et al., Electrostatic distortion of melt-electrowritten patterns by 3D objects: quantification, modeling, and toolpath correction. Adv. Mater. Technol. **6**(11), 2100345 (2021). 10.1002/admt.202100345

[57] U. Saha, R. Nairn, O. Keenan, M.G. Monaghan, A deeper insight into the influence of the electric field strength when melt-electrowriting on non-planar surfaces. Macromol. Mater. Eng. **306**(12), 2100496 (2021). 10.1002/mame.202100496

[58] Q.C. Peiffer, M. de Ruijter, J. van Duijn, D. Crottet, E. Dominic et al., Melt electrowriting onto anatomically relevant biodegradable substrates: resurfacing a diarthrodial joint. Mater. Des. **195**, 109025 (2020). 10.1016/j.matdes.2020.109025 33088011 10.1016/j.matdes.2020.109025 PMC7116215

[59] A. Zaeri, K. Cao, F. Zhang, R. Zgeib, R.C. Chang, Design and fabrication of fibrous spindle-like constructs using a melt electrohydrodynamic writing process. Macromol. Mater. Eng. **309**(11), 2400080 (2024). 10.1002/mame.202400080

[60] M. von Witzleben, T. Stoppe, A. Zeinalova, Z. Chen, T. Ahlfeld et al., Multimodal additive manufacturing of biomimetic tympanic membrane replacements with near tissue-like acousto-mechanical and biological properties. Acta Biomater. **170**, 124–141 (2023). 10.1016/j.actbio.2023.09.005 37696412 10.1016/j.actbio.2023.09.005

[61] P. Terranova, K.M.A. Mueller, D. Biebl, A. D’Amore, P. Mela, A versatile 5-axis melt electrowriting platform for unprecedented design freedom of 3D fibrous scaffolds. Addit. Manuf. **93**, 104431 (2024). 10.1016/j.addma.2024.104431

[62] F. Zhang, K. Cao, A. Zaeri, R. Zgeib, R.C. Chang, The design and fabrication of engineered tubular tissue constructs enabled by electrohydrodynamic fabrication techniques: a review. Macromol. Mater. Eng. **309**(9), 2400095 (2024). 10.1002/mame.202400095

[63] A.M. van Genderen, K. Jansen, M. Kristen, J. van Duijn, Y. Li et al., Topographic guidance in melt-electrowritten tubular scaffolds enhances engineered kidney tubule performance. Front. Bioeng. Biotechnol. **8**, 617364 (2021). 10.3389/fbioe.2020.617364 33537294 10.3389/fbioe.2020.617364 PMC7848123

[64] E. McColl, J. Groll, T. Jungst, P.D. Dalton, Design and fabrication of melt electrowritten tubes using intuitive software. Mater. Des. **155**, 46–58 (2018). 10.1016/j.matdes.2018.05.036

[65] F. Zhang, K. Cao, A. Zaeri, R. Zgeib, C. Buckley et al., Design, fabrication, and characterization of tubular scaffolds by way of a melt electrowriting process. Addit. Manuf. **62**, 103383 (2023). 10.1016/j.addma.2022.103383

[66] N.C. Paxton, R. Daley, D.P. Forrestal, M.C. Allenby, M.A. Woodruff, Auxetic tubular scaffolds *via* melt electrowriting. Mater. Des. **193**, 108787 (2020). 10.1016/j.matdes.2020.108787

[67] A.B. McCosker, M.E. Snowdon, R. Lamont, M.A. Woodruff, N.C. Paxton, Exploiting nonlinear fiber patterning to control tubular scaffold mechanical behavior. Adv. Mater. Technol. **7**(11), 2200259 (2022). 10.1002/admt.202200259

[68] E. Pickering, N.C. Paxton, A. Bo, B. O’Connell, M. King et al., 3D printed tubular scaffolds with massively tailorable mechanical behavior. Adv. Eng. Mater. **24**(11), 2200479 (2022). 10.1002/adem.202200479

[69] N.C. Paxton, M. Lanaro, A. Bo, N. Crooks, M.T. Ross et al., Design tools for patient specific and highly controlled melt electrowritten scaffolds. J. Mech. Behav. Biomed. Mater. **105**, 103695 (2020). 10.1016/j.jmbbm.2020.103695 32090895 10.1016/j.jmbbm.2020.103695

[70] P. Mieszczanek, T.M. Robinson, P.D. Dalton, D.W. Hutmacher, Convergence of machine vision and melt electrowriting. Adv. Mater. **33**(29), 2100519 (2021). 10.1002/adma.202100519 34101929 10.1002/adma.202100519 PMC11468355

[71] Z. Peng, M. Wang, H. Lv, J. Zhang, Y. Li et al., Electric field-driven microscale 3D printing of flexible thin-walled tubular mesh structures of molten polymers. Mater. Des. **225**, 111433 (2023). 10.1016/j.matdes.2022.111433

[72] Q.S. Thorsnes, P.R. Turner, M.A. Ali, J.D. Cabral, Integrating fused deposition modeling and melt electrowriting for engineering branched vasculature. Biomedicines **11**(12), 3139 (2023). 10.3390/biomedicines11123139 38137359 10.3390/biomedicines11123139 PMC10740633

[73] N.T. Saidy, T. Shabab, O. Bas, D.M. Rojas-González, M. Menne et al., Melt electrowriting of complex 3D anatomically relevant scaffolds. Front. Bioeng. Biotechnol. **8**, 793 (2020). 10.3389/fbioe.2020.00793 32850700 10.3389/fbioe.2020.00793 PMC7396698

[74] T.L. Brooks-Richards, N.C. Paxton, M.C. Allenby, M.A. Woodruff, Dissolvable 3D printed PVA moulds for melt electrowriting tubular scaffolds with patient-specific geometry. Mater. Des. **215**, 110466 (2022). 10.1016/j.matdes.2022.110466

[75] S. Loewner, S. Heene, T. Baroth, H. Heymann, F. Cholewa et al., Recent advances in melt electro writing for tissue engineering for 3D printing of microporous scaffolds for tissue engineering. Front. Bioeng. Biotechnol. **10**, 896719 (2022). 10.3389/fbioe.2022.896719 36061443 10.3389/fbioe.2022.896719 PMC9428513

[76] S. Ashour, H. Xu, Melt electrowriting: a study of jet diameters and jet speeds along the spinline. Polym. Adv. Technol. **33**(9), 3013–3016 (2022). 10.1002/pat.5755

[77] K. Cao, F. Zhang, A. Zaeri, Y. Zhang, R. Zgeib et al., Advances in design and quality of melt electrowritten scaffolds. Mater. Des. **226**, 111618 (2023). 10.1016/j.matdes.2023.111618

[78] F. Tourlomousis, H. Ding, D.M. Kalyon, R.C. Chang, Melt electrospinning writing process guided by a “printability number.” J. Manuf. Sci. Eng. **139**(8), 081004 (2017). 10.1115/1.4036348

[79] K. Cao, F. Zhang, A. Zaeri, R. Zgeib, R.C. Chang, Advancing a real-time image-based jet lag tracking methodology for optimizing print parameters and assessing melt electrowritten fiber quality. Addit. Manuf. **54**, 102764 (2022). 10.1016/j.addma.2022.102764

[80] H. Ding, K. Cao, F. Zhang, W. Boettcher, R.C. Chang, A fundamental study of charge effects on melt electrowritten polymer fibers. Mater. Des. **178**, 107857 (2019). 10.1016/j.matdes.2019.107857

[81] L. Du, L. Nie, L. Zhang, H. Lu, L. Yang et al., Enhancing the printing accuracy of melt electrowritten fibers deposited on aluminum foils. Mater. Lett. **321**, 132397 (2022). 10.1016/j.matlet.2022.132397

[82] H. Lu, Y. Sun, Y. Chen, L. Nie, L. Yang et al., The effects of voltage configurations on print accuracy in melt electrowriting. Mater. Lett. **334**, 133738 (2023). 10.1016/j.matlet.2022.133738

[83] F. Zhang, K. Cao, A. Zaeri, R. Zgeib, R.C. Chang, Effects of scaffold design parameters on the printing accuracy for melt electrowriting. J. Manuf. Process. **81**, 177–190 (2022). 10.1016/j.jmapro.2022.06.070

[84] F. Zhang, K. Cao, A. Zaeri, R. Zgeib, R.C. Chang, Effects of printing sequence on the printing accuracy of melt electrowriting scaffolds. Macromol. Mater. Eng. **307**(9), 2200222 (2022). 10.1002/mame.202200222

[85] A. Hrynevich, I. Liashenko, P.D. Dalton, Accurate prediction of melt electrowritten laydown patterns from simple geometrical considerations. Adv. Mater. Technol. **5**(12), 2000772 (2020). 10.1002/admt.202000772

[86] K. Cao, F. Zhang, B. Wang, Y. Sun, A. Zaeri et al., Analytical interpretation of microscale fiber deviation in designing for polymer melt electrohydrodynamic-based additive manufacturing. Addit. Manuf. **58**, 103035 (2022). 10.1016/j.addma.2022.103035

[87] Y. Jin, Q. Gao, C. Xie, G. Li, J. Du et al., Fabrication of heterogeneous scaffolds using melt electrospinning writing: design and optimization. Mater. Des. **185**, 108274 (2020). 10.1016/j.matdes.2019.108274

[88] K. Cao, F. Zhang, A. Zaeri, R. Zgeib, R.C. Chang, Quantitative investigation into the design and process parametric effects on the fiber-entrapped residual charge for a polymer melt electrohydrodynamic printing process. Macromol. Mater. Eng. **307**(3), 2100766 (2022). 10.1002/mame.202100766

[89] K. Cao, F. Zhang, R.C. Chang, A charge-based mechanistic study into the effects of process parameters on fiber accumulating geometry for a melt electrohydrodynamic process. Processes **8**(11), 1440 (2020). 10.3390/pr8111440

[90] K. Cao, F. Zhang, A. Zaeri, R. Zgeib, R.C. Chang, A charge‐based mechanistic study into the effect of collector temperature on melt electrohydrodynamic printing outcomes. Adv. Mater. Technol. **6**(7), 2100251 (2021). 10.1002/admt.202100251

[91] K. Cao, F. Zhang, A. Zaeri, R. Zgeib, R.C. Chang, A holistic model for melt electrowritten three-dimensional structured materials based on residual charge. Int. J. Bioprinting **9**, 656 (2022). 10.18063/ijb.v9i2.656 10.18063/ijb.v9i2.656 PMC10090534 37065672

[92] L. Du, L. Yang, H. Lu, L. Nie, Y. Sun et al., Additive manufacturing of ultrahigh-resolution poly(ε-caprolactone) scaffolds using melt electrowriting. Polymer **301**, 127028 (2024). 10.1016/j.polymer.2024.127028

[93] Z. Zou, Y. Wang, Z. Shen, N. Luo, Study on suppression strategy of jet lag effect in melt electrowriting. J. Mech. Sci. Technol. **37**(9), 4801–4808 (2023). 10.1007/s12206-023-0832-8

[94] X. Kuang, D.J. Roach, J. Wu, C.M. Hamel, Z. Ding et al., Advances in 4D printing: materials and applications. Adv. Funct. Mater. **29**(2), 1805290 (2019). 10.1002/adfm.201805290

[95] H. Xu, L. Du, Sustainable medical materials printed by melt electrowriting: a mini-review. Curr. Opin. Biomed. Eng. **27**, 100464 (2023). 10.1016/j.cobme.2023.100464

[96] J.C. Kade, P.D. Dalton, Polymers for melt electrowriting. Adv. Healthc. Mater. **10**, 2001232 (2021). 10.1002/adhm.202001232 32940962 10.1002/adhm.202001232 PMC11469188

[97] N.C. Paxton, S.W.K. Ho, B.T. Tuten, J. Lipton-Duffin, M.A. Woodruff, Degradation of melt electrowritten PCL scaffolds following melt processing and plasma surface treatment. Macromol. Rapid Commun. **42**(23), e2100433 (2021). 10.1002/marc.202100433 34668263 10.1002/marc.202100433

[98] C. Böhm, P. Stahlhut, J. Weichhold, A. Hrynevich, J. Teßmar et al., The multiweek thermal stability of medical-grade poly(ε-caprolactone) during melt electrowriting. Small **18**(3), 2104193 (2022). 10.1002/smll.202104193 10.1002/smll.202104193 34741411

[99] J. Delaey, P. Dubruel, S. Van Vlierberghe, Shape-memory polymers for biomedical applications. Adv. Funct. Mater. **30**(44), 1909047 (2020). 10.1002/adfm.201909047

[100] P. Feng, F. Yang, J. Jia, J. Zhang, W. Tan et al., Mechanism and manufacturing of 4D printing: derived and beyond the combination of 3D printing and shape memory material. Int. J. Extrem. Manuf. **6**(6), 062011 (2024). 10.1088/2631-7990/ad7e5f

[101] W.M. Huang, Z. Ding, C.C. Wang, J. Wei, Y. Zhao et al., Shape memory materials. Mater. Today **13**(7–8), 54–61 (2010). 10.1016/S1369-7021(10)70128-0

[102] S. Yang, Y. He, Z. Song, Y. Li, Research status and potential direction for thermoplastic shape memory polymers and composites: a review. Polymers **17**(10), 1360 (2025). 10.3390/polym17101360 40430656 10.3390/polym17101360 PMC12114747

[103] A. Lendlein, R. Langer, Biodegradable, elastic shape-memory polymers for potential biomedical applications. Science **296**(5573), 1673–1676 (2002). 10.1126/science.1066102 11976407 10.1126/science.1066102

[104] A. Lendlein, P. Neuenschwander, U.W. Suter, Tissue-compatible multiblock copolymers for medical applications, controllable in degradation rate and mechanical properties. Macromol. Chem. Phys. **199**, 2785–2796 (1998). 10.1002/(SICI)1521-3935(19981201)199:12<2785::AID-MACP2785>3.3.CO;2-O

[105] M. Balk, M. Behl, C. Wischke, J. Zotzmann, A. Lendlein, Recent advances in degradable lactide-based shape-memory polymers. Adv. Drug Deliv. Rev. **107**, 136–152 (2016). 10.1016/j.addr.2016.05.012 27262926 10.1016/j.addr.2016.05.012

[106] W. Zhao, C. Yue, L. Liu, Y. Liu, J. Leng, Research progress of shape memory polymer and 4D printing in biomedical application. Adv. Healthc. Mater. **12**(16), 2201975 (2023). 10.1002/adhm.202201975 10.1002/adhm.202201975 36520058

[107] B.Q.Y. Chan, Z.W.K. Low, S.J.W. Heng, S.Y. Chan, C. Owh et al., Recent advances in shape memory soft materials for biomedical applications. ACS Appl. Mater. Interfaces **8**(16), 10070–10087 (2016). 10.1021/acsami.6b01295 27018814 10.1021/acsami.6b01295

[108] F. Zhang, N. Wen, L. Wang, Y. Bai, J. Leng, Design of 4D printed shape-changing tracheal stent and remote controlling actuation. Int. J. Smart Nano Mater. **12**(4), 375–389 (2021). 10.1080/19475411.2021.1974972

[109] C. Wischke, M. Behl, A. Lendlein, Drug-releasing shape-memory polymers–the role of morphology, processing effects, and matrix degradation. Expert Opin. Drug Deliv. **10**(9), 1193–1205 (2013). 10.1517/17425247.2013.797406 23668314 10.1517/17425247.2013.797406

[110] A. Lendlein, S. Kelch, Shape-memory polymers. Angew. Chem. Int. Ed. **41**(12), 2034–2057 (2002). 10.1002/1521-3773 19746597

[111] A. Lendlein, O.E.C. Gould, Reprogrammable recovery and actuation behaviour of shape-memory polymers. Nat. Rev. Mater. **4**(2), 116–133 (2019). 10.1038/s41578-018-0078-8

[112] M. Bodaghi, A.R. Damanpack, W.H. Liao, Adaptive metamaterials by functionally graded 4D printing. Mater. Des. **135**, 26–36 (2017). 10.1016/j.matdes.2017.08.069

[113] T. Xie, Recent advances in polymer shape memory. Polymer **52**(22), 4985–5000 (2011). 10.1016/j.polymer.2011.08.003

[114] Y. Xia, Y. He, F. Zhang, Y. Liu, J. Leng, A review of shape memory polymers and composites: mechanisms, materials, and applications. Adv. Mater. **33**(6), 2000713 (2021). 10.1002/adma.202000713 10.1002/adma.202000713 32969090

[115] Z. Shao, H. Chen, Q. Wang, G. Kang, J. Jiang et al., Melt electrowriting ordered TPU microfibrous mesh for on-demand colorimetric wearable sweat detection. IEEE Sens. J. **22**(19), 18560–18566 (2022). 10.1109/JSEN.2022.3199406

[116] C. Pasini, Z.V. Soreño, D. Schönfeld, T. Pretsch, G. Constante et al., 4D fabrication of two-way shape memory polymeric composites by electrospinning and melt electrowriting. Macromol. Rapid Commun. **45**(11), 2400010 (2024). 10.1002/marc.202400010 10.1002/marc.202400010 38458610

[117] M. Xue, W. Zhang, H. Jin, H. Wu, B. Qiu et al., Composite additive manufacturing for suspended microelectrode arrays: advancing oriented myocardial tissue culturing and electrophysiological sensing. Biosens. Bioelectron. **287**, 117686 (2025). 10.1016/j.bios.2025.117686 40523322 10.1016/j.bios.2025.117686

[118] L. Nie, Y. Sun, X. Ming, Z. Xu, X. Ye et al., High-resolution 3D printed strain sensor with superb stretchability and sensitivity: unveiling the potential of melt electrowriting. Mater. Today **84**, 39–47 (2025). 10.1016/j.mattod.2025.01.017

[119] D.C.S. Costa, P.D.C. Costa, M.C. Gomes, A. Chandrakar, P.A. Wieringa et al., Universal strategy for designing shape memory hydrogels. ACS Mater. Lett. **4**(4), 701–706 (2022). 10.1021/acsmaterialslett.2c00107 36568348 10.1021/acsmaterialslett.2c00107 PMC9777886

[120] G. Constante, I. Apsite, P. Auerbach, S. Aland, D. Schönfeld et al., Smart mechanically tunable surfaces with shape memory behavior and wetting-programmable topography. ACS Appl. Mater. Interfaces **14**(17), 20208–20219 (2022). 10.1021/acsami.2c01078 35438953 10.1021/acsami.2c01078

[121] G. Constante, I. Apsite, D. Schönfeld, T. Pretsch, L. Ionov, Reversibly photoswitchable high-aspect ratio surfaces. Small Struct. **4**(10), 2300040 (2023). 10.1002/sstr.202300040

[122] H. Ramaraju, R.E. Akman, D.L. Safranski, S.J. Hollister, Designing biodegradable shape memory polymers for tissue repair. Adv. Funct. Mater. **30**(44), 2002014 (2020). 10.1002/adfm.202002014

[123] N. Roudbarian, M. Baniasadi, P. Nayyeri, M. Ansari, R. Hedayati et al., Enhancing shape memory properties of multi-layered and multi-material polymer composites in 4D printing. Smart Mater. Struct. **30**(10), 105006 (2021). 10.1088/1361-665X/ac1b3b

[124] M. Ramezani, D. Getya, I. Gitsov, M.B.B. Monroe, Solvent-free synthesis of biostable segmented polyurethane shape memory polymers for biomedical applications. J. Mater. Chem. B **12**(5), 1217–1231 (2024). 10.1039/D3TB02472E 38168979 10.1039/d3tb02472e

[125] T. Sun, H. Lu, S. Luposchainsky, L. Yang, X. Zhang et al., Challenges of high-temperature melt electrowriting: a study of EVOH printing. Polymer **331**, 128518 (2025). 10.1016/j.polymer.2025.128518

[126] D.J. Roach, X. Kuang, C. Yuan, K. Chen, H.J. Qi, Novel ink for ambient condition printing of liquid crystal elastomers for 4D printing. Smart Mater. Struct. **27**(12), 125011 (2018). 10.1088/1361-665x/aae96f

[127] T.J. White, D.J. Broer, Programmable and adaptive mechanics with liquid crystal polymer networks and elastomers. Nat. Mater. **14**(11), 1087–1098 (2015). 10.1038/nmat4433 26490216 10.1038/nmat4433

[128] T. Guin, M.J. Settle, B.A. Kowalski, A.D. Auguste, R.V. Beblo et al., Layered liquid crystal elastomer actuators. Nat. Commun. **9**, 2531 (2018). 10.1038/s41467-018-04911-4 29955053 10.1038/s41467-018-04911-4 PMC6023890

[129] M. Chen, M. Gao, L. Bai, H. Zheng, H.J. Qi et al., Recent advances in 4D printing of liquid crystal elastomers. Adv. Mater. **35**(23), e2209566 (2023). 10.1002/adma.202209566 36461147 10.1002/adma.202209566

[130] K.M. Herbert, H.E. Fowler, J.M. McCracken, K.R. Schlafmann, J.A. Koch et al., Synthesis and alignment of liquid crystalline elastomers. Nat. Rev. Mater. **7**(1), 23–38 (2022). 10.1038/s41578-021-00359-z

[131] M. Javadzadeh, J. del Barrio, C. Sánchez-Somolinos, Melt electrowriting of liquid crystal elastomer scaffolds with programmed mechanical response. Adv. Mater. **35**(14), 2209244 (2023). 10.1002/adma.202209244 10.1002/adma.202209244 36459991

[132] D. Roach, C. Yuan, X. Kuang, V.C. Li, P. Blake et al., Long liquid crystal elastomer fibers with large reversible actuation strains for smart textiles and artificial muscles. ACS Appl. Mater. Interfaces **11**(21), 19514–19521 (2019). 10.1021/acsami.9b04401 31062572 10.1021/acsami.9b04401

[133] C. Zhang, X. Lu, G. Fei, Z. Wang, H. Xia et al., 4D printing of a liquid crystal elastomer with a controllable orientation gradient. ACS Appl. Mater. Interfaces **11**(47), 44774–44782 (2019). 10.1021/acsami.9b18037 31692319 10.1021/acsami.9b18037

[134] C.P. Ambulo, J.J. Burroughs, J.M. Boothby, H. Kim, M.R. Shankar et al., Four-dimensional printing of liquid crystal elastomers. ACS Appl. Mater. Interfaces **9**(42), 37332–37339 (2017). 10.1021/acsami.7b11851 28967260 10.1021/acsami.7b11851

[135] A. Kotikian, R.L. Truby, J.W. Boley, T.J. White, J.A. Lewis, 3D printing of liquid crystal elastomeric actuators with spatially programed nematic order. Adv. Mater. **30**(10), 1706164 (2018). 10.1002/adma.201706164 10.1002/adma.201706164 29334165

[136] X. Yin, L. Li, Y.-X. Zhao, Z.-Y. Xu, L.-Y. Shi et al., Adaptive structural regulation of disulfide contained liquid crystal elastomers for mild temperature-induced two-way shape memory effects. Macromolecules **58**(12), 6005–6016 (2025). 10.1021/acs.macromol.5c01057

[137] A. Ding, O. Jeon, R. Tang, Y.B. Lee, S.J. Lee et al., Cell-laden multiple-step and reversible 4d hydrogel actuators to mimic dynamic tissue morphogenesis. Adv. Sci. **8**(9), 2004616 (2021). 10.1002/advs.202004616 10.1002/advs.202004616 PMC8097354 33977070

[138] P.J. Díaz-Payno, M. Kalogeropoulou, I. Muntz, E. Kingma, N. Kops et al., Swelling-dependent shape-based transformation of a human mesenchymal stromal cells-laden 4d bioprinted construct for cartilage tissue engineering. Adv. Healthc. Mater. **12**(2), 2201891 (2023). 10.1002/adhm.202201891 36308047 10.1002/adhm.202201891 PMC11468569

[139] M. Hippler, K. Weißenbruch, K. Richler, E.D. Lemma, M. Nakahata et al., Mechanical stimulation of single cells by reversible host-guest interactions in 3D microscaffolds. Sci. Adv. **6**(39), eabc2648 (2020). 10.1126/sciadv.abc2648 32967835 10.1126/sciadv.abc2648 PMC7531888

[140] D.J. Wu, N.H. Vonk, B.A.G. Lamers, M. Castilho, J. Malda et al., Anisotropic hygro-expansion in hydrogel fibers owing to uniting 3D electrowriting and supramolecular polymer assembly. Eur. Polym. J. **141**, 110099 (2020). 10.1016/j.eurpolymj.2020.110099

[141] D. Nahm, F. Weigl, N. Schaefer, A. Sancho, A. Frank et al., A versatile biomaterial ink platform for the melt electrowriting of chemically-crosslinked hydrogels. Mater. Horiz. **7**(3), 928–933 (2020). 10.1039/C9MH01654F

[142] Z. Kroneková, T. Lorson, J. Kronek, R. Luxenhofer, Cytotoxicity of 2-oxazines and poly(2-oxazine)s in mouse fibroblast. ChemRxiv (2018). 10.26434/chemrxiv.5793990.v1

[143] C.M. Nimmo, S.C. Owen, M.S. Shoichet, Diels-alder click cross-linked hyaluronic acid hydrogels for tissue engineering. Biomacromol **12**(3), 824–830 (2011). 10.1021/bm101446k 10.1021/bm101446k 21314111

[144] A. Cortés, A. Cosola, M. Sangermano, M. Campo, S. González Prolongo et al., DLP 4D-printing of remotely, modularly, and selectively controllable shape memory polymer nanocomposites embedding carbon nanotubes. Adv. Funct. Mater. **31**(50), 2106774 (2021). 10.1002/adfm.202106774

[145] C. Zeng, L. Liu, W. Bian, Y. Liu, J. Leng, 4D printed electro-induced continuous carbon fiber reinforced shape memory polymer composites with excellent bending resistance. Compos. Part B Eng. **194**, 108034 (2020). 10.1016/j.compositesb.2020.108034

[146] Z. Meng, J. He, Z. Xia, D. Li, Fabrication of microfibrous PCL/MWCNTs scaffolds *via* melt-based electrohydrodynamic printing. Mater. Lett. **278**, 128440 (2020). 10.1016/j.matlet.2020.128440

[147] Y. Zhang, A. Le Friec, Z. Zhang, C.A. Müller, T. Du et al., Electroactive biomaterials synergizing with electrostimulation for cardiac tissue regeneration and function-monitoring. Mater. Today **70**, 237–272 (2023). 10.1016/j.mattod.2023.09.005

[148] C. Zhang, X. Li, L. Jiang, D. Tang, H. Xu et al., 3D printing of functional magnetic materials: from design to applications. Adv. Funct. Mater. **31**(34), 2102777 (2021). 10.1002/adfm.202102777

[149] E. Yarali, M. Baniasadi, A. Zolfagharian, M. Chavoshi, F. Arefi et al., Magneto-/ electro-responsive polymers toward manufacturing, characterization, and biomedical/soft robotic applications. Appl. Mater. Today **26**, 101306 (2022). 10.1016/j.apmt.2021.101306

[150] V.Q. Nguyen, A.S. Ahmed, R.V. Ramanujan, Morphing soft magnetic composites. Adv. Mater. **24**(30), 4041–4054 (2012). 10.1002/adma.201104994 22760813 10.1002/adma.201104994

[151] V. Frantellizzi, M. Conte, M. Pontico, A. Pani, R. Pani et al., New frontiers in molecular imaging with superparamagnetic iron oxide nanoparticles (SPIONs): efficacy, toxicity, and future applications. Nucl. Med. Mol. Imaging **54**(2), 65–80 (2020). 10.1007/s13139-020-00635-w 32377258 10.1007/s13139-020-00635-w PMC7198685

[152] K.M.A. Mueller, G.J. Topping, S.P. Schwaminger, Y. Zou, D.M. Rojas-González et al., Visualization of USPIO-labeled melt-electrowritten scaffolds by non-invasive magnetic resonance imaging. Biomater. Sci. **9**(13), 4607–4612 (2021). 10.1039/d1bm00461a 34096938 10.1039/d1bm00461a

[153] I. Unalan, I. Occhipinti, M. Miola, E. Vernè, A.R. Boccaccini, Development of super-paramagnetic iron oxide nanoparticle-coated melt electrowritten scaffolds for biomedical applications. Macromol. Biosci. **24**(3), e2300397 (2024). 10.1002/mabi.202300397 37902248 10.1002/mabi.202300397 PMC13420971

[154] S. Mansi, S.V. Dummert, G.J. Topping, M.Z. Hussain, C. Rickert et al., Introducing metal–organic frameworks to melt electrowriting: multifunctional scaffolds with controlled microarchitecture for tissue engineering applications. Adv. Funct. Mater. **34**(2), 2304907 (2024). 10.1002/adfm.202304907

[155] J.C. Kade, E. Bakirci, B. Tandon, D. Gorgol, M. Mrlik et al., The impact of including carbonyl iron particles on the melt electrowriting process. Macromol. Mater. Eng. **307**(12), 2200478 (2022). 10.1002/mame.202200478

[156] P.G. Saiz, A. Reizabal, S. Luposchainsky, J.L. Vilas-Vilela, S. Lanceros-Mendez et al., Magnetically responsive melt electrowritten structures. Adv. Mater. Technol. **8**(13), 2202063 (2023). 10.1002/admt.202202063

[157] G. Cedillo-Servin, O. Dahri, J. Meneses, J. van Duijn, H. Moon et al., 3D printed magneto-active microfiber scaffolds for remote stimulation and guided organization of 3D *in vitro* skeletal muscle models. Small **20**(12), 2307178 (2024). 10.1002/smll.202307178 10.1002/smll.202307178 37950402

[158] M. Tang, S. Mahri, Y.-P. Shiau, T. Mukarrama, R. Villa et al., Multifunctional and scalable nanoparticles for bimodal image-guided phototherapy in bladder cancer treatment. Nano-Micro Lett. **17**(1), 222 (2025). 10.1007/s40820-025-01717-0 10.1007/s40820-025-01717-0 PMC12008111 40249569

[159] D. Habault, H. Zhang, Y. Zhao, Light-triggered self-healing and shape-memory polymers. Chem. Soc. Rev. **42**(17), 7244 (2013). 10.1039/c3cs35489j 23440057 10.1039/c3cs35489j

[160] X. Wu, T. Vedelaar, R. Li, R. Schirhagl, M. Kamperman et al., Melt electrowritten scaffolds containing fluorescent nanodiamonds for improved mechanical properties and degradation monitoring. Bioprinting **32**, e00288 (2023). 10.1016/j.bprint.2023.e00288

[161] P.G. Saiz, A. Reizabal, J.L. Vilas-Vilela, S. Lanceros-Mendez, P.D. Dalton, Thermochromic responses on melt electrowritten poly(ϵ-caprolactone) microstructures. ACS Appl. Polym. Mater. **5**(6), 3883–3887 (2023). 10.1021/acsapm.3c00427

[162] W. Peng, J. Yin, X. Zhang, Y. Shi, G. Che et al., 4D printed shape memory anastomosis ring with controllable shape transformation and degradation. Adv. Funct. Mater. **33**(20), 2214505 (2023). 10.1002/adfm.202214505

[163] Z.-W. Ren, Z.-Y. Wang, Y.-W. Ding, J.-W. Dao, H.-R. Li et al., Polyhydroxyalkanoates: the natural biopolyester for future medical innovations. Biomater. Sci. **11**(18), 6013–6034 (2023). 10.1039/d3bm01043k 37522312 10.1039/d3bm01043k

[164] Y.-W. Ding, Y. Li, Z.-W. Zhang, J.-W. Dao, D.-X. Wei, Hydrogel forming microneedles loaded with VEGF and Ritlecitinib/polyhydroxyalkanoates nanoparticles for mini-invasive androgenetic alopecia treatment. Bioact. Mater. **38**, 95–108 (2024). 10.1016/j.bioactmat.2024.04.020 38699241 10.1016/j.bioactmat.2024.04.020 PMC11061199

[165] M.Z. Gładysz, D. Ubels, M. Koch, A. Amirsadeghi, F. Alleblas et al., Melt electrowriting of polyhydroxyalkanoates for enzymatically degradable scaffolds. Adv. Healthc. Mater. **14**(6), 2401504 (2025). 10.1002/adhm.202401504 39533454 10.1002/adhm.202401504 PMC11874678

[166] D. Sang, X. Luo, J. Liu, Biological interaction and imaging of ultrasmall gold nanoparticles. Nano-Micro Lett. **16**(1), 44 (2023). 10.1007/s40820-023-01266-4 10.1007/s40820-023-01266-4 PMC10695915 38047998

[167] E. Navarro-Palomares, P. González-Saiz, C. Renero-Lecuna, R. Martín-Rodríguez, F. Aguado et al., Dye-doped biodegradable nanoparticle SiO_2_ coating on zinc- and iron-oxide nanoparticles to improve biocompatibility and for *in vivo* imaging studies. Nanoscale **12**(10), 6164–6175 (2020). 10.1039/C9NR08743E 32133463 10.1039/c9nr08743e

[168] D. Rahmatabadi, M.A. Yousefi, S. Shamsolhodaei, M. Baniassadi, K. Abrinia et al., 4D printing of polyethylene glycol-grafted carbon nanotube-reinforced polyvinyl chloride–polycaprolactone composites for enhanced shape recovery and thermomechanical performance. Adv. Intell. Syst. (2025). 10.1002/aisy.202500113

[169] G. Constante, I. Apsite, H. Alkhamis, M. Dulle, M. Schwarzer et al., 4D biofabrication using a combination of 3D printing and melt-electrowriting of shape-morphing polymers. ACS Appl. Mater. Interfaces **13**(11), 12767–12776 (2021). 10.1021/acsami.0c18608 33389997 10.1021/acsami.0c18608

[170] J. Uribe-Gomez, A. Posada-Murcia, A. Shukla, M. Ergin, G. Constante et al., Shape-morphing fibrous hydrogel/elastomer bilayers fabricated by a combination of 3D printing and melt electrowriting for muscle tissue regeneration. ACS Appl. Bio Mater. **4**(2), 1720–1730 (2021). 10.1021/acsabm.0c01495 10.1021/acsabm.0c01495 35014518

[171] X. Wang, Y. He, Y. Liu, J. Leng, Advances in shape memory polymers: remote actuation, multi-stimuli control, 4D printing and prospective applications. Mater. Sci. Eng. R. Rep. **151**, 100702 (2022). 10.1016/j.mser.2022.100702

[172] D. Chen, Q. Liu, Z. Han, J. Zhang, H. Song et al., 4D printing strain self-sensing and temperature self-sensing integrated sensor-actuator with bioinspired gradient gaps. Adv. Sci. **7**(13), 2000584 (2020). 10.1002/advs.202000584 10.1002/advs.202000584 PMC7341108 32670768

[173] M.N.I. Shiblee, K. Ahmed, M. Kawakami, H. Furukawa, 4D printing of shape-memory hydrogels for soft-robotic functions. Adv. Mater. Technol. **4**(8), 1900071 (2019). 10.1002/admt.201900071

[174] R. Qu, D. Zhou, T. Guo, W. He, C. Cui et al., 4D printing of shape memory inferior vena *cava* filters based on copolymer of poly(glycerol sebacate) acrylate-*co*-hydroxyethyl methacrylate (PGSA-HEMA). Mater. Des. **225**, 111556 (2023). 10.1016/j.matdes.2022.111556

[175] X. Peng, S. Wu, X. Sun, L. Yue, S.M. Montgomery et al., 4D printing of freestanding liquid crystal elastomers *via* hybrid additive manufacturing. Adv. Mater. **34**(39), e2204890 (2022). 10.1002/adma.202204890 35962737 10.1002/adma.202204890

[176] H. Lu, M. Lei, C. Zhao, Y. Yao, J. Gou et al., Controlling Au electrode patterns for simultaneously monitoring electrical actuation and shape recovery in shape memory polymer. Compos. B Eng. **80**, 37–42 (2015). 10.1016/j.compositesb.2015.05.039

[177] A. Servant, V. Leon, D. Jasim, L. Methven, P. Limousin et al., Graphene-based electroresponsive scaffolds as polymeric implants for on-demand drug delivery. Adv. Healthc. Mater. **3**(8), 1334–1343 (2014). 10.1002/adhm.201400016 24799416 10.1002/adhm.201400016

[178] S. Anand, C.A. Müller, BNørrehvedde. Jensen, M. Chen, Embracing remote fields as the fourth dimension of tissue biofabrication. Adv. Funct. Mater. **34**(32), 2401654 (2024). 10.1002/adfm.202401654

[179] C. Neudorfer, C.T. Chow, A. Boutet, A. Loh, J. Germann et al., Kilohertz-frequency stimulation of the nervous system: a review of underlying mechanisms. Brain Stimul. **14**(3), 513–530 (2021). 10.1016/j.brs.2021.03.008 33757930 10.1016/j.brs.2021.03.008

[180] Y.S. Lui, W.T. Sow, L.P. Tan, Y. Wu, Y. Lai et al., 4D printing and stimuli-responsive materials in biomedical aspects. Acta Biomater. **92**, 19–36 (2019). 10.1016/j.actbio.2019.05.005 31071476 10.1016/j.actbio.2019.05.005

[181] J. Liu, Y. Gao, H. Wang, R. Poling-Skutvik, C.O. Osuji et al., Shaping and locomotion of soft robots using filament actuators made from liquid crystal elastomer–carbon nanotube composites. Adv. Intell. Syst. **2**(6), 1900163 (2020). 10.1002/aisy.201900163

[182] L. Ceamanos, Z. Kahveci, M. López-Valdeolivas, D. Liu, D.J. Broer et al., Four-dimensional printed liquid crystalline elastomer actuators with fast photoinduced mechanical response toward light-driven robotic functions. ACS Appl. Mater. Interfaces **12**(39), 44195–44204 (2020). 10.1021/acsami.0c13341 32885661 10.1021/acsami.0c13341

[183] Y. Deng, F. Zhang, M. Jiang, Y. Liu, H. Yuan et al., Programmable 4d printing of photoactive shape memory composite structures. ACS Appl. Mater. Interfaces **14**(37), 42568–42577 (2022). 10.1021/acsami.2c13982 36097702 10.1021/acsami.2c13982

[184] S. Tasmim, Z. Yousuf, F.S. Rahman, E. Seelig, A.J. Clevenger et al., Liquid crystal elastomer based dynamic device for urethral support: potential treatment for stress urinary incontinence. Biomaterials **292**, 121912 (2023). 10.1016/j.biomaterials.2022.121912 36434829 10.1016/j.biomaterials.2022.121912 PMC9772118

[185] J. Wang, Y. Xu, D. Zhang, W. Liu, Z. Li et al., Multifunctional, NIR light-responsive, 4D printable polyurethane/polydopamine nanocomposite. Polymer **324**, 128214 (2025). 10.1016/j.polymer.2025.128214

[186] A. Zolfagharian, A. Kaynak, S.Y. Khoo, A. Kouzani, Pattern-driven 4d printing. Sens. Actuators, A Phys. **274**, 231–243 (2018). 10.1016/j.sna.2018.03.034

[187] H. Cui, S. Miao, T. Esworthy, S.-J. Lee, X. Zhou et al., A novel near-infrared light responsive 4D printed nanoarchitecture with dynamically and remotely controllable transformation. Nano Res. **12**, 1381–1388 (2019). 10.1007/s12274-019-2340-9 33312444 10.1007/s12274-019-2340-9 PMC7731938

[188] S. Chen, T. Takata, K. Domen, Particulate photocatalysts for overall water splitting. Nat. Rev. Mater. **2**(10), 17050 (2017). 10.1038/natrevmats.2017.50

[189] S. Johannsmeier, P. Heeger, M. Terakawa, S. Kalies, A. Heisterkamp et al., Gold nanoparticle-mediated laser stimulation induces a complex stress response in neuronal cells. Sci. Rep. **8**, 6533 (2018). 10.1038/s41598-018-24908-9 29695746 10.1038/s41598-018-24908-9 PMC5917034

[190] H. Sies, V.V. Belousov, N.S. Chandel, M.J. Davies, D.P. Jones et al., Defining roles of specific reactive oxygen species (ROS) in cell biology and physiology. Nat. Rev. Mol. Cell Biol. **23**(7), 499–515 (2022). 10.1038/s41580-022-00456-z 35190722 10.1038/s41580-022-00456-z

[191] Y. Kim, X. Zhao, Magnetic soft materials and robots. Chem. Rev. **122**(5), 5317–5364 (2022). 10.1021/acs.chemrev.1c00481 35104403 10.1021/acs.chemrev.1c00481 PMC9211764

[192] F. Zhang, L. Wang, Z. Zheng, Y. Liu, J. Leng, Magnetic programming of 4D printed shape memory composite structures. Compos. Part A Appl. Sci. Manuf. **125**, 105571 (2019). 10.1016/j.compositesa.2019.105571

[193] H. Liu, F. Wang, W. Wu, X. Dong, L. Sang, 4D printing of mechanically robust PLA/TPU/Fe_3_O_4_ magneto-responsive shape memory polymers for smart structures. Compos. Part B Eng. **248**, 110382 (2023). 10.1016/j.compositesb.2022.110382

[194] V. Walsh, A. Cowey, Transcranial magnetic stimulation and cognitive neuroscience. Nat. Rev. Neurosci. **1**(1), 73–80 (2000). 10.1038/35036239 11252771 10.1038/35036239

[195] A.T. Sack, D.E.J. Linden, Combining transcranial magnetic stimulation and functional imaging in cognitive brain research: possibilities and limitations. Brain Res. Brain Res. Rev. **43**(1), 41–56 (2003). 10.1016/s0165-0173(03)00191-7 14499461 10.1016/s0165-0173(03)00191-7

[196] N.G. Horton, K. Wang, D. Kobat, C.G. Clark, F.W. Wise et al., *In vivo* three-photon microscopy of subcortical structures within an intact mouse brain. Nat. Photonics **7**(3), 205–209 (2013). 10.1038/nphoton.2012.336 24353743 10.1038/nphoton.2012.336 PMC3864872

[197] M. Hallett, Transcranial magnetic stimulation: a primer. Neuron **55**(2), 187–199 (2007). 10.1016/j.neuron.2007.06.026 17640522 10.1016/j.neuron.2007.06.026

[198] J.H. Young, M.-T. Wang, I.A. Brezovich, Frequency/depth-penetration considerations in hyperthermia by magnetically induced currents. Electron. Lett. **16**(10), 358–359 (1980). 10.1049/el:19800255

[199] S. Das Barman, A.W. Reza, N. Kumar, M.E. Karim, A.B. Munir, Wireless powering by magnetic resonant coupling: recent trends in wireless power transfer system and its applications. Renew. Sustain. Energy Rev. **51**, 1525–1552 (2015). 10.1016/j.rser.2015.07.031

[200] S. Parimita, A. Kumar, H. Krishnaswamy, P. Ghosh, Solvent triggered shape morphism of 4D printed hydrogels. J. Manuf. Process. **85**, 875–884 (2023). 10.1016/j.jmapro.2022.11.065

[201] M. Jamal, S.S. Kadam, R. Xiao, F. Jivan, T.-M. Onn et al., Bio-origami hydrogel scaffolds composed of photocrosslinked PEG bilayers. Adv. Healthc. Mater. **2**(8), 1142–1150 (2013). 10.1002/adhm.201200458 23386382 10.1002/adhm.201200458

[202] P. Imrie, J. Jin, Polymer 4D printing: advanced shape-change and beyond. J. Polym. Sci. **60**(2), 149–174 (2022). 10.1002/pol.20210718

[203] Y. Hu, Z. Wang, D. Jin, C. Zhang, R. Sun et al., Botanical-inspired 4d printing of hydrogel at the microscale. Adv. Funct. Mater. **30**(4), 1907377 (2020). 10.1002/adfm.201907377

[204] D. Kim, K.-H. Kim, Y.-S. Yang, K.-S. Jang, S. Jeon et al., 4D printing and simulation of body temperature-responsive shape-memory polymers for advanced biomedical applications. Int. J. Bioprinting (2024). 10.36922/ijb.3035

[205] S. Choudhury, A. Joshi, V.S. Baghel, G.K. Ananthasuresh, S. Asthana et al., Design-encoded dual shape-morphing and shape-memory in 4d printed polymer parts toward cellularized vascular grafts. J. Mater. Chem. B **12**(23), 5678–5689 (2024). 10.1039/D4TB00437J 38747702 10.1039/d4tb00437j

[206] F. Tang, A. Ding, Y. Xu, Y. Ye, L. Li et al., Gene and photothermal combination therapy: principle, materials, and amplified anticancer intervention. Small **20**(6), 2307078 (2024). 10.1002/smll.202307078 10.1002/smll.202307078 37775950

[207] S.H. Beachy, E.A. Repasky, Toward establishment of temperature thresholds for immunological impact of heat exposure in humans. Int. J. Hyperthermia **27**(4), 344–352 (2011). 10.3109/02656736.2011.562873 21591898 10.3109/02656736.2011.562873 PMC3620730

[208] O. Feuerstein, K. Zeichner, C. Imbari, Z. Ormianer, N. Samet et al., Temperature changes in dental implants following exposure to hot substances in an *ex vivo* model. Clin. Oral Implants Res. **19**(6), 629–633 (2008). 10.1111/j.1600-0501.2007.01502.x 18371098 10.1111/j.1600-0501.2007.01502.x

[209] Y. Zhu, K. Deng, J. Zhou, C. Lai, Z. Ma et al., Shape-recovery of implanted shape-memory devices remotely triggered *via* image-guided ultrasound heating. Nat. Commun. **15**(1), 1123 (2024). 10.1038/s41467-024-45437-2 38321028 10.1038/s41467-024-45437-2 PMC10847440

[210] J. Wang, Z. Wang, Z. Song, L. Ren, Q. Liu, Programming multistage shape memory and variable recovery force with 4D printing parameters. Adv. Mater. Technol. **4**(11), 1900535 (2019). 10.1002/admt.201900535

[211] S. Nam, E. Pei, The influence of shape changing behaviors from 4D printing through material extrusion print patterns and infill densities. Materials **13**(17), 3754 (2020). 10.3390/ma13173754 32854309 10.3390/ma13173754 PMC7503952

[212] A.R. Rajkumar, K. Shanmugam, Additive manufacturing-enabled shape transformations *via* FFF 4d printing. J. Mater. Res. **33**(24), 4362–4376 (2018). 10.1557/jmr.2018.397

[213] L. Huang, R. Jiang, J. Wu, J. Song, H. Bai et al., Ultrafast digital printing toward 4D shape changing materials. Adv. Mater. **29**(7), 1605390 (2017). 10.1002/adma.201605390 10.1002/adma.201605390 27936286

[214] Y. Wu, G. Guo, Z. Wei, J. Qian, Programming soft shape-morphing systems by harnessing strain mismatch and snap-through bistability: a review. Materials **15**(7), 2397 (2022). 10.3390/ma15072397 35407728 10.3390/ma15072397 PMC8999758

[215] D.J. Roach, X. Sun, X. Peng, F. Demoly, K. Zhou et al., 4D printed multifunctional composites with cooling-rate mediated tunable shape morphing. Adv. Funct. Mater. **32**(36), 2203236 (2022). 10.1002/adfm.202203236

[216] Z.J. Wang, C.N. Zhu, W. Hong, Z.L. Wu, Q. Zheng, Programmed planar-to-helical shape transformations of composite hydrogels with bioinspired layered fibrous structures. J. Mater. Chem. B **4**(44), 7075–7079 (2016). 10.1039/C6TB02178F 32263643 10.1039/c6tb02178f

[217] H. Wang, J. Guo, Recent advances in 4d printing hydrogel for biological interfaces. Int. J. Mater. Form. **16**(5), 55 (2023). 10.1007/s12289-023-01778-9

[218] M.R. Vinciguerra, D.K. Patel, W. Zu, M. Tavakoli, C. Majidi et al., Multimaterial printing of liquid crystal elastomers with integrated stretchable electronics. ACS Appl. Mater. Interfaces **15**(20), 24777–24787 (2023). 10.1021/acsami.2c23028 37163362 10.1021/acsami.2c23028 PMC10214374

[219] J.W. Boley, W.M. van Rees, C. Lissandrello, M.N. Horenstein, R.L. Truby et al., Shape-shifting structured lattices *via* multimaterial 4D printing. Proc. Natl. Acad. Sci. U. S. A. **116**(42), 20856–20862 (2019). 10.1073/pnas.1908806116 31578256 10.1073/pnas.1908806116 PMC6800333

[220] O. Bas, B. Gorissen, S. Luposchainsky, T. Shabab, K. Bertoldi et al., Ultrafast, miniature soft actuators. Multifunct. Mater. **4**(4), 045001 (2021). 10.1088/2399-7532/ac2faf

[221] Y. Wang, X. Li, An accurate finite element approach for programming 4D-printed self-morphing structures produced by fused deposition modeling. Mech. Mater. **151**, 103628 (2020). 10.1016/j.mechmat.2020.103628

[222] S. Timoshenko, Analysis of bi-metal thermostats. J. Opt. Soc. Am. **11**(3), 233 (1925). 10.1364/josa.11.000233

[223] Y. Wu, X. Hao, R. Xiao, J. Lin, Z.L. Wu et al., Controllable bending of bi-hydrogel strips with differential swelling. Acta Mech. Solida Sin. **32**(5), 652–662 (2019). 10.1007/s10338-019-00106-6

[224] L. Li, P. Wang, H. Liang, J. Jin, Y. Zhang et al., Design of a Haversian system-like gradient porous scaffold based on triply periodic minimal surfaces for promoting bone regeneration. J. Adv. Res. **54**, 89–104 (2023). 10.1016/j.jare.2023.01.004 36632888 10.1016/j.jare.2023.01.004 PMC10704082

[225] C.-Y. Cheng, H. Xie, Z.-Y. Xu, L. Li, M.-N. Jiang et al., 4D printing of shape memory aliphatic copolyester *via* UV-assisted FDM strategy for medical protective devices. Chem. Eng. J. **396**, 125242 (2020). 10.1016/j.cej.2020.125242

[226] Z. Li, P. Yan, H. Wang, Y. Zhang, J. Kong et al., Dynamic bonds reinforced polyamide elastomer for biomedical orthosis. Adv. Sci. **12**(30), e04395 (2025). 10.1002/advs.202504395 10.1002/advs.202504395 PMC12376624 40391678

[227] M. Ramezani, Z. Mohd Ripin, 4D printing in biomedical engineering: advancements, challenges, and future directions. J. Funct. Biomater. **14**(7), 347 (2023). 10.3390/jfb14070347 37504842 10.3390/jfb14070347 PMC10381284

[228] A. Mandal, K. Chatterjee, 4D printing for biomedical applications. J. Mater. Chem. B **12**(12), 2985–3005 (2024). 10.1039/d4tb00006d 38436200 10.1039/d4tb00006d

[229] X. Chen, S. Han, W. Wu, Z. Wu, Y. Yuan et al., Harnessing 4D printing bioscaffolds for advanced orthopedics. Small **18**(36), e2106824 (2022). 10.1002/smll.202106824 35060321 10.1002/smll.202106824

[230] N. Wang, Review of cellular mechanotransduction. J. Phys. D Appl. Phys. **50**(23), 233002 (2017). 10.1088/1361-6463/aa6e18 29097823 10.1088/1361-6463/aa6e18 PMC5662120

[231] A. Melocchi, M. Uboldi, M. Cerea, A. Foppoli, A. Maroni et al., Shape memory materials and 4D printing in pharmaceutics. Adv. Drug Deliv. Rev. **173**, 216–237 (2021). 10.1016/j.addr.2021.03.013 33774118 10.1016/j.addr.2021.03.013

[232] U. Aizarna-Lopetegui, S.C. Bittinger, N. Álvarez, M. Henriksen-Lacey, D. de Jimenez Aberasturi, Stimuli-responsive hybrid materials for 4D *in vitro* tissue models. Mater. Today Bio. **33**, 102035 (2025). 10.1016/j.mtbio.2025.102035 10.1016/j.mtbio.2025.102035 PMC12275134 40688677

[233] W.J. Hendrikson, J. Rouwkema, F. Clementi, C.A. van Blitterswijk, S. Farè et al., Towards 4D printed scaffolds for tissue engineering: exploiting 3D shape memory polymers to deliver time-controlled stimulus on cultured cells. Biofabrication **9**(3), 031001 (2017). 10.1088/1758-5090/aa8114 28726680 10.1088/1758-5090/aa8114

[234] Y. Wang, H. Cui, Y. Wang, C. Xu, T.J. Esworthy et al., 4D printed cardiac construct with aligned myofibers and adjustable curvature for myocardial regeneration. ACS Appl. Mater. Interfaces **13**(11), 12746–12758 (2021). 10.1021/acsami.0c17610 33405502 10.1021/acsami.0c17610 PMC9554838

[235] H. Cui, C. Liu, T. Esworthy, Y. Huang, Z.-X. Yu et al., 4D physiologically adaptable cardiac patch: a 4-month *in vivo* study for the treatment of myocardial infarction. Sci. Adv. **6**(26), eabb5067 (2020). 10.1126/sciadv.abb5067 32637623 10.1126/sciadv.abb5067 PMC7314523

[236] M. Montgomery, S. Ahadian, L. Davenport Huyer, M. Lo Rito, R.A. Civitarese et al., Flexible shape-memory scaffold for minimally invasive delivery of functional tissues. Nat. Mater. **16**(10), 1038–1046 (2017). 10.1038/nmat4956 28805824 10.1038/nmat4956

[237] Y. Chen, Y. Zhou, Z. Hu, W. Lu, Z. Li et al., Gelatin-based metamaterial hydrogel films with high conformality for ultra-soft tissue monitoring. Nano-Micro Lett. **16**(1), 34 (2023). 10.1007/s40820-023-01225-z 10.1007/s40820-023-01225-z PMC10686972 38019305

[238] X. Han, Q. Saiding, X. Cai, Y. Xiao, P. Wang et al., Intelligent vascularized 3D/4D/5D/6D-printed tissue scaffolds. Nano-Micro Lett. **15**(1), 239 (2023). 10.1007/s40820-023-01187-2 10.1007/s40820-023-01187-2 PMC10618155 37907770

[239] C. Cui, D.-O. Kim, M.Y. Pack, B. Han, L. Han et al., 4D printing of self-folding and cell-encapsulating 3D microstructures as scaffolds for tissue-engineering applications. Biofabrication **12**(4), 045018 (2020). 10.1088/1758-5090/aba502 32650325 10.1088/1758-5090/aba502

[240] A. Weekes, J.M. Wasielewska, N. Pinto, J. Jenkins, J. Patel et al., Harnessing the regenerative potential of fetal mesenchymal stem cells and endothelial colony-forming cells in the biofabrication of tissue-engineered vascular grafts (TEVGs). J. Tissue Eng. Regen. Med. **2024**(1), 8707377 (2024). 10.1155/2024/8707377 40225752 10.1155/2024/8707377 PMC11919237

[241] G. Größbacher, M. Bartolf-Kopp, C. Gergely, P.N. Bernal, S. Florczak et al., Volumetric printing across melt electrowritten scaffolds fabricates multi-material living constructs with tunable architecture and mechanics. Adv. Mater. **35**(32), 2300756 (2023). 10.1002/adma.202300756 10.1002/adma.202300756 37099802

[242] C. Shen, A. Shen, 4D printing: innovative solutions and technological advances in orthopedic repair and reconstruction, personalized treatment and drug delivery. Biomed. Eng. Online **24**(1), 5 (2025). 10.1186/s12938-025-01334-3 39838448 10.1186/s12938-025-01334-3 PMC11748259

[243] D. You, G. Chen, C. Liu, X. Ye, S. Wang et al., 4D printing of multi-responsive membrane for accelerated *in vivo* bone healing *via* remote regulation of stem cell fate. Adv. Funct. Mater. **31**(40), 2103920 (2021). 10.1002/adfm.202103920

[244] B. Hermenegildo, C. Ribeiro, L. Pérez-Álvarez, J.L. Vilas, D.A. Learmonth et al., Hydrogel-based magnetoelectric microenvironments for tissue stimulation. Colloids Surf. B Biointerfaces **181**, 1041–1047 (2019). 10.1016/j.colsurfb.2019.06.023 31382332 10.1016/j.colsurfb.2019.06.023

[245] A. Ding, S.J. Lee, R. Tang, K.L. Gasvoda, F. He et al., 4d cell-condensate bioprinting. Small **18**(36), 2202196 (2022). 10.1002/smll.202202196 10.1002/smll.202202196 PMC9463124 35973946

[246] C. Lin, L. Zhang, Y. Liu, L. Liu, J. Leng, 4D printing of personalized shape memory polymer vascular stents with negative Poisson’s ratio structure: a preliminary study. Sci. China Technol. Sci. **63**(4), 578–588 (2020). 10.1007/s11431-019-1468-2

[247] Y. Deng, B. Yang, F. Zhang, Y. Liu, J. Sun et al., 4D printed orbital stent for the treatment of enophthalmic invagination. Biomaterials **291**, 121886 (2022). 10.1016/j.biomaterials.2022.121886 36356472 10.1016/j.biomaterials.2022.121886

[248] C. Lin, Z. Huang, Q. Wang, Z. Zou, W. Wang et al., Mass-producible near-body temperature-triggered 4D printed shape memory biocomposites and their application in biomimetic intestinal stents. Compos. B Eng. **256**, 110623 (2023). 10.1016/j.compositesb.2023.110623

[249] C. Lin, L. Liu, Y. Liu, J. Leng, 4D printing of bioinspired absorbable left atrial appendage occluders: a proof-of-concept study. ACS Appl. Mater. Interfaces **13**(11), 12668–12678 (2021). 10.1021/acsami.0c17192 33397086 10.1021/acsami.0c17192

[250] C. Zhang, D. Cai, P. Liao, J.-W. Su, H. Deng et al., 4D printing of shape-memory polymeric scaffolds for adaptive biomedical implantation. Acta Biomater. **122**, 101–110 (2021). 10.1016/j.actbio.2020.12.042 33359298 10.1016/j.actbio.2020.12.042 PMC7897283

[251] H. Pandey, S.S. Mohol, R. Kandi, 4D printing of tracheal scaffold using shape-memory polymer composite. Mater. Lett. **329**, 133238 (2022). 10.1016/j.matlet.2022.133238

[252] C. Ni, D. Chen, Y. Yin, X. Wen, X. Chen et al., Shape memory polymer with programmable recovery onset. Nature **622**(7984), 748–753 (2023). 10.1038/s41586-023-06520-8 37704734 10.1038/s41586-023-06520-8

[253] D. Mukherjee, J. Li, D. Spinosa, Aortic aneurysm management results through one year with a conformable neck sealing endograft and preemptive sac embolization with shape memory polymer devices. J. Vasc. Surg. Cases Innov. Tech. **11**(1), 101656 (2025). 10.1016/j.jvscit.2024.101656 39654957 10.1016/j.jvscit.2024.101656 PMC11626527

[254] Y. Woon, K. Hyun, W. Lee, K. Hwan, Comparative analysis of temperature-responsive hydrogel (PF 72) for postoperative pain after bimaxillary surgery: a retro-spective study. Aesthet. Plast. Surg. **48**(7), 1271–1275 (2024). 10.1007/s00266-023-03846-6 10.1007/s00266-023-03846-6 38326500

[255] M. Zhang, W. Jiang, Z.-X. Wang, Z.-M. Zhou, Using shape-memory alloy staples to treat comminuted manubrium sterni fractures: a case report. World J. Clin. Cases **11**(30), 7386–7392 (2023). 10.12998/wjcc.v11.i30.7386 37969455 10.12998/wjcc.v11.i30.7386 PMC10643072

[256] K. Somszor, O. Bas, F. Karimi, T. Shabab, N.T. Saidy et al., Personalized, mechanically strong, and biodegradable coronary artery stents *via* melt electrowriting. ACS Macro Lett. **9**(12), 1732–1739 (2020). 10.1021/acsmacrolett.0c00644 35653675 10.1021/acsmacrolett.0c00644

[257] C. Wischke, A.T. Neffe, S. Steuer, A. Lendlein, Evaluation of a degradable shape-memory polymer network as matrix for controlled drug release. J. Control. Release **138**(3), 243–250 (2009). 10.1016/j.jconrel.2009.05.027 19470395 10.1016/j.jconrel.2009.05.027

[258] M. Jahangiri, A.E. Kalajahi, M. Rezaei, M. Bagheri, Shape memory hydroxypropyl cellulose-g-poly (ε-caprolactone) networks with controlled drug release capabilities. J. Polym. Res. **26**(6), 136 (2019). 10.1007/s10965-019-1798-1

[259] X. Wang, J. Zeng, D. Gan, K. Ling, M. He et al., Recent strategies and advances in hydrogel-based delivery platforms for bone regeneration. Nano-Micro Lett. **17**(1), 73 (2024). 10.1007/s40820-024-01557-4 10.1007/s40820-024-01557-4 PMC11602938 39601916

[260] Y. Wang, Y. Miao, J. Zhang, J.P. Wu, T.B. Kirk et al., Three-dimensional printing of shape memory hydrogels with internal structure for drug delivery. Mater. Sci. Eng., C **84**, 44–51 (2018). 10.1016/j.msec.2017.11.025 10.1016/j.msec.2017.11.025 29519442

[261] A. Sadraei, S.M. Naghib, 4D printing of physical stimuli-responsive hydrogels for localized drug delivery and tissue engineering. Polym. Rev. **65**(1), 104–168 (2025). 10.1080/15583724.2024.2427184

[262] A. Gazzaniga, A. Foppoli, M. Cerea, L. Palugan, M. Cirilli et al., Towards 4D printing in pharmaceutics. International Journal of Pharmaceutics: X **5**, 100171 (2023). 10.1016/j.ijpx.2023.100171 36876052 10.1016/j.ijpx.2023.100171 PMC9982600

[263] L. Keßler, Z. Mirzaei, J.C. Kade, R. Luxenhofer, Highly porous and drug-loaded amorphous solid dispersion microfiber scaffolds of indomethacin prepared by melt electrowriting. ACS Appl. Polym. Mater. **5**(1), 913–922 (2023). 10.1021/acsapm.2c01845

[264] J. Ren, R. Murray, C.S. Wong, J. Qin, M. Chen et al., Development of 3D printed biodegradable mesh with antimicrobial properties for pelvic organ prolapse. Polymers **14**(4), 763 (2022). 10.3390/polym14040763 35215676 10.3390/polym14040763 PMC8877663

[265] A. Mathew, B.L. Devlin, D. Singh, N.C. Paxton, M.A. Woodruff, Improving infection resistance in tissue engineered scaffolds for tensile applications using vancomycin-embedded melt electrowritten scaffolds. Macromol. Mater. Eng. **308**(10), 2300168 (2023). 10.1002/mame.202300168

[266] J.P. Martins, E.T. da Silva, A.A. Fernandes, S. Costa de Oliveira, Three-dimensional melted electrowriting drug coating fibers for the prevention of device-associated infections: a pilot study. Bioengineering **11**(7), 636 (2024). 10.3390/bioengineering11070636 39061718 10.3390/bioengineering11070636 PMC11273671

[267] E. Hewitt, S. Mros, M. McConnell, J.D. Cabral, A. Ali, Melt-electrowriting with novel milk protein/PCL biomaterials for skin regeneration. Biomed. Mater. **14**(5), 055013 (2019). 10.1088/1748-605X/ab3344 31318339 10.1088/1748-605X/ab3344

[268] J. Bai, H. Wang, W. Gao, F. Liang, Z. Wang et al., Melt electrohydrodynamic 3D printed poly (ε-caprolactone)/polyethylene glycol/roxithromycin scaffold as a potential anti-infective implant in bone repair. Int. J. Pharm. **576**, 118941 (2020). 10.1016/j.ijpharm.2019.118941 31881261 10.1016/j.ijpharm.2019.118941

[269] X. Lai, J. Huang, S. Huang, J. Wang, Y. Zheng et al., Antibacterial and osteogenic dual-functional micronano composite scaffold fabricated *via* melt electrowriting and solution electrospinning for bone tissue engineering. ACS Appl. Mater. Interfaces **16**(29), 37707–37721 (2024). 10.1021/acsami.4c07400 39001812 10.1021/acsami.4c07400

[270] F.-L. He, X. Deng, Y.-Q. Zhou, T.-D. Zhang, Y.-L. Liu et al., Controlled release of antibiotics from poly-ε-caprolactone/polyethylene glycol wound dressing fabricated by direct-writing melt electrospinning. Polym. Adv. Technol. **30**(2), 425–434 (2019). 10.1002/pat.4481

[271] F. van Charante, D. Martínez-Pérez, C. Guarch-Pérez, C. Courtens, A. Sass et al., 3D-printed wound dressings containing a fosmidomycin-derivative prevent *Acinetobacter baumannii* biofilm formation. iScience **26**(9), 107557 (2023). 10.1016/j.isci.2023.107557 37680458 10.1016/j.isci.2023.107557 PMC10480667

[272] T. Xu, J. Gu, J. Meng, L. Du, A. Kumar et al., Melt electrowriting reinforced composite membrane for controlled drug release. J. Mech. Behav. Biomed. Mater. **132**, 105277 (2022). 10.1016/j.jmbbm.2022.105277 35617819 10.1016/j.jmbbm.2022.105277

[273] F. Afghah, N.B. Iyison, A. Nadernezhad, A. Midi, O. Sen et al., 3D fiber reinforced hydrogel scaffolds by melt electrowriting and gel casting as a hybrid design for wound healing. Adv. Healthc. Mater. **11**(11), e2102068 (2022). 10.1002/adhm.202102068 35120280 10.1002/adhm.202102068

[274] G. Cedillo-Servin, A.F. Louro, B. Gamelas, A. Meliciano, A. Zijl et al., Microfiber-reinforced hydrogels prolong the release of human induced pluripotent stem cell-derived extracellular vesicles to promote endothelial migration. Biomater. Adv. **155**, 213692 (2023). 10.1016/j.bioadv.2023.213692 37952463 10.1016/j.bioadv.2023.213692

[275] X. Kong, D. Zhu, Y. Hu, C. Liu, Y. Zhang et al., Melt electrowriting (MEW)-PCL composite three-dimensional exosome hydrogel scaffold for wound healing. Mater. Des. **238**, 112717 (2024). 10.1016/j.matdes.2024.112717

[276] A.R. Mridha, T.R. Dargaville, P.D. Dalton, L. Carroll, M.B. Morris et al., Prevascularized retrievable hybrid implant to enhance function of subcutaneous encapsulated islets. Tissue Eng. Part A **28**(5–6), 212–224 (2022). 10.1089/ten.TEA.2020.0179 33081600 10.1089/ten.TEA.2020.0179

[277] K. Ghosal, P. Sarkar, D. Chakraborty, S. Das, K. Sarkar, Green synthesis of nonisocyanate poly(ester urethanes) from renewable resources and recycled poly(ethylene terephthalate) waste for tissue engineering application. ACS Sustainable Chem. Eng. **11**(37), 13688–13708 (2023). 10.1021/acssuschemeng.3c03566

[278] K. Ghosal, S. Pal, D. Ghosh, K. Jana, K. Sarkar, *In vivo* biocompatible shape memory polyester derived from recycled polycarbonate e-waste for biomedical application. Biomater. Adv. **138**, 212961 (2022). 10.1016/j.bioadv.2022.212961 35913244 10.1016/j.bioadv.2022.212961

[279] L. Pang, N.C. Paxton, J. Ren, F. Liu, H. Zhan et al., Development of mechanically enhanced polycaprolactone composites by a functionalized titanate nanofiller for melt electrowriting in 3D printing. ACS Appl. Mater. Interfaces **12**(42), 47993–48006 (2020). 10.1021/acsami.0c14831 33044824 10.1021/acsami.0c14831

[280] G. Hochleitner, F. Chen, C. Blum, P.D. Dalton, B. Amsden et al., Melt electrowriting below the critical translation speed to fabricate crimped elastomer scaffolds with non-linear extension behaviour mimicking that of ligaments and tendons. Acta Biomater. **72**, 110–120 (2018). 10.1016/j.actbio.2018.03.023 29555458 10.1016/j.actbio.2018.03.023

[281] L. Keßler, R. Luxenhofer, Melt electrowriting of amorphous solid dispersions: influence of drug and plasticizer on rheology and printing performance. Int. J. Pharm. **671**, 125188 (2025). 10.1016/j.ijpharm.2025.125188 39798624 10.1016/j.ijpharm.2025.125188

